# Expression cartography of human tissues using self organizing maps

**DOI:** 10.1186/1471-2105-12-306

**Published:** 2011-07-27

**Authors:** Henry Wirth, Markus Löffler, Martin von Bergen, Hans Binder

**Affiliations:** 1 Interdisciplinary Centre for Bioinformatics of Leipzig University, D-4107 Leipzig, Härtelstr. 16-18, Germany; 2 Helmholtz Centre for Environmental Research, Department of Proteomics, D-04318 Leipzig, Permoserstr. 15, Germany; 3 Institute for Medical Informatics, Statistics and Epidemiology, Universität Leipzig, D-4107 Leipzig, Härtelstr. 16-18, Germany; 4 Leipzig Interdisciplinary Research Cluster of Genetic Factors, Clinical Phenotypes and Environment (LIFE); Universität Leipzig, D-4103 Leipzig, Philipp-Rosenthalstr. 27, Germany; 5 Helmholtz Centre for Environmental Research, Department of Metabolomics, D-04318 Leipzig, Permoserstr. 15, Germany

## Abstract

**Background:**

Parallel high-throughput microarray and sequencing experiments produce vast quantities of multidimensional data which must be arranged and analyzed in a concerted way. One approach to addressing this challenge is the machine learning technique known as self organizing maps (SOMs). SOMs enable a parallel sample- and gene-centered view of genomic data combined with strong visualization and second-level analysis capabilities. The paper aims at bridging the gap between the potency of SOM-machine learning to reduce dimension of high-dimensional data on one hand and practical applications with special emphasis on gene expression analysis on the other hand.

**Results:**

The method was applied to generate a SOM characterizing the whole genome expression profiles of 67 healthy human tissues selected from ten tissue categories (adipose, endocrine, homeostasis, digestion, exocrine, epithelium, sexual reproduction, muscle, immune system and nervous tissues). SOM mapping reduces the dimension of expression data from ten of thousands of genes to a few thousand metagenes, each representing a minicluster of co-regulated single genes. Tissue-specific and common properties shared between groups of tissues emerge as a handful of localized spots in the tissue maps collecting groups of co-regulated and co-expressed metagenes. The functional context of the spots was discovered using overrepresentation analysis with respect to pre-defined gene sets of known functional impact. We found that tissue related spots typically contain enriched populations of genes related to specific molecular processes in the respective tissue. Analysis techniques normally used at the gene-level such as two-way hierarchical clustering are better represented and provide better signal-to-noise ratios if applied to the metagenes. Metagene-based clustering analyses aggregate the tissues broadly into three clusters containing nervous, immune system and the remaining tissues.

**Conclusions:**

The SOM technique provides a more intuitive and informative global view of the behavior of a few well-defined modules of correlated and differentially expressed genes than the separate discovery of the expression levels of hundreds or thousands of individual genes. The program is available as R-package 'oposSOM'.

## 1. Background

DNA microarray and next generation sequencing technologies allow researchers to screen ten thousands of genes for differences in expression between up to hundreds of individuals or experimental conditions of interest. Not only the progressively increasing data throughput of newest array and sequencing technologies challenges data analysis methods but also the increasing availability of large data sets from public data repositories such as gene expression omnibus (http://www.ncbi.nlm.nih.gov/geo/) or array express (http://www.ebi.ac.uk/microarray-as/ae/) with to date hundred thousands of different assays implying large-scale meta-analyses. These resources pose a challenge how to best arrange and to visualize the huge heaps of data in a fashion that enables combination of sample- and gene-centered views on multidimensional expression data to capture the global picture of groups of samples while simultaneously presenting the specific expression pattern within each individual sample.

Self-organizing map (SOM) machine learning was developed by Kohonen about thirty years ago [1]. It projects data from the original high dimensional space to reference vectors of lower dimension. First studies applying SOM to microarray gene expression data were published by Tamayo et al. [2] and Törönen et al. [3]. These and later applications of the SOM method to expression data emphasized either a gene-centered perspective to cluster genes [2] or a sample-centered mode to map individual samples onto the SOM grid enabling the classification of samples into a small number of diagnostic or prognostic groups [4-6]. The SOM method can be configured also in such a way that it combines both, the sample- and gene-centered perspectives [7-9]. This specific approach decodes the expression pattern of ten thousands of genes per sample into a two-dimensional mosaic pattern which allows the sample-to-sample comparison of expression profiles by direct visual inspection.

It has been demonstrated that such SOM displays are featured by several important benefits [7-9]: (i) they provide an individual visual identity for each sample; (ii) they reduce the dimension of the original data; (iii) they preserve the information richness of the molecular portraits allowing the detailed, multivariate explorative comparisons between samples, (iv) they are highly intuitive not-requiring specific knowledge of the underlying algorithmic kernel of the method, and (v) they can be treated as new, complex objects for next level analysis in terms of visual recognition.

SOM-based gene expression analyses have been applied, for example, in studies on cell differentiation and development [10-12], organogenesis [13] and tumor differentiation [14]. It has been demonstrated that SOM analysis can visualize relevant substructures inherent in the data without forcing them into hierarchies and without significant loss of primary information [7]. This intuitive image-based perception clearly promotes the discovery of qualitative relationships between the samples in the absence of an existing hypothesis. The SOM approach also offers new concepts of data analysis based on, e.g., metagene summaries, global entropy estimates and state-space trajectory characteristics [12,13].

Despite its convincing advantages the SOM method is relatively infrequently applied to high-dimensional molecular data compared with alternative approaches such as hierarchical clustering. Possibly, interpretation of SOM mosaic patterns is less trivial and/or unusual for many researchers because it requires basic understanding of details of the method such as the way how expression of real genes transforms into expression profiles of the metagenes. The lack of availability of this information presumably hampers application of SOM-based methods in a wider number of applications. Moreover, standard analysis tasks such as feature selection, significance analysis of differential expression and functional gene set enrichment analysis require the availability of appropriate algorithms and of suited program tools to generate the desired information. Such approaches must consider the specifics of gene expression analysis (e.g., information about the microarray platform used, the probes and the genome of interest, statistical issues and previous knowledge on functional-related gene sets) on one hand, but also the specifics of SOM-machine learning on the other hand.

We strongly advocate in favor of the SOM method. The present publication aims at bridging the gap between the potency of SOM-machine learning to reduce dimension of high-dimensional data on one hand and its availability with special emphasis on gene expression analysis on the other hand. Our approach includes a series of analytical reports which might support interpretation of SOM metagene data (see below) and an available R-program package. Here we focus on the identification and functional interpretation of metagene clusters using gene set overrepresentation analysis, a novel aspect in the context of SOM analysis, and on the comparison of data analysis based on single and on metagenes.

We apply our approach to expression data of human tissues which is well suited as an illustrative example: The selected 67 tissues provide a sufficient large data set of highly diverse expression pattern possessing a complex internal covariance structure. Moreover, the samples are well classified in terms of distinct tissues and tissue categories allowing the clear assignment of expression pattern. The discovery of this human body index data set is also motivated by the argument that tissue-specific RNA expression pattern indicate important clues to the physiological function of the coding genes, suitable as a reference for comparison with diseased tissues. Our analysis thus provides a first step towards a SOM atlas of gene activity in normal human tissues which complements previous work on this objective [15-18].

We address selected methodical aspects of the SOM method which aim at extracting functional information about the expression pattern: Firstly, we complement the gallery of primary SOM images with a number of summary maps characterizing the covariance structure of the data after transformation into latent variables. These summary maps allow extraction of so-called spot-clusters which collect co-expressed metagenes together. This spot-clustering enables to significantly reduce the dimensionality of expression data to a handful of representative expression-modules in an unsupervised fashion. The results of SOM-clustering are compared with the results of alternative methods such as non-negative matrix factorization, hierarchical clustering and sets of correlated genes. Secondly, the detected spot-clusters are linked with biological knowledge to support functional interpretation of the data based on the 'guilt by association' principle. Particularly, we apply gene set overrepresentation analysis to visualization space which is a novel approach to our best knowledge. Thirdly, we analyze the capability of the SOM approach for data filtering and dimension reduction in terms of maintaining representativeness and reduction of noisiness of the data. Finally, we applied SOM analysis in a zoom-in step to a subensemble of tissues to increase the resolution of the method. We use the sample-centered second-level SOM representation to visualize similarity relations between the different tissues and compare the results with independent component analysis.

The main paper is supplemented with additional files which provide the full gallery of SOM-images of human tissues, a detailed methodical section addressing issues such as calibration of microarray raw intensity data to minimize possible artifacts due to systematic biases caused by improper preprocessing [19], the configuration of the SOM-method and additional options of data analysis. We developed our own R-program including all analysis functionalities described below for application of the method. The program is available as CRAN-package 'oposSOM'.

## 2. Results and discussion

### 2.1. Expression maps of human tissues

**Figure 1:**
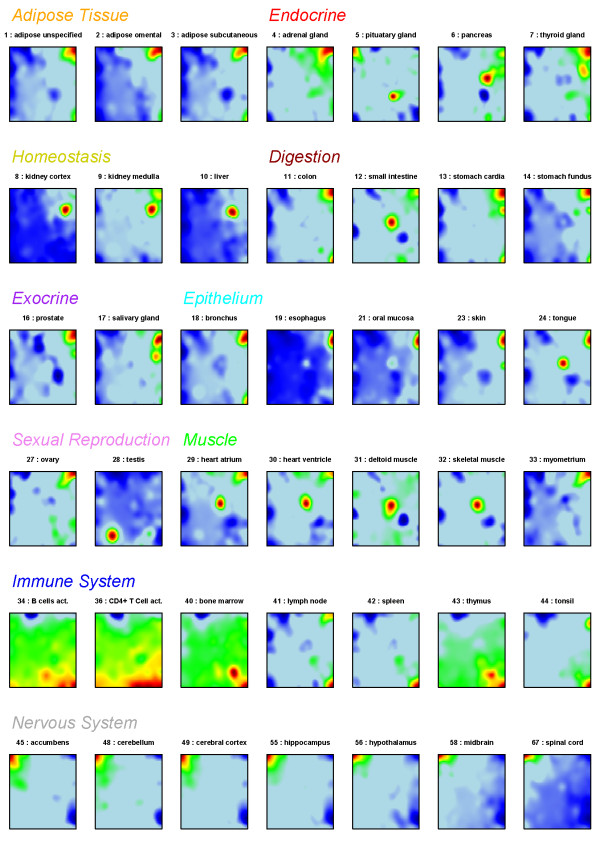
**SOM expression profiles of 42 selected tissues**. The tissues are sorted according to tissue categories in agreement with the classification used in Hornshøj et al. [20]. The color of the heading of each tissue category and the numbering of tissues are used also in the other figures throughout the paper.

Microarray expression data taken from the human body tissue index data set were input into the SOM machine learning algorithm after calibration and normalization of the raw probe intensities as described in the Methods section below and in Additional file 1. Our SOM method transformed the whole genome expression pattern of about 22,000 single genes into one mosaic pattern per tissue studied. Figure 1 shows selected SOM-fingerprints of 42 selected tissues using a 60 × 60 mosaic grid. The collection of SOM profiles of the complete set of 67 tissues is given in Additional file 2 (Additional file 3 shows respective profiles using modified contrasts, please see detailed description in Additional file 1). Each tile of the SOM mosaics refers to one of 3,600 metagenes characterizing the expression landscape of the data set. The metagenes act as representatives of miniclusters of single genes with similar expression profiles. Their number varies from metagene to metagene (see below). The color gradient of the map was chosen to visualize over- or underexpression of the metagenes in the particular tissue compared with the mean expression level of each metagene in the pool of all samples studied: Maroon codes the highest level of gene expression; red, yellow and green indicate intermediate levels and blue corresponds to the lowest level of gene expression. Each individual mosaic exhibits characteristic spatial color patterns serving as fingerprint of the transcriptional activity of the respective tissue sample.

The tissues are grouped into ten categories in accordance with the classification used in Hornshoj et al. [20]. Most of these categories show typical SOM-landscapes which are characterized by red and blue spots at specific positions due to over- and underexpressed metagenes as the most evident features. For example, the profiles of adipose tissues might be identified by the maroon-red overexpression spot in the right upper corner and those of nervous tissues by a similar spot in the top left corner.

**Figure 2:**
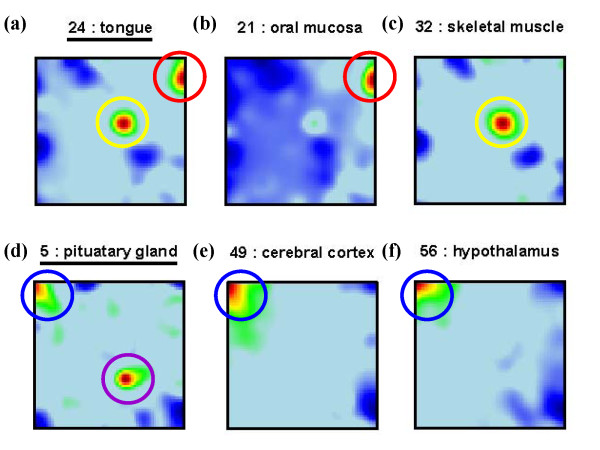
**Specific spots in selected expression profiles: Tongue (panel a), oral mucosa (b), skeletal muscle (c), pituitary gland (d), cerebral cortex (e) and hypothalamus (f).**  The SOM-pattern of tongue (a) shows two spots of upregulated metagenes. One of them is characteristic for mucosa type tissues (b; red circles) and the other one is found in muscle tissues (c, yellow circles). Pituatary gland (d) shows a specific spot for this particular tissue and one which is characteristic for nervous system tissues (e and f, blue circles) as well.

Some tissues combine the characteristic spot pattern of different tissue categories (see Figure 2). For example, the expression fingerprint of tongue (no. 24) shows the typical overexpression spot evident in the profiles of other epithelial tissues (e.g. 21: oral mucosa) but also the spot typically found in muscle tissues (e.g. 32: skeletal muscle). The physiology of tongue tissue as a 'mucosa covered muscle' is thus reflected in the expression profile. Another example is pituatary gland (profile no. 5), an endocrine gland located near hypothalamus: Its SOM landscape shows the upregulated spot found in other nervous system tissues (e.g. cerebral cortex or the adjacent hypothalamus, no. 49 and 56, resp.) in the top left corner, as well as a unique spot in the bottom right area not found in the profiles of other tissues. This spot obviously collects genes which are specifically overexpressed in pituatary gland (see below), whereas the first spot represents a common signature typically found in nervous system samples. Some SOM-fingerprints are outliers in their tissue category: For example, small intestine (no. 12), classified as digestive tissue, shows the overrepresentation pattern of muscle type tissues. This is not surprising as this organ consists of a double layer of smooth muscle. Also myometrium (no. 33), the smooth muscle of the uterus, is classified as muscle. Its SOM expression profile however closely resembles that of endometrium (no. 26) and also of ovary (no. 27), reflecting the common function of these three organs in female reproduction.

In general, SOM fingerprints within a tissue category reveal similar pattern, whereas different tissue types show consistently different expression landscapes. Such differences can be detected, for example, by simple visual inspection of the mosaic pattern of nervous, immune system and endocrine type tissues. Hence, comparison of the SOM-textures allows the straightforward grouping of the tissues into different categories based on differences of their expression patterns.

### 2.2. Metagene characteristics and overexpression spots

**Figure 3:**
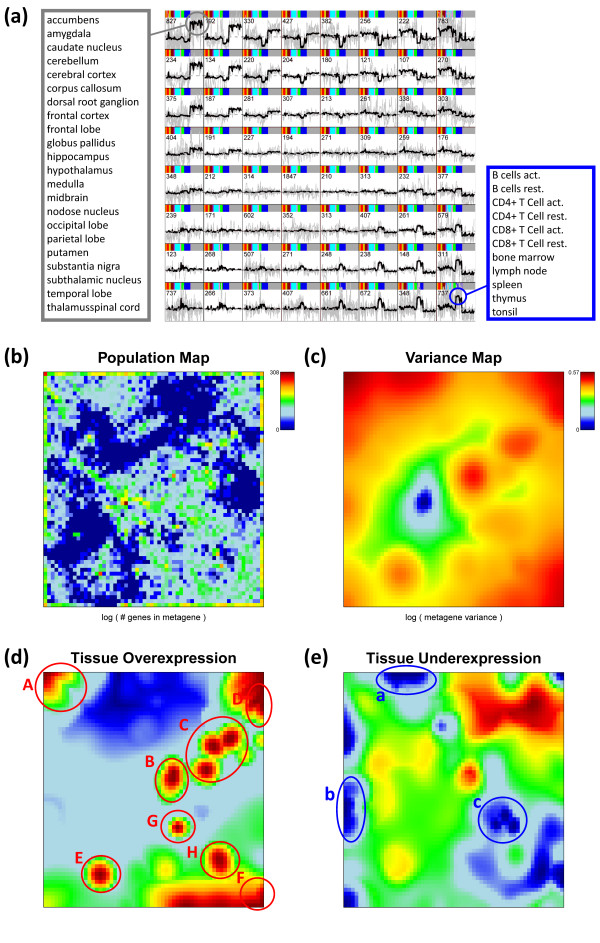
**Metagene characteristics: Metagene expression profiling map of the 67 tissues studied (panel a), population (b), variability (c, Eq. (2)), metagene over- (d) and underexpression (e) maps**. Panel a): Metagene profiles are shown by thick curves whereas thin grey ones show the profiles of associated real genes. The vertical axis is the logged expression change relatively to the mean expression of the selected gene averaged over all tissues. All tiles use the same vertical scale. The number in each tile gives the population of the respective metagene cluster with real genes. The bars color-code the tissue samples (compare with headings in Figure 1). The circles indicate over- and under-expression in selected tissues listed in the boxes (see text). One sees that the metagenes in the top left and the bottom right corner cluster genes strongly overexpressed in nervous (grey circle) and immune system (blue circle) tissues, respectively. Panel d) and e): Red/maroon spots mark overexpression, blue ones underexpression. Selected spots are marked by letters (capital and lower case letters refer to maxima and minima, respectively). They are assigned to different tissues in Table 1.

The metagene expression profiling map in Figure 3a illustrates the systematic character of the alterations of metagene expression between different regions of the SOM. Adjacent metagene profiles show similar profiles whereas more distant metagenes are different and partly show mirror symmetry with respect to the abscissa. In the centre of the map one finds virtually invariant metagenes whereas the profiles along the borders of the map strongly vary between different tissue categories. This distribution of the profiles reflects the fact that SOM machine learning tends to maximally segregate different modes of strong variation on one hand while maximizing the distance between such modes relative to virtually invariant profiles on the other hand. Note also, that the number of real genes per metagene strongly varies throughout the map as indicated by the numbers given in each tile of the metagene expression profiling map.

The metagene expression profiling map uses a smaller number of tiles and thus a coarse grained latticing of the mosaic. The population and variance maps shown in panel b and c of Figure 3 provide information about the number of single genes per metagene minicluster and the variability of the metagene profiles via appropriate color coding using the finer granularity of the mosaic. SOM-machine learning scales the difference between the expression profiles of adjacent metagenes inversely to their population, i.e., adjacent metagene profiles become more similar for highly populated metagenes. This way the method tends to distribute the single genes over as much as possible tiles. The population map reveals that the real genes inhomogeneously distribute among the tiles of the mosaic (Figure 3b). Highly populated metagenes (n_k _> 20, see yellow and red tiles) predominantly group along the edges of the map whereas only a few highly populated tiles are found in its central area. A zone of 'empty' metagenes lacking real genes (n_k _= 0, see dark blue tiles) clusters in four regions halfway between the centre and the edges of the map. The tile of maximum population (n_k _= 308, see the dark brown tile slightly left from the centre of the map) refers to genes with virtually invariant, mostly absent specific expression in all tissues studied (see Additional file 1). These invariant genes give rise to the dark blue spot in the central area of the variance map (Figure 3c). The variance map also reveals that other nearly invariant metagenes cluster around this tile in the central area of the map (see blue and green areas in Figure 3c). Both, invariant and empty metagenes carry essentially no specific information as classification markers in transcriptional profiling. Hence, the tiles occupied by empty and invariant genes form regions not suited for differential expression analysis between the tissues studied.

The more variant and higher populated metagenes reveal an underlying spot like pattern preferentially along the boundaries of the map (red areas in Figure 3c), which agrees with the over- and underexpression spots detected in the SOM mosaics of individual tissues. For an overview about all observed spots we generate two types of integral maps characterizing over- and underexpression, respectively (Figure 3d and 3e). They transfer either the over- or the underexpression spots observed in the individual profile into one master map. The profiles of selected metagenes reveal marked under- and overexpression for distinct tissue types which transform into a characteristic spot patterns (see Figure 1 and Figure 3). For example, the metagenes in the top left corner show overexpression for nervous system and underexpression for immune system tissues whereas the metagenes in the bottom right corner are, in turn, characterized by overexpression in immune system tissues. Table 1 assigns the different spots to the tissue mosaics in which they are observed.

**Table 1:** Functional assignment of tissue specific over- and underexpression spots using the GO-terms biological process/molecular function (see also Figure 3d and 3e).

**Spot **^**a**^	**Over-/underexpressed in tissue **^**a**^	**Biological process/Molecular function (overrepresented genes set) **^**b**^
**A**	Nervous system samples (45-67), pituatary gland(5)	Nervous system development Synaptic transmission Transmission of nerve impuls
**B**	Muscle related: small intestine (12), tongue (24), heart atrium&ventricle (29, 30), muscle (31, 32)	Structural constituent of muscle System process Striated muscle contraction
**C1**	Liver (10), kidney cortex&medulla (8,9)	Substrate specific transporter activity Carboxylic acid metabolic process Organic acid metabolic process
**C2**	Pancreas (6)	Carboxypeptidase activity Carboxylesterase activity Digestion
**D**	Adipose tissue (1-3), epithelium tissue (18-26), ovary (27)	Tissue development Organ development Ectoderm development
**E**	Male reproduction: testis (28)	Sexual reproduction Reproduction Gamete generation
**F**	Immune system samples (34-44)	Immune system process Immune response Defense response
**G**	Pituatary gland(5)	Hormone activity DNA fragmentation during apoptosis Apoptotic nuclear changes
**H**	Bone marrow (40), thymus (43)	Cell cycle process Mitotic cell cycle Cell cycle phase
**a**	Immune system (34-44)	Regulation of axonogenesis Regulation of structural morphogenesis Regulation of neurogenesis
**b**	Various samples without clear assignment, e.g., sexual reproduction and muscle	Microtubule binding Protein maturation Tubulin binding
**c**	Epithelium and muscle tissues	RNA metabolic process Biopolymer metabolic process RNA processing

^a ^Spots are assigned in Figure 3d and 3e. Over- and underexpression spots are labeled using upper and lower case letters, respectively.

^b ^Top-three overrepresented gene sets

### 2.3. Gene set overrepresentation

The SOM assigns mini-clusters of real genes to each metagene represented by a tile in the two-dimensional mosaic pattern. These metagenes collect sets of single genes with similar, mostly highly correlated expression profiles. The correlation and coexpression of the single gene profiles in each spot can be utilized as a simple heuristics with implications for tentative gene function because biological processes are usually governed by coordinated modules of interacting molecules [21]. Application of gene set overrepresentation analysis to the metagene clusters makes use of this 'guilt-by-association' principle which assumes that co-expressed genes are likely to be functionally associated [22,23]. Previous SOM analyses have shown that, indeed, functionally related genes cluster together in the SOM images [7].

For each of the miniclusters we therefore estimate the degree of overrepresentation with respect to pre-defined gene sets using the hypergeometrical (HG-) distribution. It provides an overrepresentation p-value for each metagene and each gene set considered. We visualize the distribution of p-values of each gene sets using the same two-dimensional mosaic as used for the original SOM images and appropriate color-coding. The obtained overrepresentation maps allow identification of metagenes containing an enriched fraction of genes from a selected gene set by visual inspection. Note that this map applies to all samples studied because each of the mini-clusters contains the same genes in all samples used to train the SOM. The overrepresentation map thus reflects the global enrichment pattern of a chosen set of genes in the experimental series studied.

**Figure 4:**
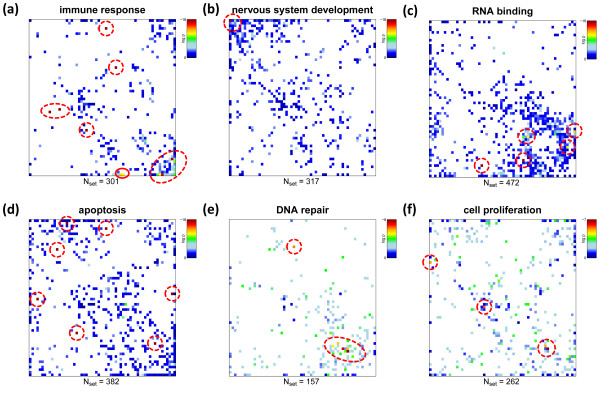
**Overrepresentation maps of six selected gene sets containing between N**_**set **_**= 157 and 472 genes**. Overrepresentation in each tile of the mosaic is calculated in units of log(p_HG_) using the hypergeometrical distribution and color-coded (maroon > red > yellow > green > blue). White areas indicate metagenes not containing genes from the respective set). Strongest overrepresentation of the different gene sets is found in different regions of the SOM (see red circles). Overrepresentation can be concentrated within one or a few adjacent metagenes (e.g. nervous system, panel b) or spread over different disjunct regions of the map (apoptosis, panel d).

Figure 4 shows overrepresentation maps for selected gene sets. Their overrepresentation is usually observed in different regions of the map, for example in the bottom right and top left corner for genes related to 'immune system process' and to the 'transmission of nerve impulse', respectively. The examples also show that overrepresentation is either strongly localized in one region of the map (e.g. for 'nervous system' or, to a less degree, for 'RNA repair' and 'immune system process') or it spreads over wider areas of the SOM (e.g. for 'apoptosis').

Overrepresentation analysis is not restricted to single tiles but it can also be applied to the over- and underexpression spots detected in the previous subsection. Accordingly, overrepresentation of selected gene sets can be linked with additional properties of the expression profiles such as overexpression by combining spot selection with overrepresentation analysis. Particularly, the genes associated with each spot are analyzed for overrepresentation of genes taken from the collection of 1454 gene sets downloaded from the GSEA-homepage according to the GO-categories molecular function, biological process and cellular component (see methods section). The hypergeometrical distribution then provides an ordered list of gene sets ranked with decreasing significance of overrepresentation for each of the spots.

**Figure 5:**
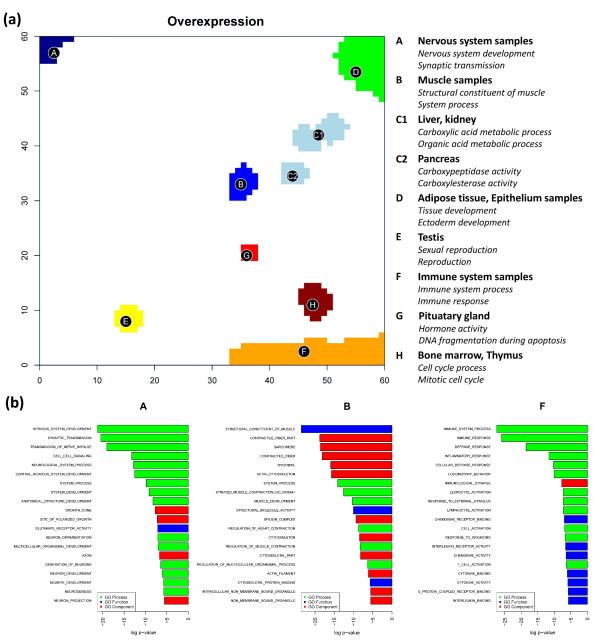
**The overexpression summary map shows nine spots which are strongly overexpressed in different tissues. **(part a) Overrepresentation of a collection of 1454 gene sets is estimated for each spot using the hypergeometrical distribution. The right legend assigns the two most significantly overrepresented gene sets to the respective spots. The top-twenty gene sets of the ranked list are shown in part b for three selected spots. The length of the bars scales with the logged overrepresentation p-value of the sets. The color assigns the category of the gene sets according to the GO terms 'molecular process' (green), 'molecular component' (red) and 'molecular process' (blue).

Figure 5a shows the overexpression summary map with nine spots of strongly overexpressed metagenes. The legend assigns the two leading gene sets in the list of each of the spots to get a first idea about the possible biological context of the genes in the spots. For example, spot A in the top left corner of the SOM is clearly related to molecular processes in nervous cells according to the leading gene sets (see also Table 1). The significance of overrepresentation of the top-twenty gene sets are visualized for three selected spots in Figure 5b using bar plots. Ten out of the top-twenty gene sets of spot A are related to nervous system (Figure 5b). Also other spots can be associated with distinct molecular functions such as immune system processes (spot F), sexual reproduction (spot E) or muscle contraction (spot B).

**Figure 6:**
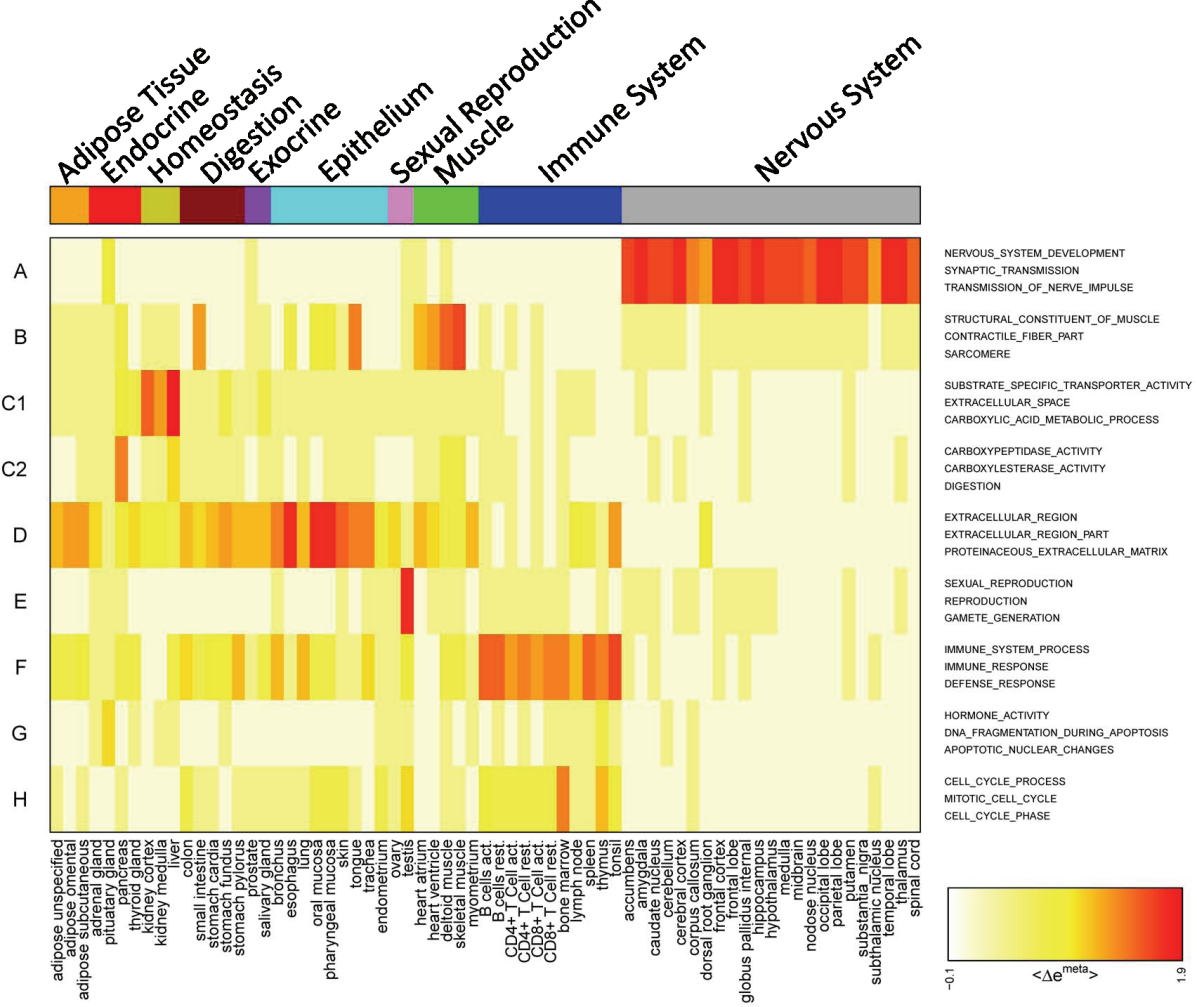
**Overexpression summary heatmap of selected global spots (A - H, see Figure 5 and Table 1) in all tissues studied**. The tissues are grouped into different categories in horizontal direction (see the color bar on top of the map; the colors are assigned to the categories in agreement with Figure 1). Each spot refers to one row. The top-three overrepresented gene sets are assigned in the right part of the map. The expression scale refers to the metagene of maximum expression in the respective spot.

The heatmap in Figure 6 visualizes the metagene expression in each of the spots in the series of tissues studied. It allows to link overexpression with overrepresentation in a tissue- and spot-specific way. It clearly reveals that nervous (see grey bar on top of the heatmap for assignment), muscle (green) and homeostasis (ocher) tissues are characterized by essentially only one overexpression spot (spot A, B and C1, respectively) with clearly assigned molecular function. Some of the tissue-specific spots are also overexpressed in other tissues. For example, the muscle-specific spot B shows overexpression also in tongue and small intestine which partly contain muscle tissues as discussed above.

It is noteworthy that the enriched areas in the overrepresentation maps of the gene sets 'nervous system development' and 'immune response' (see Figure 4) largely agree with the overexpression spots in the SOM images of nervous and immune system tissues, respectively. A non-negligible number of genes from these sets are however located in other regions of the map which are assigned to alternative molecular functions. For example, genes from the gene set 'immune response' accumulate in spot D assigned to tissue development. This spot is overexpressed in a larger number of tissues such as epithelium and adipose tissues which are not explicitly assigned to the category immune system tissues. Moreover, subgroups of genes from these gene sets are located in the central area of the map which accumulates virtually invariant and weakly expressed genes (compare with Figure 3). Possibly part of the genes in these sets are incorrectly specified and/or possess a more complex activation pattern 'beyond' the similarity metrics used to train the SOM. We suggest that combination of gene set overrepresentation analysis with SOM-expression profiling allows verification and further refinement of existing gene sets.

In summary, gene set overrepresentation analysis links selected gene sets and different regions of the SOM images with single-tile resolution. These regions, in turn, can be collected into over- or underexpression spots in different tissues. Overrepresentation analysis then provides lists of significantly overrepresented gene sets which characterize the respective spot in a functional context. Some of the spots can be assigned to specific molecular characteristics such as 'nervous processes', 'muscle contraction' and 'immune response'. Both, the single-tile SOM-wide and the multi-tile spot-wise overrepresentation analysis constitute a link between characteristic expression pattern and concepts of molecular function for the associated genes. These orthogonal views complement each other: The former one judges the homogeneity of a selected set with respect to different metagene expression profiles. The latter one assigns selected expression profiles to their tentative molecular function.

### 2.4. Filtering metagenes and single genes

The reduction of the size of the data set by removing genes that carry essentially no or low information is common practice to improve downstream analysis such as two-way hierarchical clustering of genes and samples. Such data reduction has been shown to result in dendrograms which more accurately reflect relationships between the samples with increasing stringency of the filter applied [24]. This improvement can be rationalized by the fact that random noise tends to disrupt similarity relations between genes and samples. On the other hand, also the opposite trend is possible: systematic errors in the data, e.g. due to batch effects, can cause artificial clustering if the bias affects subsets of genes in a coordinated fashion. Hence, a particular filter aims at improving data by removing either noisy, biased and/or weakly expressed genes. On the other hand, extreme filtering is dangerous because it may eliminate valuable information, for example, about genes of relatively low and thus noisy expression but with important biological impact. Hence, filtering is an optimization task with the requirement of removing virtually irrelevant data while preserving all information in the remaining part of the data which is important in the context of the particular issue studied. We will shortly call the latter property as the 'representativeness' of a filter and the former one as its 'noisiness', i.e. the mean noise-to-signal ratio of the data included. Optimization thus aims at maximizing representativeness while minimizing noisiness.

'Top-list selection' is probably the simplest method of filtering: One first defines a ranking criterion such as differential expression or variability (see below), then one ranks the data accordingly and finally selects a certain number of features on top of the list for further analysis. The length of the list can be cut by applying different criteria such as a fixed number of features or a significance threshold.

SOM analysis enables alternative filtering based on the metagenes as representative features characterizing the expression profiles of miniclusters of single genes. In other words, the metagene profiles itself can serve as a filtered and compressed extract of the original data. Our SOM-method assigns the expression profiles of the N = 22,277 input genes measured in 67 tissues to 3,600 metagene clusters. Each metagene cluster consequently contains G/M = <nk > = 6.2 real genes on the average. Hence, complexity of transcriptome characterization is reduced to about one sixth by utilizing the metagenes instead of the 'real' genes.

Moreover, the local G/M-ratio considerably varies between the different metagene clusters with minimum and maximum values of n_k _= 0 (empty metagenes) and n_k _= 308 (see Figure 3b). Thus each metagene can be representative for a very different number of real genes. In consequence, the importance of transcriptome information is effectively reweighted by using metagenes instead of real genes. For example, the metagene of highest population (n_k _= 308) clusters genes of virtually invariant expression profiles. These essentially not-informative features comprise 1.4% (308/22,277 × 100%) of all single genes but only 0.3% (1/3,600 × 100%) of all metagenes. Hence, their contribution is effectively down-scaled by nearly a factor of ~1/5 if one uses the metagenes instead of real genes. In other words, SOM clustering itself can be viewed as a sort of selective compression filter reducing the number of features considered by condensing larger numbers of similar single gene profiles into one metagene profile with a profile-specific compression factor, F_k_^compression ^= (n_k_⋅K/N)^-1 ^(K and N are the total numbers of metagenes and of single genes, respectively).

Metagene filtering is expected to outperform single gene filtering in terms of representativeness and noisiness because the reduced number of metagenes not only preserves the diversity of single gene profiles but it also amends the resolution of downstream analysis due to the reduced noise of the metagene profiles. With the objective of proving this expectation we compare two options for data filtering by applying top-list selection either to the metagenes or to the single 'real' genes. We used three types of filters to reduce the number of single genes and metagenes, namely fold change (FC)-expression, variance and significance (FDR-) filtering (see Additional file 1 and the methodical section). In the first case the full set of absolute FC-values of all genes (real genes and metagenes) under all conditions studied are ranked and a certain number of topmost features is considered for further analysis.

**Figure 7:**
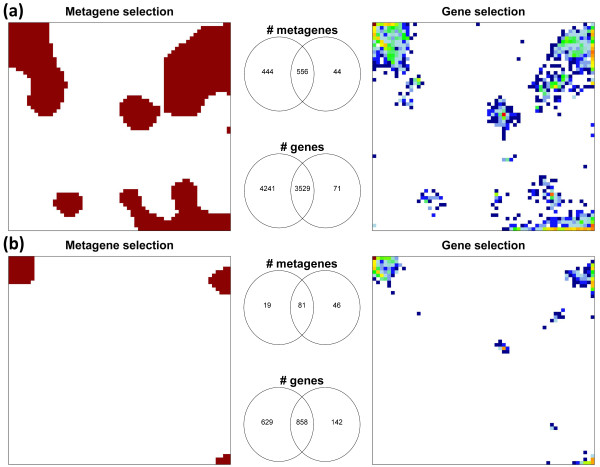
**Filtering genes or metagenes by differential expression: Different numbers of metagenes (left mosaics) and single genes (right mosaics) are selected using the FC-1000/FC-3600 (a) and FC-100/FC-1000 (b) filters to account for the data compression in the metagene clusters**. The brown areas in the left part show the selected metagenes and the colored tiles in the right part the fraction of single genes in the metagene miniclusters (maroon to blue codes high to low fractions). The Venn-diagrams illustrate the degree of overlap between the metagenes and single genes selected by both filters.

Note that lists of equal numbers of metagenes and of single genes are asymmetric owing to data compression in the metagene miniclusters. The different sample sizes selected by both options of filtering are given in detail in Additional file 1. Metagene lists integrate roughly a tenfold larger number of 'real' genes in our SOM settings. Figure 7 compares the areas in the SOM mosaic filtered by FC-lists of different lengths if applied either to metagenes or to single genes. The shorter metagene lists cover essentially the same regions of the SOM as the longer single gene lists with considerable overlap of the selected meta- and single genes. The large overlap demonstrates that the metagene filter is representative for the metagene-associated single genes which are also selected to a large fraction if one applies single gene filtering using a roughly ten-times longer list. For example, 3,529 out of the 3,600 single genes are shared by the FC-3600 single gene and the FC-1000 metagene lists ('FC-1000' denotes the 'fold change top-1000' criterion, see Figure 7a). However, 444 out of the top-1000 metagenes do not contain the genes from the single gene list which, on the other hand, contains 71 single genes in 44 metagenes not selected by the metagene list. Hence, the metagene filter covers a wider range of expression profiles than the single gene filter which selects only a few additional features. Figure 7b illustrates that different spot areas are progressively excluded from the list of filtered features with increasing stringency of the filter as expected.

In addition to FC-filtering we applied variance and significance filtering which select profiles of largest variance and of highest significance of differential expression, respectively. The former filter possesses similar properties as the FC-filters. In contrast, significance filters select more diverse collections of features which are spread over different areas in the respective mosaic representations (see Additional file 1). Below we apply FC-filtering in the more detailed analysis to judge the consequences of both filters for selected downstream characteristics.

### 2.5. Metagene- and single genes-based clustering analysis

In the next step we applied secondary standard analysis methods to the lists of filtered genes and metagenes to assess the particular effect of filtering. We performed one- and two-way hierarchical clustering and independent component analysis (ICA) using either the expression values of a list of real genes or of a list of metagenes of selected lengths. Hierarchical cluster analysis was applied because this method is often routinely run as a first step of data summary in microarray data analysis [25].

**Figure 8:**
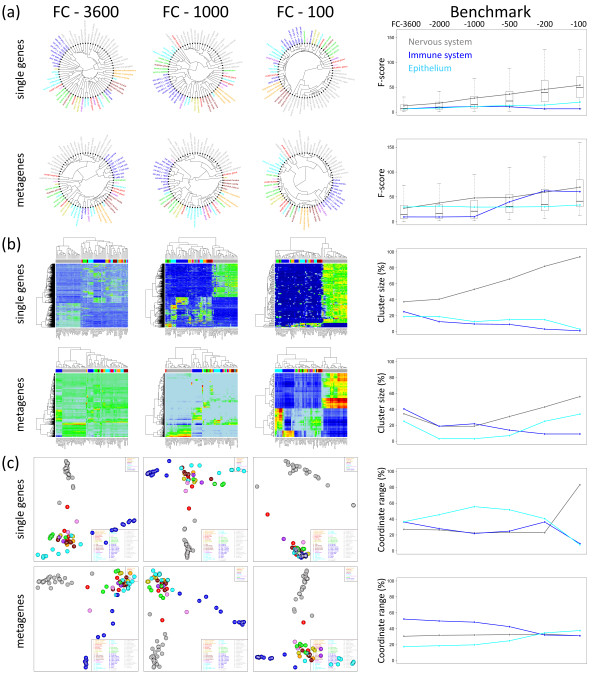
**The effect of filtering of single genes and metagenes on the results of one-way hierarchical clustering trees (part a), two-way hierarchical cluster heatmaps (part b) and independent component analysis (part c) of the 67 tissues studied**. The samples are color-coded according to the classification of tissues introduced in Figure 1. Top-list FC filters select the 3600, 1000 and 100 (from left to right) most strongly differentially expressed genes/metagenes in all samples. Note that the ICA-plots are invariant with respect to reversing the direction(s) of the coordinate axe(s) and thus to mirror and rotational symmetry operations. The right part shows different benchmark criteria for different lengths of the FC-lists ranging from FC-3600 to FC-100 (see top axis). The benchmark criteria were applied to nervous system, immune system and epithelium tissues (see text and Methods section).

One way hierarchical cluster trees obtained from single gene and metagene FC-lists of length 3600, 1000 and 100 reflect similar properties showing that clustering is relatively robust with respect to the chosen conditions (Figure 8a). Tissues from categories with homogenous SOM-pattern such as nervous (grey), adipose (orange) and immune system (blue) tissues (see also Figure 1) robustly cluster together at nearly all conditions studied. Note that the blue cluster of immune system tissues however partly decomposes if one uses the shortest single gene list (FC-100) owing to the loss of representativeness. On the other hand, the FC-100 metagene list of equal length still produces a compact blue cluster reflecting the improved representativeness of the same number of metagenes.

The blue immune system tissue cluster splits for both, the single gene and metagene filters in the opposite limit of low stringency using FC-3600 lists. These lists obviously become too long with worse noisiness characteristics. Note, that the FC-3600 metagene list considers all available metagenes whereas the FC-3600 single gene list is still limited to only 16% of all available single genes. Longer single gene lists reduce the quality of the observed cluster structure due to the progressive inclusion of noisy genes (data not shown). In summary, metagene lists are more representative and less noisy than single gene lists of equal length in downstream cluster analysis. On the other hand, also the length of metagene lists is optimal in the intermediate range (e.g., the FC-1000 list in our study): shorter and longer lists are suboptimal in terms of representativeness and noisiness, respectively.

The cluster trees generated on the basis of single gene and metagene lists reveal another interesting difference (compare the first and second rows in Figure 8a): The mean length of the outmost branches between the periphery of the circles and the first split point is considerably shorter for the metagene-based trees than for the single gene-based trees. This relation reverses for the innermost branches. This systematic difference indicates that metagene clusters are more compact than single gene clusters (an illustrative explanation for this difference is given in Additional file 1) owing to the decreased noisiness of the metagene data. In the right part of Figure 8a we compare the inter-to-intra cluster ratio of the Euclidian distances between the samples (F-score) for three tissue categories as a simple measure of the compactness of their clusters. The F-score of the metagenes systematically exceeds that of the single genes.

Figure 8b shows two-way hierarchical cluster heatmaps after FC-filtering of metagenes and single genes. This type of representation visualizes similarity relations between the samples in horizontal direction (see the color bars which assign the tissue categories) and between the filtered genes in vertical direction. One immediately observes that the contrast of the heatmaps increases from the left to the right because more stringent filters trivially accentuate larger differences between over- (red) and under (blue) expressed features. The loss of contrast for the longer FC-3600 and FC-1000 lists (compared with the FC-100 list) is stronger for the metagenes because data compression includes a larger fraction of features of small differential expression (green and light blue areas) than the respective single gene lists. On the other hand, the short FC-100 list of metagenes produces the heatmap of strongest contrast illustrating the favorable signal-to-noise characteristics of the filtered metagenes.

The heatmaps express detailed information about the amount of genes differentially expressed in the various tissues (cluster size, see the right part of Figure 8b). For example, the percentage of single genes which are overexpressed in the nervous tissues and underexpressed in the other tissue categories (see also the green/maroon area associated with the grey bar on top of the heatmaps) increases from values of less than 50% (FC-3600) to a dominating amount of more than 90% (FC-100) whereas the percentage of genes overexpressed in other tissue categories almost completely vanishes. Hence, the relative contribution of genes collected into clusters characterizing a selected tissue clearly depends on the length of the list. The use of metagenes instead of single genes effectively re-weights the contribution of tissue-specific genes. Particularly, the percentage of metagenes which are specific for nervous tissues is markedly smaller in the metagene list giving rise to a more balanced distribution of features.

### 2.6. Metagene- and single genes-based ICA analysis

Hierarchical clustering may identify groups of samples which share genes or metagenes of similar expression pattern. Hierarchical clustering however does not represent the multivariate structure of the data. Such aspects become highlighted by projecting the data to subspaces of lower dimension spanned by interesting modes such as the components of minimum mutual statistical dependence. ICA provides a visual plot in the space spanned by these independent components which are shown to point along the directions of maximum information content in the data or, equivalently, of non-normal distribution of the data [26]. We applied ICA to single and metagene lists to see which of the alternative data sets offers the better separation among the various tissue groups.

The ICA-plots of the two leading independent components shown in Figure 8c illustrate the degree of similarity between the samples as a function of the selected filters. All filters except one provide virtually three clusters, namely that of nervous (grey circles), immune system (blue) and the remaining tissues. The FC-100 single gene filter merges the latter two clusters due to its small representativeness with respect to non-nervous tissues (see also the respective heatmap in Figure 8b). Note also that the relative dimension of the three clusters in the ICA-plot and thus also their intrinsic resolution changes from filter to filter. These trends reflect the subtle interplay between the length of the list and its representativeness and/or noisiness which might overweight one tissue category and underweight another one. For example, the specifics of epithelium tissues (cyan circles) become relatively well resolved using the FC-100 metagene or, alternatively, the FC-1000 single gene lists. The respective heatmaps in Figure 8b confirm that this tissue category is well represented by a reasonable number of specifically over- and underexpressed genes/metagenes in these lists. The fraction of these genes however clearly decreases in the other filtering lists giving rise to the suboptimal resolution of the cluster of cyan circles in the ICA plots. The right part of Figure 8c compares the relative size of three clusters in terms of the fraction of the covered coordinate region. The metagene-based clusters are less dependent on the chosen length of the list and more balanced especially for short lists.

The ICA plots in Figure 8c reveal another interesting property inherent in the expression profiles: The points especially of nervous (grey) and immune systems (blue) but also of epithelium (light blue) tissues form chain-like clusters which point roughly along the coordinate axes. This pattern reflects the fact that the transcriptional activity of nervous tissues on one hand side and immune system and epithelium tissues on the other hand side are governed by different and mutually independent groups of genes. We will discuss this point below more in detail in the context of the SOM mosaics. In the context of the filter lists it should be noticed that this property of the data gets partly lost after most stringent single gene filtering (FC-100) whereas essentially all metagene lists well reflect the independence of the expression pattern of the different tissue categories.

In summary, ICA analysis illustrates the robustness and the discrimination power inherent in the metagene lists. The use of metagenes allows compressing the length of the list by about one order of magnitude without loss of information. The filtering conditions govern the resolution between different tissue categories in the ICA plot in a subtle way. Short and intermediate metagene lists provide best results in this respect. Notably, consideration of the full metagene information without filtering (FC-3600) provides still reasonably resolved clusters in the ICA-plot. In conclusion, metagenes are more robust with respect to the quality of secondary analysis than single gene lists owing to their better representativeness. Hence, the reduction of dimensionality provided by SOM analysis improves the performance of downstream hierarchical clustering and ICA analysis. The number of considered features can be reduced by about one order of magnitude without loss of information if one uses metagenes instead of real genes. Clustering and ICA characteristics obtained for the metagene and single gene lists after variance and FDR filtering virtually agree with the results of FC-filtering (see Additional file 4).

### 2.7. Metagene- and single gene-based correlation analysis

**Figure 9:**
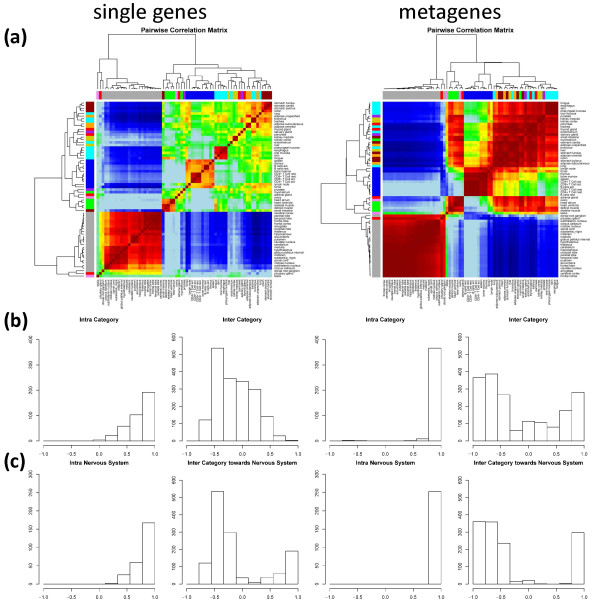
**Single gene (left panels) and metagene (right panels) correlation analysis of human tissues using the 1000/100 most strongly regulated genes/metagenes: (a) Pairwise Correlation Map (PCM); (b) Frequency distributions of correlation coefficients for all intra- and inter-tissue category pairings and (c) for pairings of intra-nervous tissue pairings and for pairings between nervous and all other tissues**. Note that the metagenes produce the stronger contrast of the PCM clusters due to the sharper and better resolved distributions.

In the next step we calculated pairwise correlation maps (PCM) illustrating Pearson correlation coefficients for all mutual combinations between the tissues. The PCM-heatmaps shown in Figure 9a are obtained using the FC-1000 (single genes, left part) and FC-100 (metagenes, right part) filters representing both roughly the same number of genes (see discussion above). The metagenes clearly provide PCM-patterns of higher contrast which becomes emergent as diagonal and off-diagonal dark red/maroon and blue clusters. They refer to tissue pairings with highly correlated and anti-correlated expression profiles, respectively. Both, the single gene and the metagene PCM reveal essentially four groups of tissues which consist mainly of nervous (see the grey bar at the margins), immune system (blue bar), muscle (green bar) tissues and also of a mix of diverse tissue categories.

The expression profiles of nervous tissues strongly anti-correlate with essentially all the other tissue categories, i.e. a gene overexpressed in nervous tissues usually becomes underexpressed in non-nervous tissues and vice versa. The original expression SOM always reflect this property showing one characteristic overexpression spot in the top left corner (see spot A in Figure 3 and Table 1) and otherwise a blue and light blue background due to underexpressed genes/metagenes (Figure 1). Muscle tissues show strong off-diagonal correlation with the group of diverse tissues but not with the immune system tissue group. This property can be mainly attributed to spot D in the right upper corner in the SOM of these tissues whereas the diagonal correlation component mainly originates from the muscle-specific spot B (see Figure 3 and Table 1). The cluster of immune system tissues along the diagonal of the PCM can be associated with spot F in their SOM. Hence, the diagonal and off-diagonal clusters in the metagene PCM can be related to different spots in the original expression SOM of the different tissue categories.

To get further insights into the origin of the contrast differences between the single gene and metagene PCM we calculated frequency distributions of the pairwise correlation coefficients either between tissues of one category or between tissues of different categories (Figure 9b). Intra-category correlation coefficients are expected to be close to unity because samples of the same categories show usually similar expression profiles. Indeed, these metagene correlation coefficients are close to unity as expected whereas the respective single gene correlations show a markedly broader distribution resulting in smaller correlation values on the average. Inter-category pairings of single genes show a broad distribution centered about zero with a strong component of anti-correlation near -0.5 revealing that single genes of different tissue types are either not or anti-correlated. The metagenes produce a more resolved trimodal distribution with strong components of correlated, anti-correlated and uncorrelated metagenes near 1.0, -0.7 and 0.0, respectively. The component peaks are clearly sharper and the whole distribution covers a wider range of correlation values. Hence, the metagenes obviously enable the better resolution of different subcomponents produced by different tissue types.

The PCMs reveal that anti-correlated metagene expression profiles are especially found between nervous tissues and the other tissues. We therefore calculated a second set of frequency distributions restricting the intra-tissue correlations to nervous tissues only and the inter-tissue correlations to that between nervous and all the other tissues (Figure 9c). The latter histograms reveal that the degree of anti-correlation is much stronger for the metagenes than for the single-genes again showing that metagenes more sharply express the correlation pattern of gene expression. Note that this anti-correlation is evident already in the textures of the original tissue SOM: Large blue areas in the SOM of nervous tissues reveal under-expression of the respective metagenes which become selectively overexpressed in the SOM of other, non-nervous tissues (Figure 1). The inter-nervous tissue correlation histogram also shows a strong correlation peak near unity which is caused by the metagenes commonly overexpressed in nervous tissues and pituatary gland (endocrine tissue, no. 5) as discussed above.

In summary, our extended dataset of human tissues confirms the results of Guo et al. [14] who found that SOM based metagenes well recapitulate gene expression profiles of the entire gene dataset despite dimension reduction and that the visual patterns capture the real similarity relationships among samples with a high fidelity. Moreover, one can improve the resolution power of popular standard analyses based on two-way hierarchical clustering or pairwise correlation heatmaps using metagenes instead of real genes. The SOM metagene pattern serves as an adequate data filter which appropriately selects representative features characterizing the expression properties of the system studied.

### 2.8. Selecting tissue specific metagenes: comparison of methods

**Figure 10:**
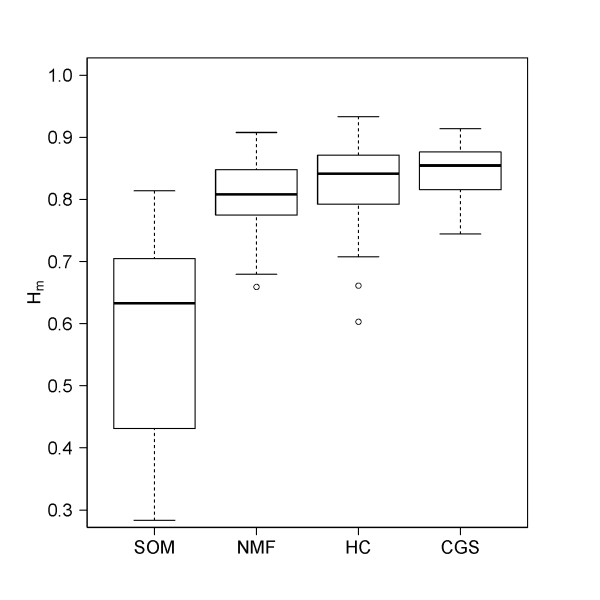
**Cluster specificity of different methods**. The specificity is measured in terms of the entropy (Eq. (7)): small values refer to tissues which are specifically characterized by only one cluster of high expression whereas large entropy values refer to tissues with more uniform expression of the metagene clusters. The boxplot illustrates the distribution of the entropy values for all tissues considered in each method.

One essential feature of the SOM approach discussed in the previous subsections is the reduction of dimensionality of the full data set from ten thousands of single gene expression profiles to a few thousand metagene profiles. In a second unsupervised reduction step, the dimensionality is further reduced to a handful of overexpression spots representing clusters of co-expressed metagenes which are highly expressed in, at minimum, one tissue. Particularly we demonstrated that the global expression landscape of human tissues is characterized by about nine- to - ten of such spots (see Figure 5). For comparison with these spot-clusters we applied selected alternative methods of dimension reduction: non-negative matrix factorization (NMF, see [27-29]), K-means hierarchical clustering (HC, see [25]) and correlated gene set clustering (CGS, [30,31]). These supervised clustering methods use different approaches: NMF virtually decomposes each of the expression profiles in original space into an additive set of 'metagene' profiles with non-negative expression amplitudes. HC is a heuristic iterative algorithm that tries to separate the original data into compact clusters using typically Euclidian distance metrics. CGS uses correlation metrics in combination with stringent significance testing to group the original data into groups of correlated single genes. NMF, HC and SOM were compared under different aspects in previous work (see [7,8,14,27,28] and references cited therein). Here we judge the ability of the methods to generate tissue-specific clusters using a simple entropy-measure [32]. We assume a number of ten clusters in each of the supervised clustering methods in correspondence with the SOM results. Figure 10 shows that SOM clustering outperforms the alternative methods in terms of specificity of the obtained spot clusters. In the supplementary material (Additional file 1) we show that SOM-clustering also outperforms the alternative methods in terms of representativeness and correlation contrast between nervous, immune systems and the remaining tissues similar to the results presented in the previous subsection.

Note that SOM, HC and CGS cluster genes together which show similar profiles in the series of samples using either distance or correlation metrics. Such groups of co-expressed genes can be interpreted in a common functional context based on the guilt-by-association heuristics [22] (see above). Instead, NMF decomposes the gene expression patterns as an additive combination of NMF-metagenes whereas SOM, HC and CGS use a decomposition that insists mutual exclusion of features [22,27]. The functional meaning of this polysemous decomposition of NMF in comparison with the exclusive guild-by-association decomposition is presently not clear and requires additional work.

### 2.9. Zoom-in step and similarity analysis

SOM expression profiles show very similar spot pattern for tissues of the same category in most cases. For example, the profiles of nervous and immune system tissues are commonly characterized by highly expressed metagenes in spot A located in the left upper corner and in spot F located in the right lower corner of the mosaic, respectively. Subtle tissue-specific characteristics are visible in the blue and green regions of under- and moderately expressed genes. These specific patterns of gene expression of selected subclasses of tissues were studied with increased resolution using a 'zoom-in' step which trains a new SOM based on the reduced set of tissues samples. The obtained expression images reveal a much more diverse spot pattern than the images obtained from the whole set of tissues discussed because SOM training adapts the expression profiles of the metagenes to a smaller bandwidth of expression values observed in the subensemble selected. Three examples for zoom-in analysis are presented in Additional file 1. We separately trained SOMs for the nervous tissues, immune system tissues and a collection of 31 diverse tissues including adipose, muscle and epithelial tissues.

**Figure 11:**
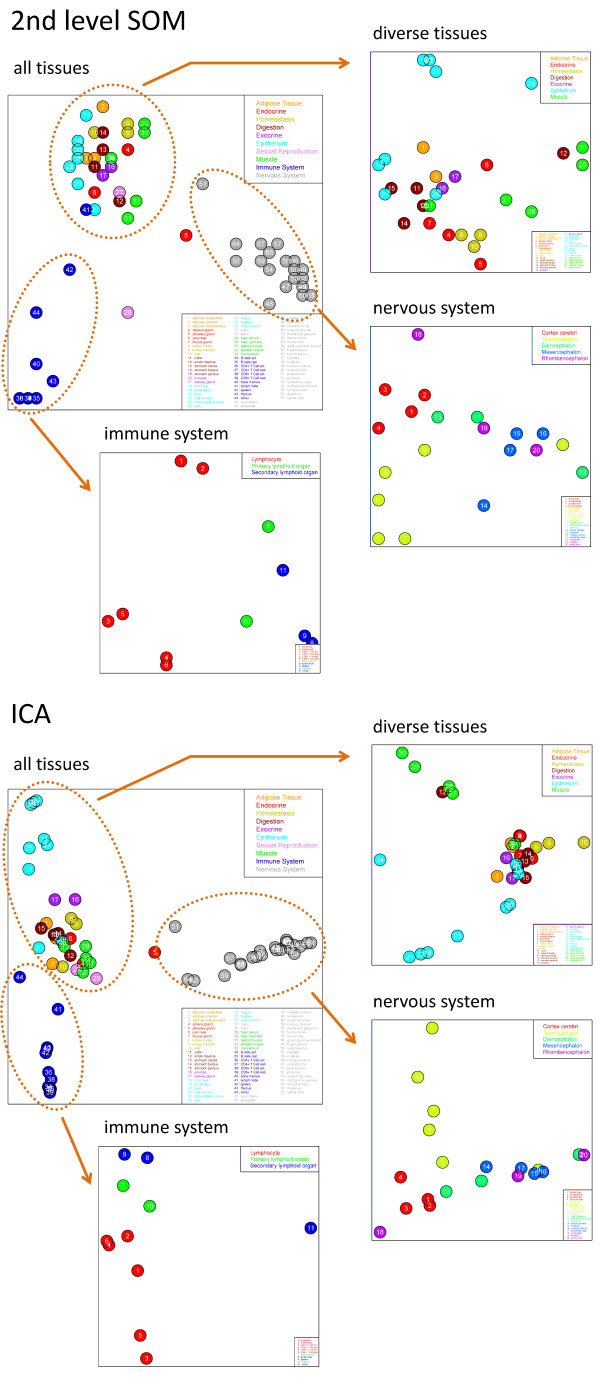
**Second level SOM and ICA plot of all 67 tissues and zoom-in views of the 31 diverse tissues, the 20 nervous tissues and the 11 immune system tissues (see arrows)**. Note that the nervous and immune tissue samples are re-colored in the zoom-in maps according to the sub-categorization of tissues applied (see Additional file 1 for details).

Guo et al. proposed a second-level SOM analysis step [14]. It maps all samples together into one two-dimensional mosaic pattern to visualize the degree of similarity between their metagene expression profiles. The second-level SOM algorithm uses the metagene expression of all samples considered as input. After training, each tile of the mosaic is characterized by the expression profile of one 'metasample' which serves as the condensation nucleus of the associated minicluster of real samples possessing similar SOM pattern. The mutual distances between the samples in the map are related to the degree of similarity of their SOM expression pattern. Figure 11 shows 2^nd ^level SOM and ICA maps of the 67 tissues studied. One distinguishes essentially the same three main clusters in both plots, namely that of nervous tissues (grey), immune system tissues (blue) and the remaining ones. The substructures of the three groups were further disentangled by applying the zoom-in step for each of the three tissue clusters as described above.

In general, ICA and 2^nd ^level SOM provide a similar view on the samples, however with subtle differences. For example, the ICA algorithm distributes the sample points continuously in the coordinate system spanned by the two leading principal components of maximum information content. The mutual separation between the points linearly scales with their distance in units of these components. In contrast, SOM machine learning uses non-linear scale to distribute the sample points in the discrete space defined by the mosaic grid of metasamples. It enables to display differences between the samples with improved resolution in regions of high sample density. In consequence, the individual tissues effectively spread over a larger area in the SOM mosaics than in the respective ICA.

As noticed above, most of the samples group into linear clusters which orient along one of the coordinate axes in the two dimensional ICA plots. The orthogonal orientation of most of these clusters indicates that each of them is characterized by genes which vary mutually independently. For example, nervous and immune systems tissues aggregate into such linear and perpendicularly-oriented clusters in the original ICA of all 67 tissues. Note that nervous and immune systems tissues are characterized by their specific spots A and F, respectively. Recall that these spots contain genes which indeed vary virtually independently.

Similar orthogonal clusters are found in the ICA plots after zoom-in of the nervous, immune system and diverse tissues. The obtained clusters reveal groups of tissues which are governed by independent sets of genes with enhanced resolution. For example one finds that telencephalon tissues (dark yellow circles in the ICA of nervous tissues) form one linear cluster which can be attributed to a category-specific spot in the zoom-in SOM images (see Additional file 1 for details). The linear clusters formed by muscle (green circles) and epithelium (cyan) tissues are oriented in perpendicular direction in the zoom-in ICA plot of the 'diverse tissues'. Both clusters are characterized by specific metagene spots in the SOM images after zoom-in. Tissues with mixed spot patterns due to different tissue components such as tongue are located at intermediate positions between that of the pure tissue components. We also generated three-dimensional ICA-plot to assess the third main independent component (see Additional file 1). These plots reveal that the characteristic pattern of orthogonal linear clusters of selected tissue categories can extend into the third dimension.

The second-level SOM and ICA plots similarly arrange the tissues samples. However, non-linear scaling of the SOM partly disturbs the linear arrangement of samples observed in the ICA plots. For example, the linear ICA-cluster of the nervous tissues (grey circles) transform into a slightly more compact cluster in the 2^nd ^level SOM. On the other hand, the 2^nd ^level SOM more in detail resolves small differences between the expression profiles (see, e.g. the zoom-in of the 31 diverse tissues). Hence, although very similar, 2^nd ^level SOM and ICA visualize partly complementary aspects of the data which can be studied more in detail using the spot-texture of the individual SOM of the samples studied. Tree-based similarity analysis provides an additional option to visualize the mutual relations between the samples (see Additional file 1).

## 3. Conclusion

The microarray expression data of 67 human tissues was used as an illustrative example to demonstrate the strengths of the SOM method in disentangling large sets of heterogeneous data. After suited preprocessing and training, the SOM method decomposes the original data into metagene expression profiles representing clusters of correlated single genes. Metagene expression values in the individual samples provide mosaic pictures visualizing tissue-specific over- and underexpression in terms of characteristic color-coded textures. They enable the direct comparison of the expression of individual samples in a simple and intuitive way.

Particularly, the tissue-specific patterns of gene expression were readily discernable in the obtained gallery of individual tissue maps. They reveal a series of about one handful stable over- and underexpression spots which selectively characterize different tissue categories such as nervous, immune system, muscle, exocrine, epithelial or adipose tissues. Single tissues of mixed characteristics such as tongue (composed of expression spots found in muscle or epithelial tissues) can be easily identified. Also anti-correlated expression spots are detected which, for example, are overexpressed in nervous tissues but underexpressed in the other tissues and vice versa.

To extract the functional context of spot and metagene related lists of single genes we applied overrepresentation analysis with respect to pre-defined gene sets of basically known functional impact. The mapping of overrepresentation of a selected gene set into the SOM mosaic provides a 'functional' map showing areas which are potentially relevant for this function. Tissue related spots typically contain enriched populations of function-related gene sets well corresponding to molecular processes in the respective tissues. This result strongly supports the 'guilt-by-association' principle that coexpressed genes are likely to be functionally associated. It, in turn, implies the ability to define either new gene sets using selected SOM spots or to verify and/or to amend existing ones.

The SOM method compresses the original set of high-dimensional data in two consecutive steps: Firstly, similar expression profiles of single genes are collected into metagene clusters, which reduces the number of relevant features nearly by one order of magnitude in our application. These metagene profiles can be understood as a sort of 'eigen-modes' characterizing the multitude of expression pattern inherent in the data. Secondly, the textures of the obtained SOM are decomposed into a few (typically less than one dozen) spots of similarly (over- or under-) expressed metagenes. This 'double compression' sequentially applies global (similar profiles) and local (over-/underexpression in part of the samples) criteria.

The use of metagene instead of single gene expression reduces the dimension of the data and leads to an increased discriminating power in downstream agglomerative analysis such as hierarchical clustering and independent component analysis owing to essentially two facts: Firstly, the set of metagenes better represents the diversity of expression pattern inherent in the data and secondly, it also possesses the better signal-to-noise characteristics as a comparable collection of single genes. Due to the better representativeness, metagene lists are less sensitive to downstream filtering than lists of single genes. Metagenes can be seen as a natural choice to detect context-dependent patterns of gene expression in complex data sets. SOM-spot clustering provides groups of genes of higher sample-specificity compared with selected alternative methods such as non-negative matrix factorization, hierarchical clustering and correlated gene set clustering.

Our example shows that SOM cartography transforms large and heterogeneous sets of expression data into an atlas of sample-specific texture maps which can be directly compared in terms of similarities and dissimilarities. This global view on the behavior of defined modules of correlated and differentially expressed genes is more intuitive than ranked lists of hundreds or thousands of individual genes. Importantly, the dimension reduction of the data does not entail the loss of primary information in contrast to simple filtering approaches which irretrievably removes part of the data. Instead, the reduction of dimension is attained by the re-weighting of primary information in the aggregation step. The whole set of single gene expression profiles remains virtually 'hidden' behind the metagenes. This primary information together with the respective gene annotations can be extracted in later steps of analysis to interpret the observed SOM textures using concepts of molecular biological function.

Finally, the software used in this publication is available as CRAN package 'oposSOM'.

## 4. Methods

### 4.1. Microarray data and preprocessing

Microarray raw intensity data (*.cel files, Affymetrix HG-U133 plus 2 array) of M = 67 tissues each measured in R_m _= 1, 2... (m = 1...M) replicates were downloaded from the Gene Expression Omnibus repository as the 'human body index - transcriptional profiling' - data set (http://www.ncbi.nlm.nih.gov/geo, GEO accession no. GSE7307; see Additional file 5 for the detailed list of samples used).

Raw probe intensities are calibrated and transformed into expression value using the hook method [33,34]. The expression values from all arrays are subsequently divided into present and absent ones [35] and normalized as described in the Additional file 1.

Logged expression values of each gene (e ≡ log_10 _E) were transformed into differential expression values relative to the mean expression of the particular gene in the experimental series of tissues considered,(1)

Eq. (1) thus defines differential expression in units of the logged fold change, logFC ≡ Δe.

### 4.2. SOM-mapping of gene expression profiles

In the next step, the preprocessed differential expression values of the series of tissue samples, Δe, are processed using the unsupervised machine learning method to train a self organizing map (SOM) representing information-rich diagrams. The SOM method applies a neural network algorithm to project high dimensional data onto a two-dimensional visualization space [1,36]. SOMs have a strong visualization capability by presenting each individual sample as an entity allowing its identification in a series of samples. Each SOM still keeps full high-resolution information about the co-expression pattern of the genes in the samples studied.

We applied a home-made R-program [37] which uses the CRAN package 'som' [38]. The SOM-algorithm assigns the expression profiles of the N input genes measured under M conditions to a number of K < N rectangular 'tiles' (so-called SOM nodes), each of which is characterized by one representative profile of metagene expression given by a vector of length M, Δe_k_^meta ^= (Δe_k,1_^meta^, Δe_k,2_^meta^,..., Δe_k,M_^meta^) (k = 1...K). It is trained such that the profiles of the metagenes capture the range of all individual expression pattern observed. Each individual expression profile of a 'real' gene is assigned to the metagene pattern of closest similarity using the minimum Euclidian distance as criterion. Each metagene thus serves as a sort of condensation nucleus for a minicluster of n_k _'real' genes with similar expression profiles, Δe_k,i _= (Δe_k,1,i_, Δe_k,2,i_,..., Δe_k,M,i_), with i = 1...n_k _and .

The metagenes are arranged in a two-dimensional grid with K = x⋅y tiles where x and y are the number of tiles per dimension. Most similar expression profiles of metagenes are located adjacent each to another. The correlation between metagene expression decreases with the mutual distance between the tiles on the mosaic. The degree of similarity between adjacent metagenes depends on the number of genes assigned to the respective metagenes being closer for larger populated metagenes and vice versa. For each condition m = 1...M a SOM mosaic pattern is constructed by color-coding the tiles k = 1...K according to its metagene expression, Δe_k,m_^meta^. This way one obtains a coherent mosaic pattern that is characteristic for each sample owing to the similarity of adjacent metagenes. Since the SOMs assign the same metagene to the same tile in all samples, they can be directly compared to each other allowing immediate identification of biologically interesting groups of genes.

Typically, the number of tiles to 'pixelate' the expression profiles is K = 10 × 10 - 100 × 100 = 10^2 ^- 10^4 ^with, on the average, n_k _= 5 - 100 genes per metagene. The obtained mosaic pattern is usually more homogeneous than typical gene clustering heatmaps containing typically about 10^2 ^clusters. This finer granularity of SOM-maps is associated with a fewer number of genes per unit (cluster/metagene) which in consequence gives rise to a more detailed expression pattern.

The number of tiles per SOM image and also the lattice-type (e.g., rectangular or hexagonal) potentially affects the obtained cluster structure and color texture of the images. In a preliminary study we found that the number of tissue-specific 'spots' converges for x = y > 50 and weakly depends on the chosen lattice type. Under these conditions the number of tiles exceeds the number of relevant expression modules roughly by two-orders of magnitude which allows their resolution with high granularity. The contrast of the SOM images can be adjusted using different color-scales to attenuate different aspects of the expression profiles with the aid of pattern recognition, feature selection and/or data filtering. In the supplementary information we compare three options of contrast variation with the focus on strong-to-moderate differential expression (log FC-scale), very strong overexpression (WAD-scale) or weak-to-moderate differential expression (log log FC-scale).

Details of these methodical studies are presented in Additional file 1 together with a schematic workflow of our SOM-pipeline used. The complete set of analysis results, as well as the current version of our R-program 'oposSOM' can be downloaded from http://som.izbi.uni-leipzig.de. The program is also available as CRAN package via http://cran.r-project.org/.

### 4.3. Supporting maps

We define the following supporting maps which provide additional information about the miniclusters defined by each metagene and the associated real genes:

(i) The metagene expression profiling map uses a coarse grained mosaic to provide an overview of the courses of the metagene profiles. For visualization purposes we use a coarse grained (e.g., 8 × 8) mosaic with considerably less tiles than the mosaic grid applied for the SOMs (60 × 60). The metagene profiles might be plotted together with the associated single gene profiles.

(ii) The population map plots the number of real genes per metagene in logarithmic scale, log n_k_.

(iii) The variance map illustrates the variability of the expression profile of each metagene in the samples studied,(2)

(iv) The integral over-/under-expression summary maps collect all over-/underexpression spots observed in the individual sample SOMs into one master map.

An extended set of supporting maps visualizing the covariance and the Euclidian distance between the genes and metagenes in each tile, the maxima and minima of the metagene profiles in absolute scale and the correlations between the metagenes are given as supporting information (Additional file 1). These maps illustrate the concerted changes of real genes in each of the metagene clusters and of the metagenes in the SOM images. It is shown that the Euclidian distance-based SOM algorithm implicitly clusters correlated expression profiles together in different regions of the SOM.

### 4.4. Gene set overrepresentation analysis

Gene set analysis requires the knowledge of predefined gene sets to study their enrichment in gene lists which are obtained from independent differential expression analysis (see [39] for a critical review and references cited therein). A large and diverse collection of such sets can be downloaded from the 'gene-set-enrichment-analysis'-website (http://www.broadinstitute.org/gsea). Particularly, we included in total 1454 gene sets in our analysis according to the GO terms 'biological process' (825 sets), 'molecular function' (396 sets) and 'cellular component' (233 sets). We use the term 'overrepresentation' to assign the probability to find members of a given set in a list compared with their random appearance independent of the values of their expression scores. We use the hypergeometric distribution to characterize overrepresentation in terms of a p-value which estimates the probability to find a stronger overlap between the list and the set by chance [40,41].

### 4.5. Grouping samples: Second level SOM cartography

We applied second-level SOM analysis as proposed by Guo et al. [14] to visualize the similarity relations between the individual SOM-metagene expression patterns. Second-level SOM analysis uses the K metagene expression profiles of the M samples as input. It then clusters the samples and not the genes as in first-level SOM analysis. Each tile of the second-level SOM mosaic characterizes the expression profile of a representative metasample defined by K metagene expression values. The M samples were presented using a mosaic grid of size K_2SOM _> M. Note that the number of metasamples usually exceeds the number of real samples whereas in first order SOM the number of metagenes is usually much smaller than the number of real genes. A considerable fraction of tiles of the second order SOM are consequently empty with no sample assigned.

### 4.6. Estimating similarities: Clustering-, tree- and independent component-analysis

One- and two-way hierarchical clustering [25] and independent component analysis [42] were applied in two versions using either the profiles of the SOM-metagenes (metagene analysis) or the profiles of individual 'real' genes (single gene analysis) using the R-packages 'stats' and 'fastICA' for clustering and ICA, respectively. Hierarchical clustering uses Euclidian distances between the genes/metagenes as similarity measure, whereas ICA is based on covariance. In addition to two-way hierarchical clustering heatmaps, we generate pairwise correlation maps (PCM) which visualize the Pearson correlation coefficients between the gene expression profiles (metagenes or 'real' genes) in all pairwise combinations of samples.

### 4.7. Filtering genes and metagenes

Optionally, the number of real genes and/or metagenes used in the analyses is reduced by applying three types of filters to exclude genes/metagenes of weak or of virtually invariant differential expression from downstream analysis: (i) FC-filtering: the genes/metagenes are ranked with decreasing absolute value of the fold change (FC) for each sample and a certain number (e.g., 100, 1000 and 3600) of the top-most features is selected; (ii) Variance filtering: the genes/metagenes are ranked with decreasing variance of their expression profiles and a certain number of top-most features is selected; (iii) FDR-filtering: only genes/metagenes with a local false discovery rate (FDR) smaller than a certain threshold (0.005, 0.01, 0.05) were selected. The local FDR estimates the probability of false positives in a list genes/metagenes. We used a shrinkage t-score statistics to assign p-values to each single gene the distribution of which then provides its FDR-values. The FDR of the metagenes is simply calculated as log-average of the single gene FDR of the respective metagene cluster. Details of the method will be published separately (Wirth and Binder; submitted).

### 4.8. Filtering benchmarks

The performance of metagene and single gene filters was compared using the following benchmarks (see also Figure 8):

*Hierarchical clustering*: The ratio of the inter-class and intra-class variance of the Euclidian distances between the respective expression data (F-score) was used to estimate the quality of the clusters.

*Two-way hierarchical clustering*: The percentage of genes/metagenes attributed to tissue-specific clusters for three tissue categories (nervous, immune systems and epithelium) was used to estimate the representativeness of the list.

*ICA*: The percentage of the variance of the independent components IC1 and IC2 of one tissue category, % = (varIC1+varIC2)_one_category_/(varIC1+varIC2)_three___categories_, was used to judge the relative size of the respective cluster.

### 4.9. Measuring cluster specificity with entropy and alternative clustering methods

The cluster-specificity estimates the degree to which the expression of a selected cluster differs from ubiquitous uniform expression of all clusters in a given tissue. It can be measured in terms of the entropy [32],(3)

where e_c,m _is the logged expression of the cluster which is calculated as mean value over the expression values of its members. The entropy is calculated for each tissue sample m = 1...M where the sum runs over all clusters c = 1...C. It has units of bits and ranges from zero for tissues with only one highly expressed cluster to 1 for tissues with uniformly expressed clusters.

For comparison with the SOM spot-clusters we applied selected alternative methods of dimension reduction: non-negative matrix factorization (NMF, see [27-29]), hierarchical clustering (HC, see [25]) and correlated gene set clustering (CGS, [30,31]). For NMF- and HC-clusterings we use the CRAN-package 'NMF' [43] and the basic package 'stats' [37], respectively. CGS-clusters were obtained using an in-house R-program [31].

## Authors' contributions

HW and HB: Conceived and designed this study, performed data analysis and wrote the manuscript. HW: Wrote the R-programs and performed the calculations. ML and MB contributed to finalize the study. All authors read and approved the final manuscript.

## Supplementary Material

Additional file 1 **The additional text describes methodical issues such as the calibration of microarray data and the adjustment of the size and topology of the SOM, additional supporting maps which illustrate the covariance and correlation structure of the metagene clusters, alternative options of contrast of the SOM images, the filtering of metagenes/single genes and the interpretation of cluster trees**. Further details of zooming-in of tissue subgroups are given together with the 3D-ICA plots of the tissues studied. Click here for file

Additional file 2 Whole set of 67 SOM expression profiles of human tissues Click here for file

Additional file 3 **Expression profiles of human tissues in alternative color scales**. Click here for file

Additional file 4 Agglomerative cluster analyses after single gene and metagene filtering using FDR and variance criteria Click here for file

Additional file 5 **Table of samples studied **The complete set of results of our SOM analysis of the human tissue dataset can be found on our website: http://som.izbi.uni-leipzig.de Click here for file
