# Supplementary material for: Expression cartography of human tissues using self organizing maps
Source: BMC Bioinformatics. 2011 Jul 27;12:306. doi: 10.1186/1471-2105-12-306 (PMC3161046; doi:10.1186/1471-2105-12-306)
Supplement: Additional file 1 — The additional text describes methodical issues such as the calibration of microarray data and the adjustment of the size and topology of the SOM, additional supporting maps which illustrate the covariance and correlation structure of the metagene clusters, alternative options of contrast of the SOM images, the filtering of metagenes/single genes and the interpretation of cluster trees. Further details of zooming-in of tissue subgroups are given together with the 3D-ICA plots of the tissues studied. [file 1471-2105-12-306-S1.PDF]

Supplementary text

Expression cartography of human tissues using self organizing maps

Henry Wirth[1,2]*, Markus Löffler[1,3,4], Martin von Bergen[2,5], Hans Binder[1,4]*

[1] Interdisciplinary Centre for Bioinformatics of Leipzig University, D-4107 Leipzig, Härtelstr. 16-18
[2] Helmholtz Centre for Environmental Research, Department of Proteomics, D-04318 Leipzig, Permoserstr. 15, Germany
[3] Institute for Medical Informatics, Statistics and Epidemiology, Universität Leipzig, D-4107 Leipzig, Härtelstr. 16-18
[4] Leipzig Interdisciplinary Research Cluster of Genetic Factors, Clinical Phenotypes and Environment (LIFE); Universität Leipzig, D-4103 Leipzig, Philipp-Rosenthalstr. 27, Germany
[5] Helmholtz Centre for Environmental Research, Department of Metabolomics, D-04318 Leipzig, Permoserstr. 15, Germany

* to whom correspondence should be addressed

Table of Contents

1. Preprocessing microarray intensities 2
2. SOM-analysis pipeline and availability of the program 4
3. Adjusting the size of the SOM 6
4. Adjusting contrast 10
5. Additional supporting maps 12
6. Differential expression summary maps 15
7. Correlation maps 17
8. Filtering metagenes and single genes 18
9. Clustering metagenes and single genes 23
10. Selecting metagenes using alternative methods: NMF, HC and correlated sets 24
11. Sample- and spot-related similarity trees 28
12. Zooming in: Expression map of nervous tissues 30
13. Zooming in: Expression map of immune system tissues 35
14. Zooming in: Expression map of 31 diverse tissues 40
15. 2nd level SOM and 3D ICA maps 44
16. References 46

1. Preprocessing microarray intensities

We consider a microarray data set consisting of the expression levels of N genes in M different sample categories such as different tissues, each measured in R_m (m=1…M) replicates. For gene expression studies the number of genes N is typically in the ten thousands, the number M of experimental conditions is typically in the tens to a few hundreds, and the number of replicates between one and ten. The used GeneChip microarrays provide typically eleven raw probe intensities per gene constituting one probe set. Raw probe intensity values of each of the M x R_m chips studied are calibrated and summarized into one expression value E per probe set using the hook method [27, 28]. The expression values of all arrays are subsequently quantile-normalized [29] (see Figure S 1 for illustration).

The obtained distribution of expression values shows typically a bimodal shape: It's left peak at smaller expression values and its right peak values were attributed to non-specific and specific hybridization, respectively [30]. The peak due to non-specific hybridization is non-informative with respect to the target genes which are therefore called 'absent' because their expression is smaller than the detection threshold of the method. The non-specific peak consequently characterizes the 'chemical' background of the measurement.

The distribution of expression data of each experimental series is then processed as follows: Firstly, the origin of the log-expression axis (log E=0) was positioned to agree with the peak position of the non-specific peak of the distribution. Secondly, both peaks are decomposed as described previously [30] assuming mirror symmetry of the left and right flanks of the non-specific peak (Figure S 1b). Thirdly, we make use of the decomposed distributions to estimate the probability that the specific expression of a selected gene is detected. This 'present-call'-parameter is set to pc=0 and pc=1 for genes with expression values outside the region of overlap of both peaks (see Figure S 1c). In the range of overlap, the present call is calculated as the fraction of the local density of the specific signal contributing to the total signal distribution. The resulting value of pc roughly linearly scales between zero and one with increasing expression in this range (Figure S 1c). Fourth, the log-expression of each gene is scaled with its present call, i.e., e= pc(e')*e' where lower case e' define the logged expression values, e'= log E. The used transformation thus considerably narrows the non-specific peak at position e'=0 of the expression axis while leaving the specific signal virtually unaffected. As a consequence, the variability of the signals of absent called and thus of non-informative probes is markedly reduced (Figure S 1c). This transformation enables to conserve the full set of available genes in the data set used for SOM analysis in contrast to data filtering which removes presumably uninformative probes from the data set prior to downstream analysis.

Expression values of replicates of the same tissue were log-averaged and finally, the logged expression values of each gene were transformed into differential expression values relative to the mean expression of each particular gene in the experimental series of tissues considered (Figure S 1d),

$$\Delta e = e - \langle e \rangle_{all\_tissues} \qquad (1)$$

Eq. (1) thus defines differential expression in units of the logged fold change, logFC≡ Δe.

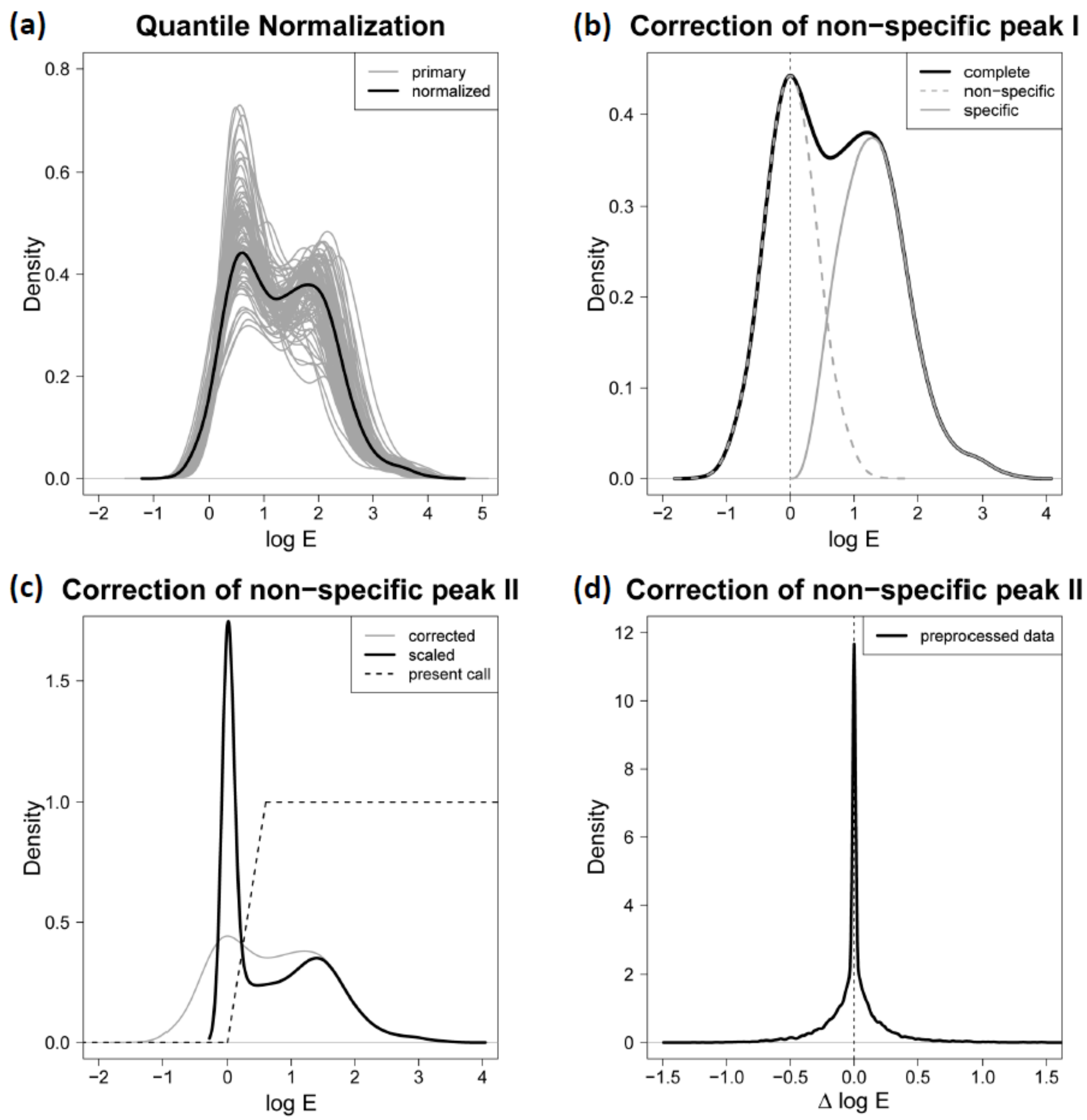

Figure S 1: Normalization and adjustment of expression values: The different distributions of hook-calibrated expression values of the samples studied merge into one representative mean distribution after quantile normalization (panel a). Its double peaked shape is decomposed into two single peaked distributions due to non-specific and specific hybridizations at small and larger expression values, respectively (b). The fraction of the specific signal contributing to the total signal density (dashed curve) is used as weighting coefficient of the expression values, e= pc(e')*e', which reshapes the total signal density (c). Finally, the expression values are normalized with respect to the logged mean expression of each gene (d). The large central peak refers to invariant genes under all conditions studied.

2. SOM-analysis pipeline and availability of the program

Figure S 2 illustrates the SOM-pipeline used in this study: Raw microarray probe intensity data referring to an experimental series of different conditions was preprocessed including calibration, normalization and adjustment. The obtained expression values are then used in the SOM-algorithm. It translates the high-dimensional expression data into a two-dimensional grid of expression profiles. Each tile represents a cluster of individual genes (thin lines in the graphs, numbers of genes are given for each cluster) characterized by the expression profile of a representative metagene (thick lines). The expression profiles of the metagenes are then transformed into one mosaic image per condition which are shown in the row „expression profiles“ in Figure S 2. The tiles in these maps are color-coded to represent overexpression or underexpression of each metagene in the respective sample to map the underlying gene expression pattern. The parallel evaluation of multiple samples allows linking their overall profile pattern. For example, the metagene of the tile in the top left corner of the mosaic is underexpressed in sample no. 1 and overexpressed in sample no. 2 as indicated by the red and blue circles and the respective color-code in the respective pictures. Summary maps characterize different aspects of the individual SOM such as the population of metagenes or the summary of all overexpression peaks. Metagene expression can further be used for statistical and functional analysis as will be described in a separate publication (Wirth, Binder; submitted). In the last step, summary reports for each sample are generated providing lists of differentially expressed genes, enriched gene sets, error statistics and further information. The complete analysis results can be downloaded from http://som.izbi.uni-leipzig.de. Also the current version of our R-program can be downloaded from this website and as CRAN-package ‘oposSOM’ from http://cran.r-project.org/.

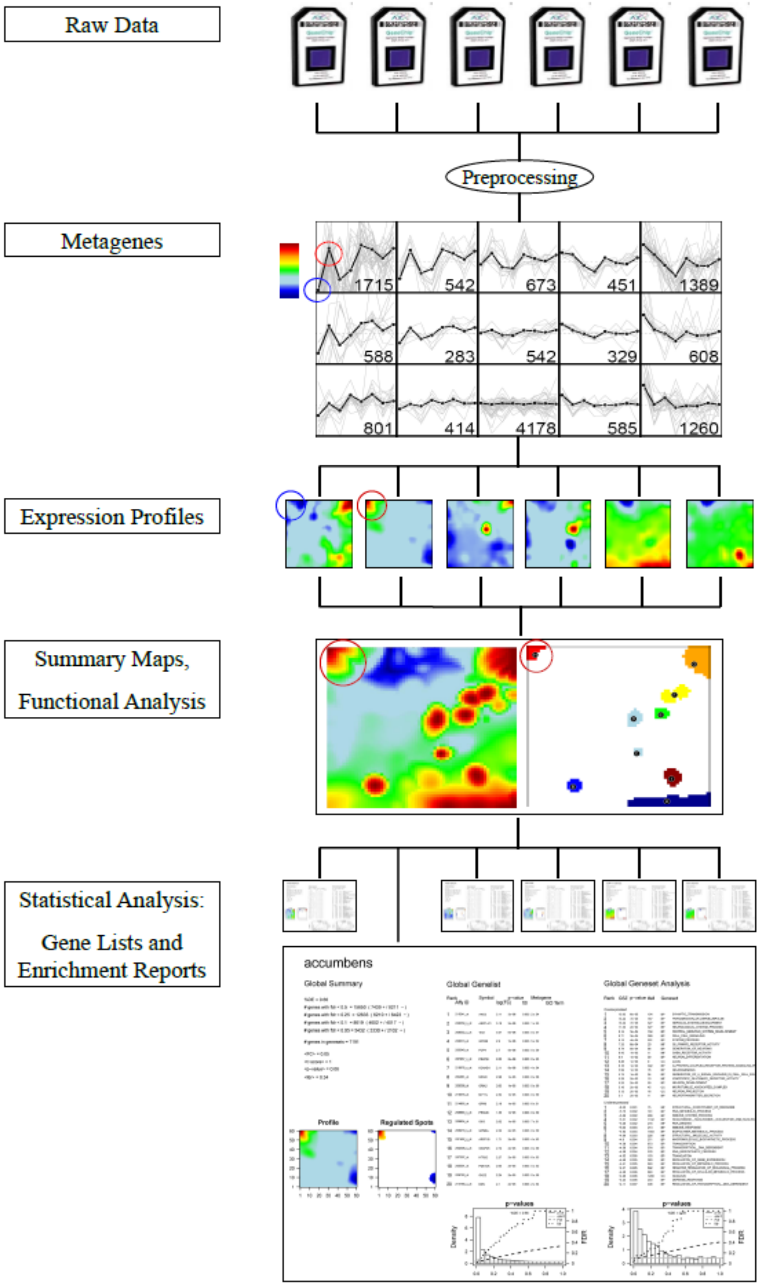

Figure S 2: Expression profiling using self organizing maps (SOMs).

3. Adjusting the size of the SOM

SOM machine learning is an unsupervised neural network algorithm that potentially clusters the data into biologically meaningful groups, e.g., of genes which are overexpressed in the same tissue. The number of tiles of the SOM-image ('SOM' size) is chosen by the researcher in a supervised fashion. Each tile of the map represents a separate mini-cluster of co-regulated single genes. Actually several neighboring tiles may cluster into one spot together because they collect genes of similar expression profiles in the experimental treatments studied. In our example each spot can be assigned to groups of co-expressed genes overexpressed in at minimum one tissue. The number of such spots is an intrinsic property of the expression pattern studied, which should not depend on the clustering algorithm and the particular parameter settings. Consequently, the SOM algorithm must be configured such that it produces a stable and consistent spot pattern.

The SOM can be configured by the numbers of tiles used in the image $K=x^2$ (x is the number of tiles in one direction assuming a quadratic mosaic), different topologies (e.g., with rectangular or hexagonal lattices), and different neighborhood functions describing the range and strength of interactions between the nodes during the training process. We studied the sensitivity of the resulting pattern of overexpression spots as a function of the SOM-size $x=\sqrt{K}$ for rectangular (Figure S 3) and hexagonal lattices (Figure S 4) using a Gaussian neighborhood decaying according to a normal distribution around the central node [1], and for a rectangular lattice using a 'bubble' neighborhood which equally affects adjacent nodes (Figure S 5).

The size of the SOM determines the resolution of the resulting mosaic image. For small SOM sizes each tile (or metagene) will contain a large number of single genes profiles whereas large sizes enable the distribution of the genes over a larger number of metagene clusters which more specifically can express the properties of the single gene profiles. Our analysis shows that with increasing size of the SOM the number of observed spot-clusters increases from two to at minimum nine (see panel a of the figures). Each of these spots can be associated with genes overexpressed in a particular group of tissues. The number of clusters and their assignment converges for SOM-sizes x>30 (hexagonal lattice) and x>50 (rectangular lattice) to a stable and virtually identical spot pattern. At these conditions the number of tiles (K=900 – 2,500) exceeds the number of spot clusters (9 -10) at minimum by two orders of magnitude. The hexagonal topology of the grid is a bit more homogeneous with respect to the directions on the SOM plane than the alternative rectangular topology. However the results are very similar with both choices.

The correlation between the single gene profiles within each spot (intra-spot correlation) initially increases with increasing size of the SOM but then levels off to a constant value for x>20 (see part c of the figures). This asymptotic behavior indicates that larger SOM sizes essentially do not improve the obtained spot-clusters. Hence, the obtained clusters indeed reflect intrinsic properties of the overall expression pattern. Please note that this limit x>20 is slightly smaller than the limit discussed in the previous paragraph (x>30-50) because it judges the mean clustering behavior whereas the latter tree-criterion refers to the appearance of the full set of expected spots. Contrarily, the correlation between the single gene profiles within each tile of the SOM (intra-tile correlation) permanently increases over the whole range of SOM-sizes considered without a limiting asymptotic level. Note that the distribution of the single genes over an increasing number of tiles allows the finer adjustment of mutual similarities of their expression profiles which will increase the respective intra-tile correlations. The largest SOM-size studied refers to a mean gene-to-metagene ratio of G/M=6. In other words, one finds six single genes in each of the tiles on the average (see also the population map below and in the main paper).

The 'bubble' neighborhood conditions tend to 'over-compartmentalize' the SOM images where some of the tissue-specific spots become fragmented into two or more correlated subspots.

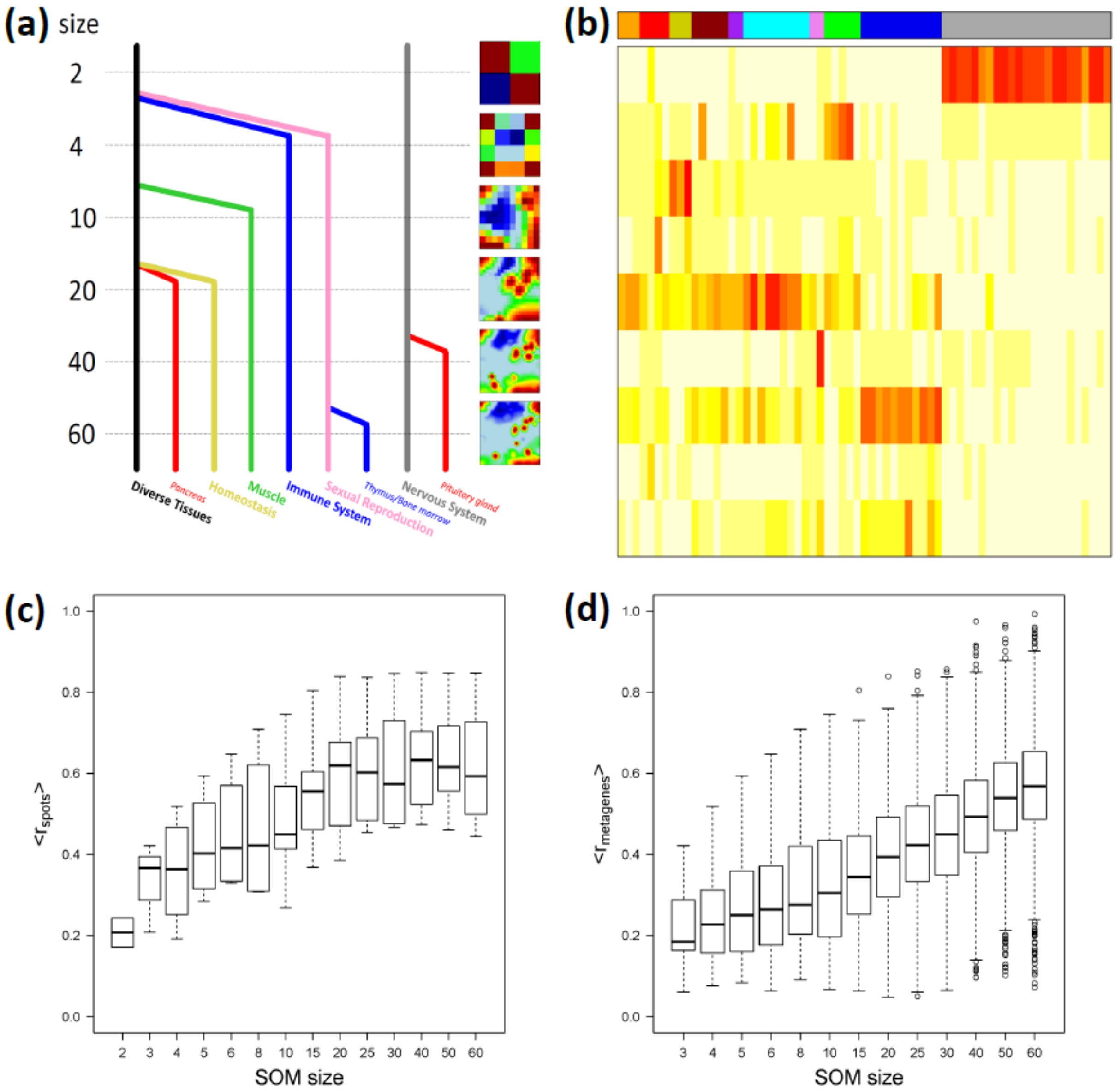

Figure S 3: Performance of SOM with two-dimensional rectangular mosaic topology and Gaussian neighborhood as a function of the number of tiles per axis (SOM size). a) The spot tree shows the tissue categories or individual tissues which can be identified by characteristic spots in the SOM-images. The respective overexpression summary maps are shown in the right part of the panel. b) The spot overexpression heatmap visualizes in which tissues (columns, the tissue categories are colour-coded) the spots are overexpressed (60x60 SOM). c) Box plots of the intra-spot Pearson correlation coefficient of the expression profiles of the single genes (panel c) and of the respective intra-tile correlation coefficient as a function of the SOM-size.

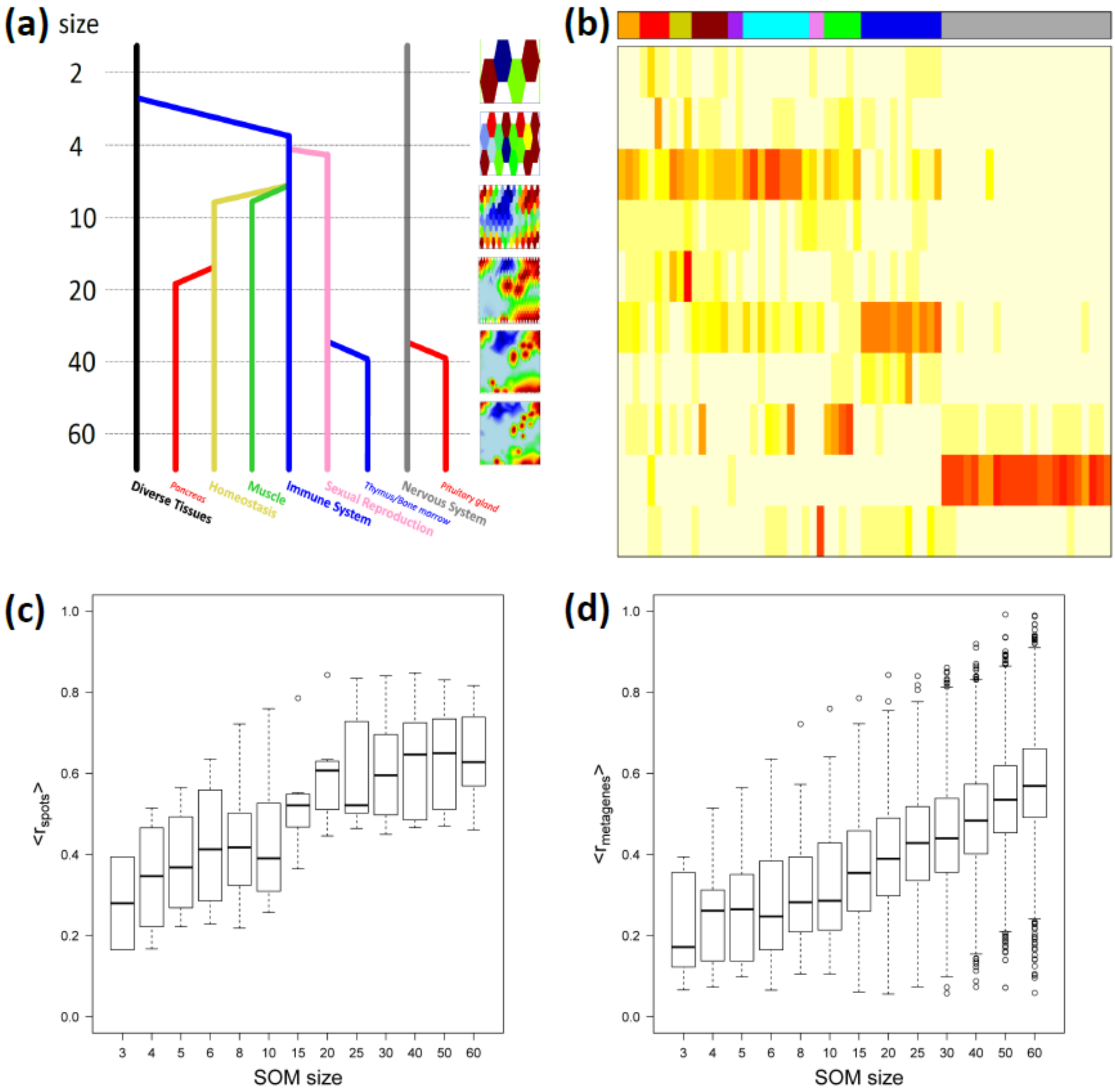

Figure S 4: Performance of SOM with two-dimensional hexagonal mosaic topology and Gaussian neighborhood as a function of the number of tiles per axis (SOM size). See legend of Figure S 3 for further details.

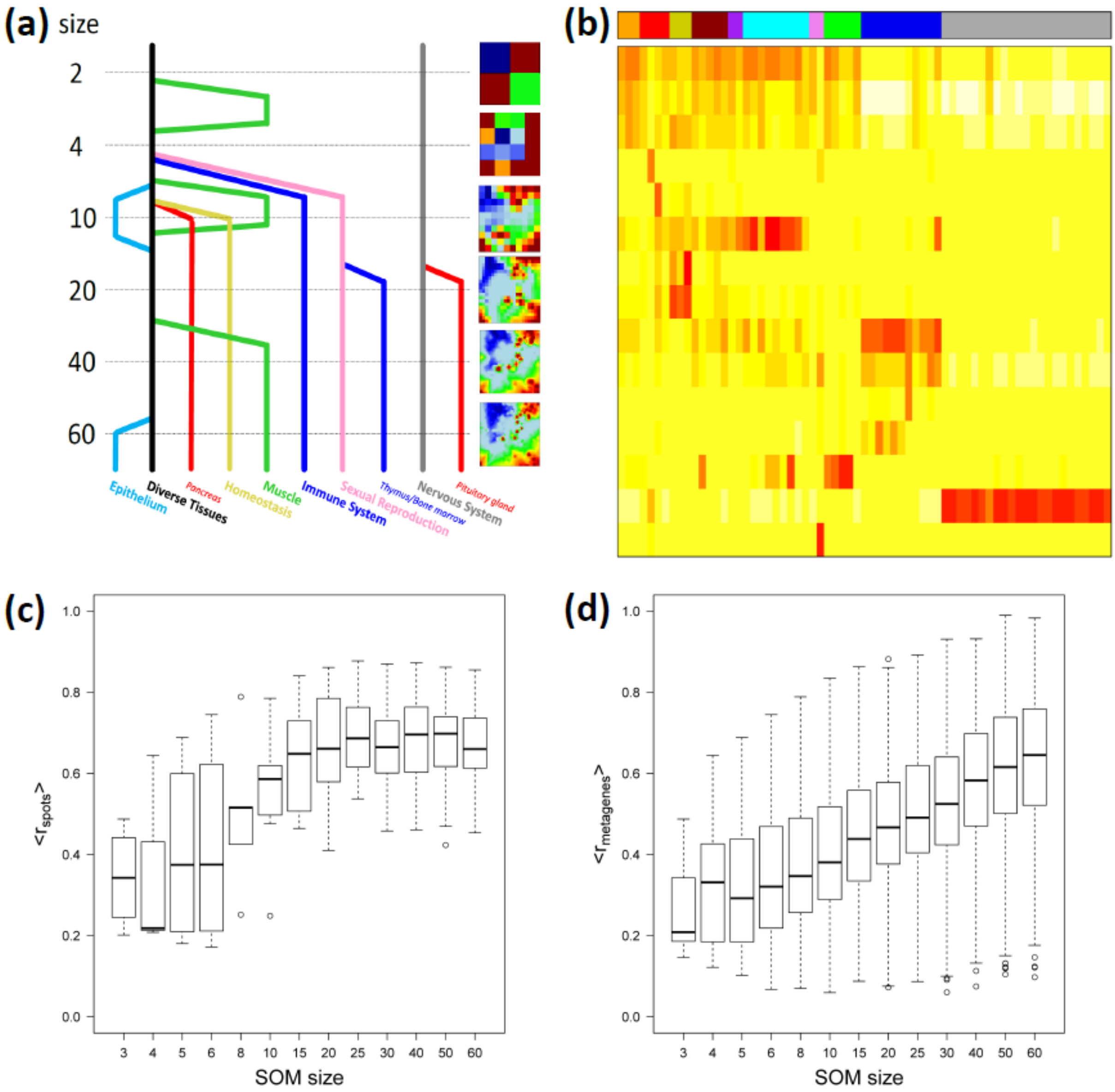

Figure S 5: Performance of SOM with two-dimensional rectangular mosaic topology and 'bubble' neighborhood as a function of the number of tiles per axis (SOM size). See legend of Figure S 3.

4. Adjusting contrast

Our standard SOM method scales the differential expression in units of the logged fold change of the metagenes, log FC = $\Delta e_{k,m}^{meta}$. The observed spots thus mark regions of over- and under-expression in the respective metagene profiles in logarithmic scale (Figure S 6a). Alternative scales, such as the double logarithmic log log FC ~ log $\Delta e_{k,m}^{meta}$, and the so-called weighted average difference score (WAD), are applied to vary the contrast of the texture of the SOM mosaics in order to highlight different aspects of the expression profiles.

The WAD-score is calculated for each tile k and sample m according to

$$\mathrm{WAD}_{k,m} = w_{k,m} \cdot \Delta e_{k,m}^{meta} \quad \text{with} \quad w_{k,m} = \frac{\Delta e_{k,m}^{meta} - \min\left(\Delta e_{k,m}^{meta}\right)}{\max\left(\Delta e_{k,m}^{meta}\right) - \min\left(\Delta e_{k,m}^{meta}\right)} \quad . \tag{2}$$

The WAD score is a fold change (FC)-based score which 'amplifies' large expression values [2]. The main idea of the WAD method is based on the observation that potential marker genes tend to have high expression levels. Moreover, it intuitively considers the fact that the experimental error of expression values typically inflates at small expression levels in logarithmic scale [3]. Hence, the basic assumption for the WAD-approach to the gene ranking problem is that 'strong signals are better signals'. It is suited especially for small sample sizes and it partly outperforms popular standard methods for determining differentially expressed genes when sensitivity and specificity are considered simultaneously [2, 4]. The WAD-score approximately changes as a quadratic function of differential expression, WAD ~ Δe^2 (see Eq. (2)), highlighting peaks due to overexpression more sharply with higher contrast as shown by the density distribution of the respective scores (Figure S 6, part b).

As third option, the original FC color-code was rescaled into double-logarithmic units giving rise to a wider distribution in the positive and negative expression ranges, which strongly enhances the discrimination between up- and downregulated metagenes (Figure S 6, part c). The log log FC-scale thus expresses structured blue and red areas of characteristic shape which clearly identifies the borderline between the regions of over- and underexpression. These details are not or only hardly detected in the log FC- and WAD-scales. In contrast, FC- and even more the WAD-scales express a spot-like pattern which is characteristic for most of the samples.

The used options of contrast variation enable to accentuate different ranges of metagene differential expression with the aid of pattern recognition, feature selection and/or data filtering with the focus on strong-to-moderate differential expression (log FC), very strong overexpression (WAD) or weak-to-moderate differential expression (log log FC). For example, the three adipose tissues show very similar images with essentially the same overexpression spot in the log FC and WAD scales whereas the log log FC map reveals subtle differences between the underexpressed blue regions of 'adipose omental' tissue and the other types of adipose tissues.

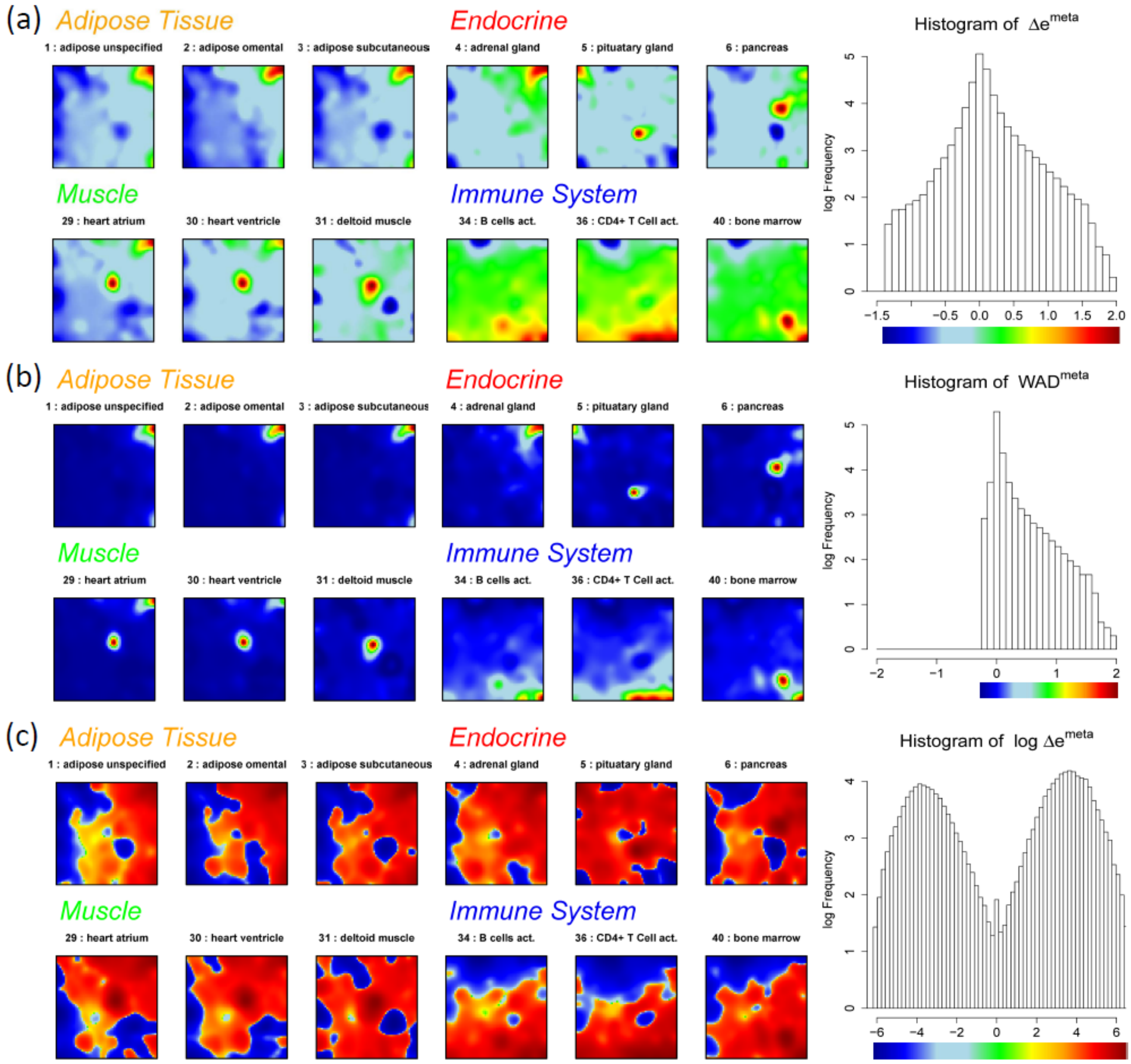

Figure S 6: Contrast variation of the SOMs using different expression scores in selected tissues: Fold change of metagene expression relative to the mean expression in all samples studied in logarithmic (a, Eq. (1)) and double-logarithmic scale (c) and using the WAD-score (b, Eq. (2)). The right part of the figure shows the frequency distribution of the scores in logarithmic scale. The whole set of SOMs for all tissues studied is given as additional file).

5. Additional supporting maps

we define the following supporting maps in addition to the supporting maps discussed in the main paper (expression profiling map, population map, variance map, integral over-/under-expression summary maps) to extract complementary information about the metagene and single gene profiles visualized in the SOM-images:

(i) The covariance map visualizes the degree of concordance between the expression profiles of the real genes and that of the metagene in each metagene minicluster in terms of the cross correlation coefficient,

$$r_k = \frac{1}{n_k}\sum_{i=1}^{n_k} \mathrm{cov}_{k,i} / \sqrt{\mathrm{var}_k^{meta} \cdot \mathrm{var}_{k,i}}$$

$$\text{with} \quad \mathrm{cov}_{k,i} = \frac{1}{M-1}\sum_{m=1}^{M}\left(\Delta e_{k,m}^{meta} \cdot \Delta e_{k,m,i}\right) \quad \text{and} \quad \mathrm{var}_{k,i} = \frac{1}{M-1}\sum_{m=1}^{M}\left(\Delta e_{k,m,i}\right)^2 \quad . \qquad (3)$$

(ii) The deviation map visualizes the degree of concordance between the expression profiles of the real genes in each metagene cluster using the quadratic mean of the Euclidian distances between each metagene and the respective single genes

$$d_k = \sqrt{\frac{1}{n_k}\sum_{i=1}^{n_k} d_{k,i}^2} \quad \text{with} \quad d_{k,i}^2 = \frac{1}{M-1}\sum_{m=1}^{M}\left(\Delta e_{k,m,i} - \Delta e_{k,m}^{meta}\right)^2 \quad . \qquad (4)$$

These supporting maps use the same resolution of the two-dimensional mosaic grid as the SOM representation and appropriate color-scales for direct comparison.
Elementary algebra links the Euclidian distance, the metagene variance and the covariance (see Eqs. (3)-(4)), $d_k^{\,2} = \mathrm{var}_k^{meta} + \frac{1}{n_k}\sum_{i=1}^{n_k}\left(\mathrm{var}_{k,i} - 2\cdot \mathrm{cov}_{k,i}\right)$, where var_k^{meta} is the variance of the metagene profiles defined in the main paper. Under the assumption of equal variances of the single genes and of the metagene in each tile ($\mathrm{var}_{k,i} \approx \mathrm{var}_k^{meta}$) one gets

$$r_k = 1 - \frac{d_k^{\,2}}{2\,\mathrm{var}_k^{meta}} \quad . \qquad (5)$$

Eq. (5) links the Euclidian distance used to train the SOM and to partition the single genes among the metagene miniclusters with the correlation coefficient. It shows that correlation coefficients near unity are obtained for close similarity in terms of the former measure ($d_k \rightarrow 0$) and/or if the metagene variance largely exceeds the squared Euclidian distance, $d_k^{\,2} \ll \mathrm{var}_k^{meta}$. Note that the correlation coefficient vanishes for $d_k^{\,2} \approx 2\,\mathrm{var}_k^{meta}$.
The population, variance, covariance and deviation maps shown in Figure S 7 provide information about special properties of the individual tissue SOMs using the same number of tiles. The population map reveals that the real genes inhomogeneously distribute among the tiles of the mosaic (Figure S 7a, see also the main paper). The tile of maximum population (n_k=308, see the dark brown tile slightly left from the centre of the map in Figure S 7a) refers to genes with virtually invariant, mostly absent specific expression in all tissues studied. These genes form the strong peak in the distribution of differential expression shown in Figure S 1c and d. These invariant genes give rise to the dark blue spot in the central area of the variance map (Figure S 7b). The covariance and concordance maps show a similar but more noisy pattern as the variance map due to the fact that they explicitly process single gene profiles (Figure S 7c and d, respectively).
The three measures variance, covariance and Euclidian distance plotted in different maps are linked properties (see Eq. (5)). Accordingly, the three maps confirm the concerted changes of real genes together with that of the associated metagenes in each tile (compare Figure S 7b and c). The deviation map more accentuates metagenes of low variance (blue areas in Figure S 7d).

Recall that the SOM algorithm uses the Euclidian distance between single and metagene profiles as similarity criterion to partition the single genes over the tiles of the mosaic. Close similarity in distance scale transforms into correlation coefficients near unity in the areas of relatively large metagene variance as predicted by Eq. (5) (see read areas in Figure S 7b and c). Contrarily, areas of relatively weak correlations largely agree with the regions of low metagene variance (see blue and green areas in Figure S 7b and c) which, in turn, lack marked over- and overexpression spots.

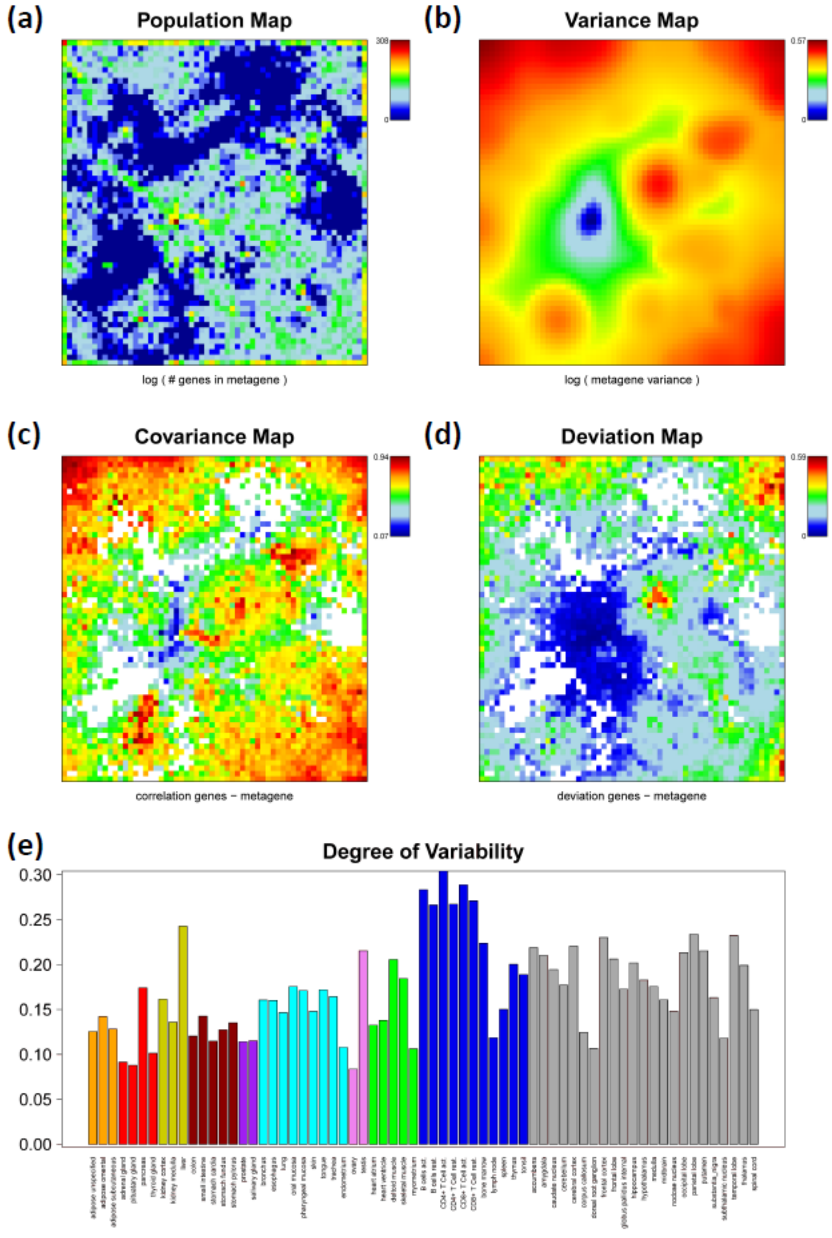

Figure S 7: Supporting maps characterizing the expression profiles of metagenes: Population (panel a), variability (b), covariance (c, Eq. (3)) and deviation (d, Eq. (4)) maps. Panel (e) shows the variability of metagene expression values over the tissue-samples according to Eq.(6). The different colors indicate the different tissue categories.

Alternatively one can apply correlation-based metrics such as the correlation coefficient between the metagenes and single genes. Correlation metrics however fail to differentiate between relatively invariant expression profiles of similar shape (small d_k^2 and var^{meta}_k see (5)) and very noisy profiles (large d_k^2 and var^{meta}_k). It has been shown that distance and correlation metrics provide different results in clustering gene expression data mainly because the correlation coefficient is prone to small measurement errors in uniform profiles [5]. Note also that the Euclidian distance-based SOM

algorithm implicitly clusters correlated profiles together in different regions of the SOM. The correlation map shown in Figure S 9 below illustrates this property. Hence, distance-based clustering enables subsequent analysis to identify correlated gene sets.

The variance map shown in Figure S 7b characterizes the variability of each metagene profile by appropriate color coding. One can extract the complementary information by calculating the variability for each tissue-sample in terms of the respective standard deviation,

$$SD_m^{tissue} = \sqrt{\frac{1}{K-1}\sum_{k=1}^{K}\left(\Delta e_{k,m}^{meta}\right)^2} \;. \qquad (6)$$

The bar plot in panel e of Figure S 7 shows the variability of the expression pattern in a tissue-specific fashion. Interestingly, pancreas (endocrine tissues, red), liver (homeostasis, dark yellow), testis (sexual reproduction, pink) and T- and B-cells (immune system, blue) reveal large variability of the expression profiles within their tissue categories. Recently, similar variability measures based on entropy metrics of SOM metagene expression profiles revealed subtle transitions between stages of organogenesis [6]. Metagene expression pattern thus might provide a suited framework for the quantitative characterization of global expression properties to describe different modes of gene activity in the context of cellular differentiation, organ development and also heterogeneity of the tissue samples.

6. Differential expression summary maps

The texture of the SOM visualizes 'local' expression properties in terms of spots due to high and low expression levels in the individual tissues. For an overview about all observed spots we calculate two types of integral 'master' maps characterizing over- and underexpression. Firstly, the metagene peak map shown in part a (overexpression) and b (underexpression) of Figure S 8 accentuates the maximum and minimum values of the metagene expression profiles, respectively. This map plots the metagene profiles in one common scale. They allow discriminating between subtle differences of the amplitudes of the maxima and minima considered. Here the expression maxima and minima are scaled in a sample-specific fashion which virtually amplifies spots referring to local maximum/minimum values in the metagene expression profiles. For example, one finds differently colored spots along the diagonal line in part a of Figure S 8 (spots B to E) which refer to maxima of different amplitude in the respective metagene profiles (e.g. the amplitude of spot C clearly exceeds that of spot B).

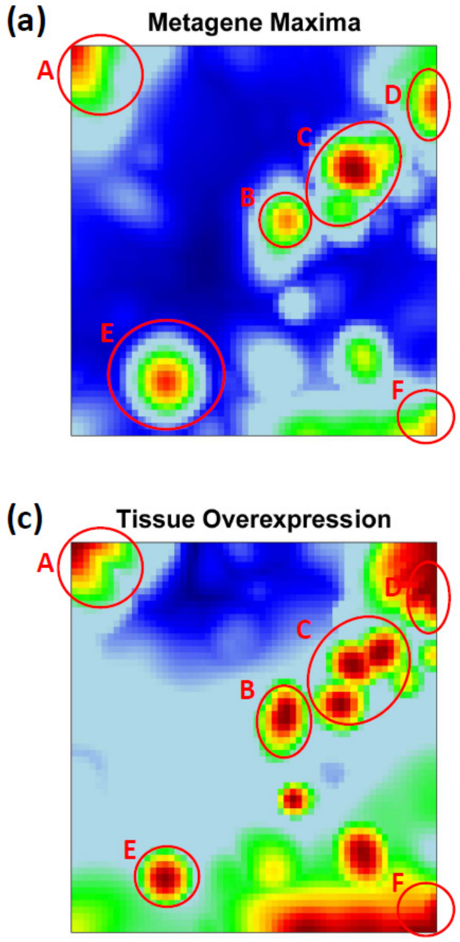

Figure S 8: Grouping genes: Metagene maximum (a) and minimum (b) maps and tissue over (c) and underexpression (d) maps. Red/maroon spots mark overexpression/maxima, blue ones underexpression/minima. Selected spots are marked by letters (capital and lower case letters refer to maxima and minima, respectively). The maximum/minimum maps use a unique scaling for metagene expression whereas the over/underexpression map integrates tissue-specific spots from different scales. As a consequence they show a larger number of spots than the former ones.

Alternatively we plot the sample overexpression overlay maps which transfer either the over- or the underexpression spots observed in the samples into one master map (part c and d of Figure S 8 respectively; see also the main paper). Here, the respective maximum and minimum values observed in one of the samples scale equally showing, for example, equally colored spots along the diagonal line in part c of Figure S 8 (spots B to E, e.g. spots B and C are of equal amplitude). Note also that the overexpression spot C decomposes into three subspots which however strongly differ in their

amplitude in the original expression profiles (compare spot C in Figure S 8c and a). Both types of integral master maps thus reflect similar properties however in a complementary fashion, either with the focus on their absolute amplitude in common scale or on the identification of maxima and minima in the individual SOM maps independent of their amplitude.

7. Correlation maps

The spot-like texture of the SOM results from the large similarity of the profiles of metagenes in adjacent tiles of the SOM image which however decreases with the mutual distance between the tiles in the SOM mosaic. So far we considered the differential expression (over- or underexpression) of the metagenes to identify the spots in the integral spot maps. One can also apply a different metrics based on the mutual correlation of the metagenes. Particularly, we used the following algorithm to determine groups of correlated metagenes in the SOM:

(i) The Pearson correlation coefficients, r_{ij}^{meta} (i, j= 1…K) are calculated for all pairwise combinations of metagenes.

(ii) Their maximum value $r_{IJ}=\max(r_{ij}^{meta})$ defines a pair of 'source' metagenes at positions i,j=I,J. They typically refer to neighbored tiles in the SOM.

(iii) Then, the source metagenes serve as condensation nucleus for the associated group of correlated metagenes which comprises all metagenes meeting the condition , $\min(r_{I,j}, r_{J,j}) > r_{threshold}$ where the threshold value for the correlation cluster is typically set to $r_{threshold}= 0.9$.

(iv) The metagenes of this group were excluded from further analysis which starts again with step (ii) to determine the next group of correlated metagenes by processing the remaining metagenes.

Steps (ii) – (iv) were repeated until all metagenes are clustered into groups of at minimum one member. The obtained groups of correlated metagenes are differently color-coded in the correlation summary map shown in Figure S 9.

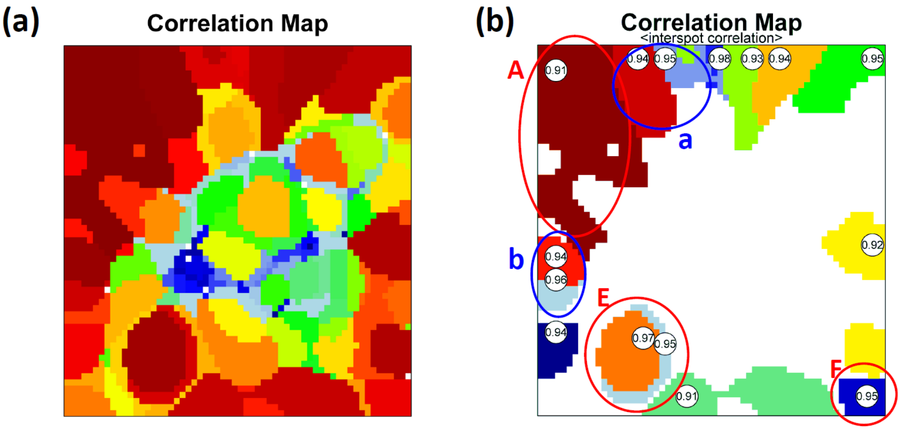

Figure S 9: Groups of correlated metagene profiles. Each colored area represents a group of metagenes which strongly correlate each with another with a correlation coefficient of $r> r_{threshold}=0.9$. The left panel shows the whole correlation pattern of all metagenes. The right part shows the 15 clusters of strongest correlation. The mean correlation coefficient averaged over all metagenes of each cluster is given within the circles.

The clustering of correlated metagenes represents a complementary global approach which searches for metagenes of similar profiles. The obtained groups form disjunct clusters in the respective correlation map (Figure S 9). In general, correlation analysis provides a similar cluster structure compared with the integral over- and underexpression maps. The clusters of largest mutual correlations (Figure S 9b) are mostly located in the region of largest metagene variance in agreement with Eq. (5).

Hence, SOM mapping based on Euclidian distance similarity metrics in the training step provides also a characteristic pattern with respect to the alternative correlation metrics. SOM mapping clusters correlated genes of highly variable expression profiles with pronounced maxima, but also genes of virtually invariant profiles. These two groups of genes tend to occupy different regions either along the edges or in the central area of the mosaic image, respectively.

In summary, SOM machine learning provides a two-dimensional pattern of distinct spots each of which constitutes a cluster of metagenes. The metagene profiles of one spot are usually strongly correlated. Such clusters can be identified in different ways using complementary criteria such as the values of differential expression or the mutual correlation between the metagene profiles. In addition one finds also relatively uninformative clusters of highly populated but virtually invariant metagenes in the central area of the map.

8. Filtering metagenes and single genes

We analyzed the tissue data set using three types of filters to reduce the number of single genes and metagenes, namely FC-expression, variance and significance (FDR-) filtering (see Table S 1 and the methodical section of the main paper). In the first case of expression filtering, the full set of absolute differential expression values of all genes (real genes and metagenes) under all measured conditions are ranked and a certain number of topmost genes in the list is considered for further analysis. In variance filtering the ranked list is generated using the variance of the expression profiles of the genes. These filterings improve the sensitivity of downstream discriminant analyses because they remove non- and less-informative weakly expressed, 'noisy' and/or virtually invariant genes from the data set. In the case of significance filtering the false discovery rate (FDR) of the features (metagenes or real genes) is used as filter criterion. Details of the statistical analysis of differential expression using SOM clustering will be presented elsewhere in a separate publication.

Table S 1 shows that the filter criteria, when applied to the metagenes, gives rise to a gene-to-metagene ratio between G/M= 6 and 28, whereas more stringent filters increase the G/M-values. For example, the 100 selected metagenes (FC-filtering) are representative for 1,487 single genes (G/M=14.9). In turn, selection of real genes roughly maintains this relation: The filtered 100 'real' genes distribute over 8 metagenes only (G/M=12.5) which are all enclosed in the 100 members of the metagene list. Hence, both subsets of metagenes after metagene and single gene filtering completely intersect each other reflecting the high degree of correlation between the metagenes and the associated 'real' genes. Figure S 10 shows the areas in the SOM mosaics covered by the filtered features and their mutual overlap after metagene and single gene filtering in terms of Venn diagrams. The left/right part of the figure highlights the selected metagenes/genes per tile of the SOM-mosaic. For example, the FC-3600 filter selects 100% of the metagenes but only 16% of the real genes. These genes accumulate essentially in the same areas of the SOM-mosaic as the metagenes, however when selected using the more stringent FC-1000 filter, which selects 28% of the metagenes only. The FC-1000 single gene filter, in turn, delivers genes which preferentially accumulate in the metagenes selected mostly by the more stringent FC-100 metagene filter (compare the right mosaics in Figure S 10 with the left ones in the respective rows below).

Hence, equal numbers of 'real' genes and of metagenes selected by the respective filters reflect effectively different sample sizes of real genes owing to the G/M-compression. The metagene lists integrate the properties of roughly a tenfold longer list of 'real' genes and vice versa in our particular SOM setting.

With increasing stringency of filtering, whole spot areas and thus also the respective expression profiles are progressively excluded from the list of filtered features. For example, the most stringent FC-100 metagene filter excludes a few areas selected by the FC-1000 single gene filtering thus revealing a decreased representativeness. Variance-filtering essentially provides similar relations between metagenes and real genes as FC-filtering (see Table S 1).

As a third filter criterion, we applied a threshold of significance levels estimated in terms of the false discovery rate (FDR). The FDR-value defines the probability that each of the selected features is a differentially 'null' and thus a false positive one [7]. It applies to single genes and to the metagenes as well. The FDR-value of the metagenes was calculated as the mean value averaged over the FDR of the single genes associated with each metagene.

The number of selected real genes after FDR-filtering is similar for both types of filters (for example, 670 versus 387 for FDR<0.2; Figure S 11 and Table S 1). The single genes spread over a much larger number of metagenes after filtering real genes than the metagenes which are directly selected after filtering metagenes (116 versus 14 for FDR<0.2, Figure S 11). This difference simply reflects the fact that genes selected by the single gene filter might be associated with metagenes which are not selected by the metagene filter (see the mosaics shown in Figure S 11). Hence, the FDR-filter, if applied to single genes, provides a similar number of real genes compared with the respective metagene filter. These genes however spread over a markedly larger number of metagenes and suggest an increased representativeness. In other words, significance filtering is roughly symmetric with respect to sample size but asymmetric with respect to 'representativeness' of the selected features. Figure S 12 illustrates the consequences of changed FDR-significance criteria.

Table S 1: Filtering metagenes and real genes

filter	threshold	applied to metagenes #metagenes	#real genes	G/M [a]	applied to real genes #metagenes	#real genes	G/M* [a]
**fold change (FC)[b]**	100	100	1,487	14.9	8 (8/0)[c]	100 (100/0)[c]	12.5
	1,000	1,000	7,770	7.8	127 (127/0)	1,000 (1,000/0)	7.9
	3,600	3,600	22,277	6.2	600 (600/0)	3,600 (3,600/0)	6.0
**variance (Var)[d]**	100	100	1,889	18.9	20 (19/1)	100 (97/3)	5,0
	1,000	1,000	9,924	9.9	126 (124/2)	1,000 (995/5)	7,9
**false discovery rate (fdr)[e]**	0.2	14	387	27.6	116 (14/102)	670 (317/353)	5.7
	0.4	666	6,576	9.8	1,390 (666/724)	7,088 (5,587/1,501)	5.1
	0.5	1,751	13,692	7.8	2,332 (1,751/581)	13,063 (11,812/1,251)	5.6

[a] G/M, G/M*: ratio #real genes/#metagenes. All genes of the filtered metagenes are considered in the first case (G/M). In the second case (G/M*) the metagenes containing the filtered single genes are considered. Consequently not all single genes of the respective metagenes are taken into account and one gets on the averaged G/M>G/M* for the same criterion.

[b] Toplist FC-expression filter: Metagenes/genes are ranked with decreasing FC-value. The number of items indicated on top of the list are selected.

[c] (#in/#out): #in denotes the intersection between the number of metagenes/real genes sampled by filtering the metagenes and real genes. #out is the respective number of genes not sampled by the metagene filter

[d] Toplist variance filter: Metagenes/genes are ranked with decreasing variance of their expression profile. The number of items indicated on top of the list are selected.

[e] False discovery rate (fdr) significance filter, i.e. all metagenes/genes with smaller fdr-values than the indicated threshold are included in the list

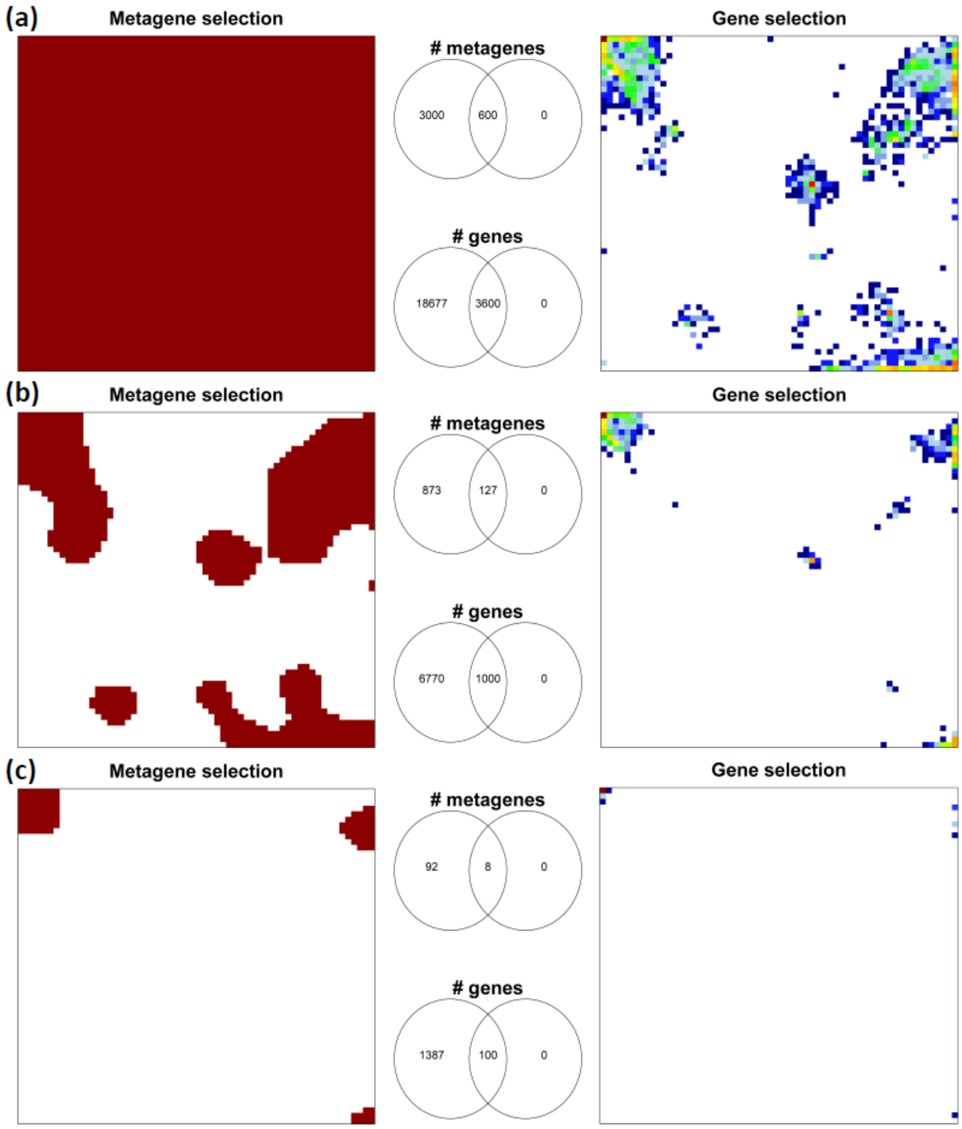

Figure S 10: Filtering genes or metagenes by differential expression: Equal numbers of metagenes (left mosaics) and single genes (right mosaics) are selected using the FC-3600 (a), FC-1000 (b) and FC-100 (c) filters. The brown areas in the left part show the selected metagenes and the colored tiles in the right part the density of single genes (maroon to blue codes high to low densities). The Venn-diagrams illustrate the degree of overlap between the metagenes and genes after metagene and single gene filtering. Note that the FC-3600 filter if applied to single genes (right mosaic in panel a) selects features in the same areas of the mosaic as the FC-1000 filter if applied to metagenes (left mosaic in panel b). The similar result was found for FC-100 and FC-1000 filters if applied to metagenes and single genes, respectively.

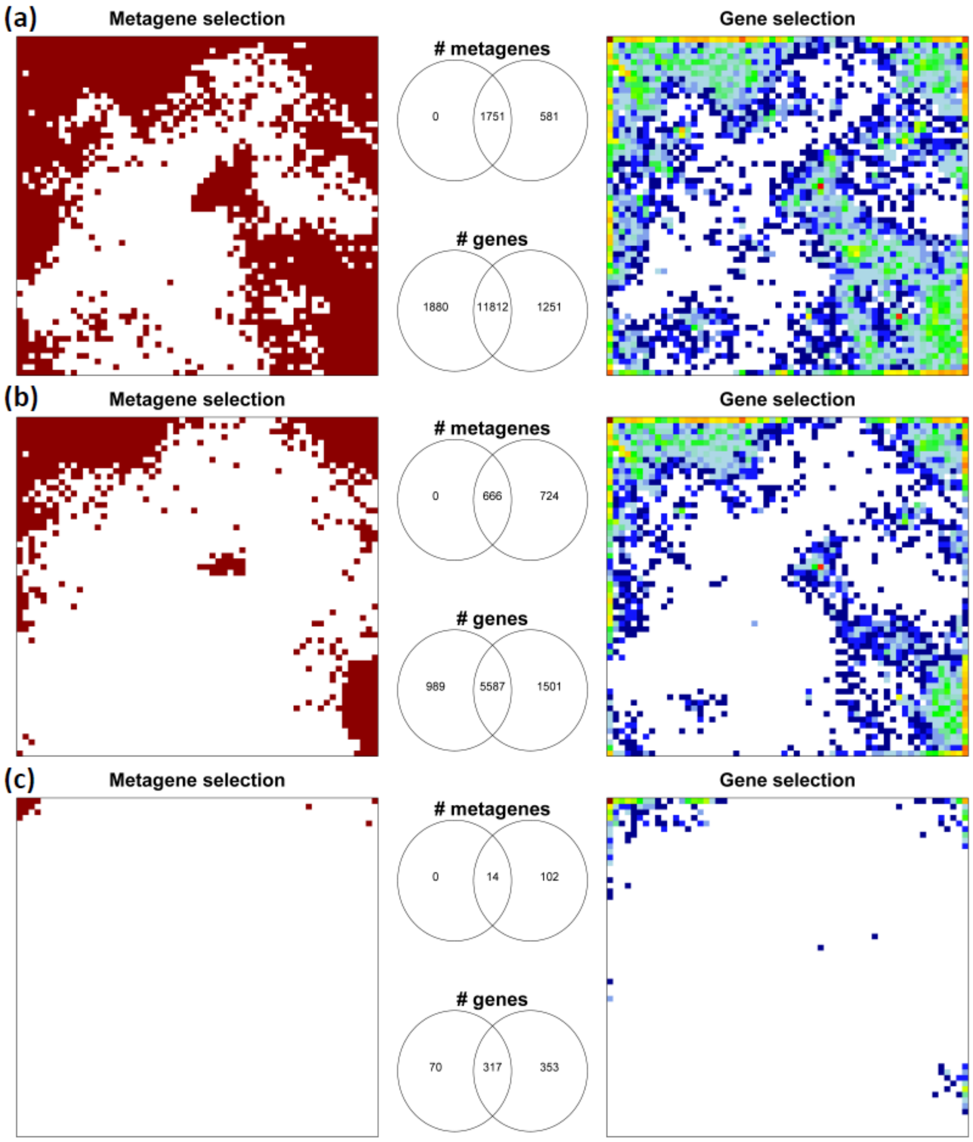

Figure S 11: Filtering genes and metagenes by significance: Equal FDR-thresholds are applied to metagene (left mosaics) and single gene (right mosaics) lists selected using FDR< 0.5 (panel a), FDR<0.4 (panel b) and FDR<0.2 (panel c) filters. The brown areas in the left part show the selected metagenes and the colored tiles in the right part the density of single genes (maroon to blue codes high to low densities) selected by filtering metagene and single gene lists, respectively. The Venn-diagrams illustrate the degree of overlap between the metagenes and genes after metagene and single gene filtering. The single gene filter selects consistently a roughly twice as large number of metagenes and a slightly larger number of single genes than the respective metagene filters.

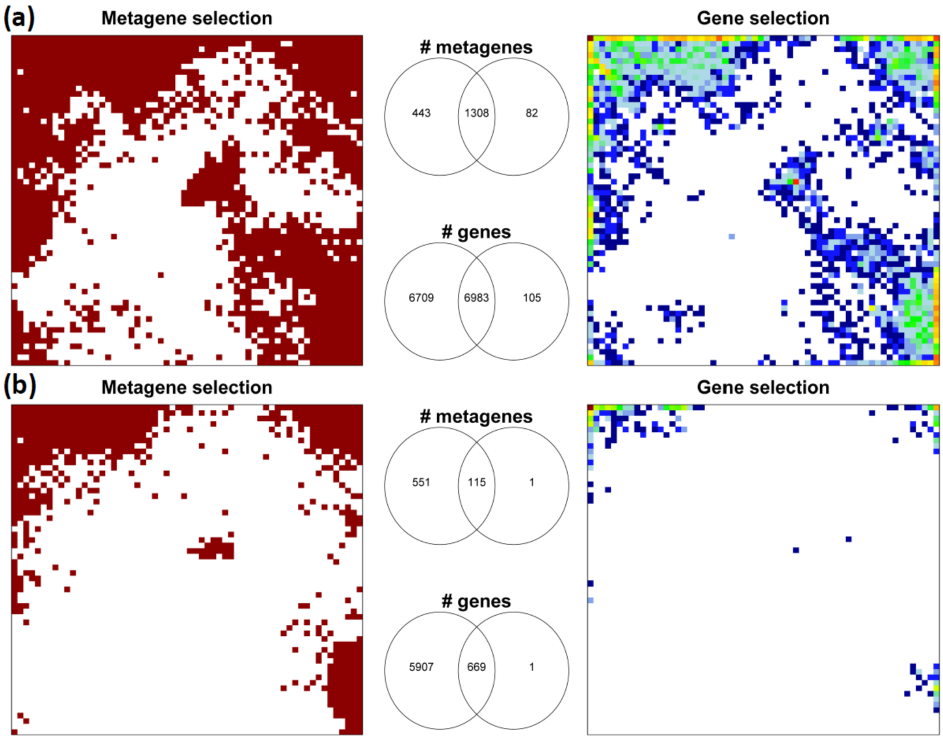

Figure S 12: Analogous to Figure S 11: Comparison of metagene/single gene filters using different FDR thresholds, 0.5/0.4 (panel a), 0.4/0.2 (b). The Venn diagrams indicate that the less stringent metagene filter shifts the number of metagenes and single genes selected towards the metagene filter.

9. Clustering metagenes and single genes

Figure S 13 shows two simple cluster trees with different relative distances between their branching points, L_1 and L_2. The left one characterizes more compact clusters than the right one. It qualitatively explains the difference between the cluster trees obtained from single gene (bottom left) and metagene (bottom right) lists. In the chosen radial representation the cluster trees are projected onto unit circles. It normalizes the mean Euclidian distance between all samples to a common constant. The length of a particular branch in this plot consequently estimates its relative distance which is defined as the ratio of its Euclidian distance divided by the mean value averaged over all branches. The mean length of the 'outer' branches, $<L_1>$, then estimates the mean relative distance between the most similar samples on the lowest level of clustering whereas the mean length of the 'inner' branches estimates the mean mutual distance between the largest clusters. This distance of closest approach is markedly smaller for metagene gene cluster trees than for single gene trees meaning that the observed metagene clusters are more compact as illustrated schematically by the sketch in the middle part of Figure S 13.

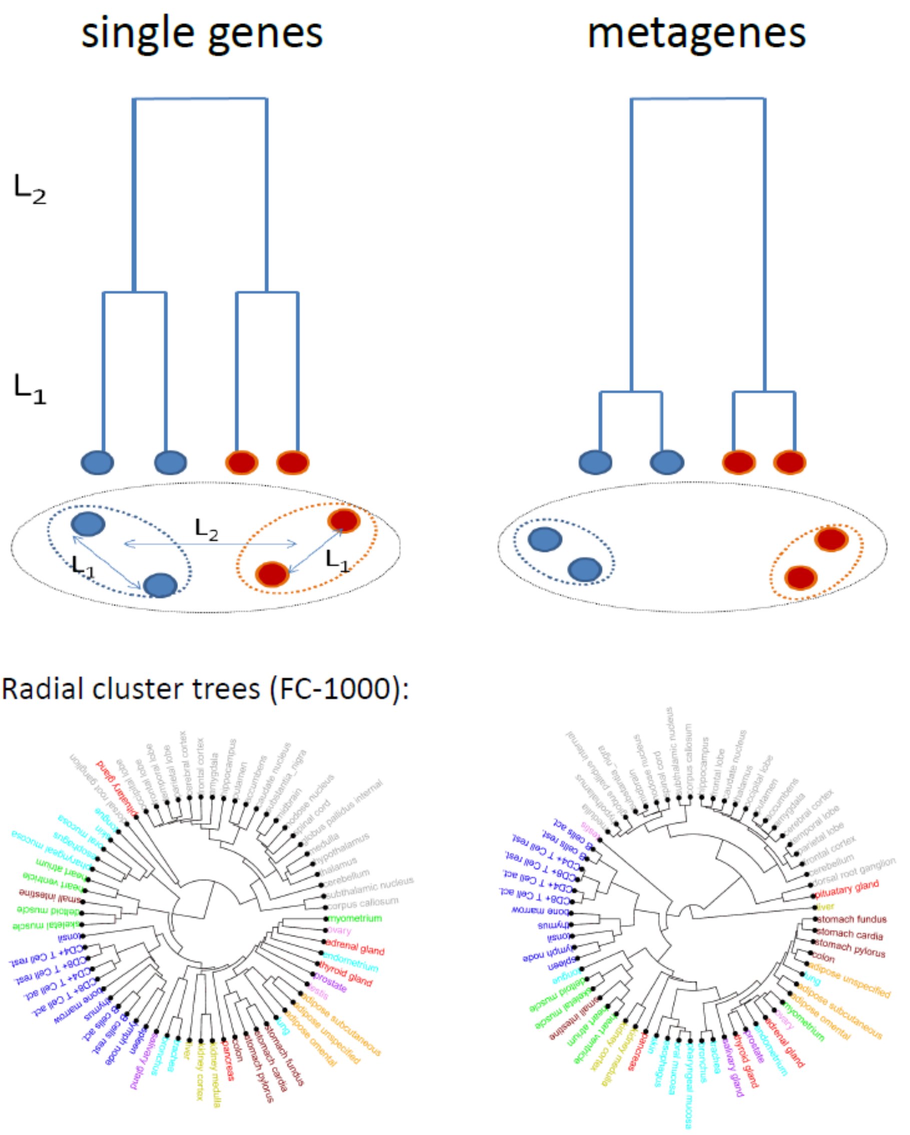

Figure S 13: Schematic illustration how the relative length of the branches in the tree transforms into the compactness of clusters: This distance of closest approach is markedly smaller for metagene gene cluster trees than for single genes meaning that the observed metagene clusters are more compact as illustrated schematically by the sketch in the part above.

10. Selecting metagenes using alternative methods: NMF, HC and correlated sets

We analyzed the tissue data set using three alternative supervised clustering methods: non-negative matrix factorization (NMF, see [8-10]), hierarchical clustering (HC, see [11]) and correlated gene set clustering (CGS, [12-13]). The number of clusters was set to ten in correspondence with the number of overexpression spots detected in the SOM images.

Figure S 14 illustrates the distribution of the genes of the five leading clusters in the SOM-images. NMF generates relatively diffuse clusters which spread over wider areas of the SOM. The first two HC clusters also show diffuse patterns whereas the remaining ones localize in relatively small areas of the map. Finally, CGS also generates localized but partly redundant clusters: Three out of five of them occupy the top left corner of the map which was assigned to genes overexpressed in nervous tissues. The cluster heatmaps in Figure S 15 further confirm this observation: The genes which are specifically overexpressed in nervous tissues are captured by at minimum five of the ten CGS-clusters. HC generates two to three of such ‘nervous system’-clusters whereas SOM provides only one spot which collects virtually all genes overexpresssed in nervous tissues. Note also that SOM and HC show clusters of genes specifically expressed in muscle tissues whereas CGS is unable to collect these genes into a separate cluster. In contrast, the NMF-clusters are clearly not redundant but, on the other hand, most of them are overxpressed in diverse tissue categories and thus unspecific for these tissue groups.

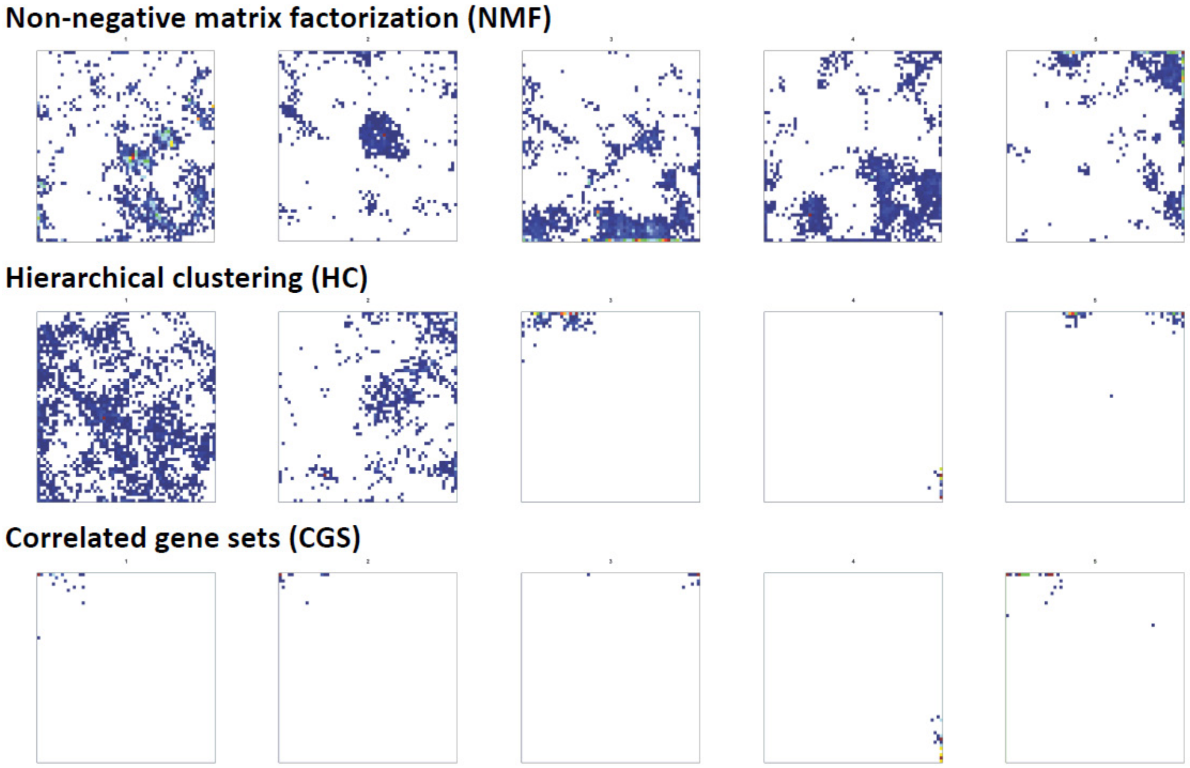

Figure S 14: Cluster-specific population maps of the five leading clusters obtained by alternative methods. SOM-clusters occupied by single genes from the respective clusters are marked by dark dots.

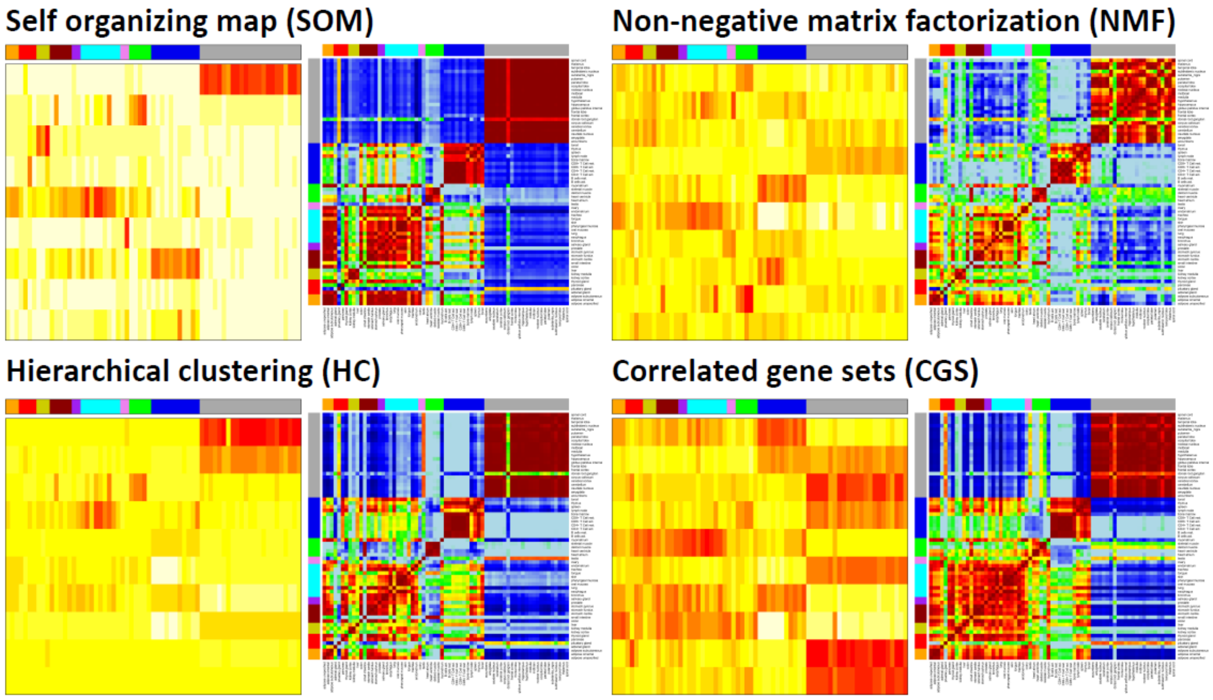

Figure S 15: Metagene cluster heatmap (left part) and PCM (right part) of the metagene expression obtained from different methods. The cluster heatmap visualizes the expression of the metagenes referring to the ten clusters used in all tissue samples. The color bar on top of the heatmap assigns the tissue categories. The PCM illustrates sample-to-sample similarities as seen by the metagenes obtained using the different clustering methods.

Thus SOM clustering obviously outperforms the alternative methods in terms of representativeness with respect to the tissue categories studied. The redundancy of clusters collecting genes which are highly expressed in nervous tissues provided especially by CGS and, to a less degree, by HC can be attributed to the relatively large number of genes in this cluster as discussed in the context of data filtering. Note that SOM removes this redundancy by appropriate re-weighting of these genes as argued in the main paper.

Note that SOM, HC and CGS cluster genes together which show similar profiles in a series of samples using either distance or correlation metrics. Such groups of co-expressed genes can be interpreted in a common functional context based on the guilt-by-association heuristics [14]. Instead, NMF yields a sparse parts-based representation [8] where parts are NMF-metagenes which can overlap and thus expose the participation of single genes in multiple biological processes [9]. Particularly, NMF decomposes the gene expression patterns as an additive combination of NMF-metagenes whereas SOM, HC and CGS use a decomposition that insists mutual exclusion of features. In other words, NMF-metagenes are non-specific for single tissues and tissue categories per definition since they imply an alternative context dependency. The functional meaning of this polysemous decomposition of NMF in comparison with the exclusive guild-by-association decomposition will be addressed separately. Figure S 16 compares enlarged versions of the overexpression heatmaps of SOM- and NMF-metagene clusters together with the top-three overrepresented gene sets. NMF-metagenes enriched with genes related to chromosome function (2nd cluster from the top), to ribosome function (8th) and to translational initiation (10th) spread over several tissue categories.

All clustering methods studied produce similar PCMs (Figure S 15) revealing the same three main groups of tissue (nervous, immune system and the remaining diverse group) as correlated clusters along the diagonal line. SOM-clustering clearly outperforms the other methods in terms of the contrasts of the maps showing the largest gradient of the correlation coefficients. Figure S 16 further supports this finding: Intra-category correlation coefficients of the SOM-metagenes show the sharpest distribution near unity and inter-category correlation coefficients of SOM and HC show the sharpest distribution near minus one. These differences become even more pronounced for the respective

metagene-correlations of nervous tissues which are calculated separately as described in the main paper.

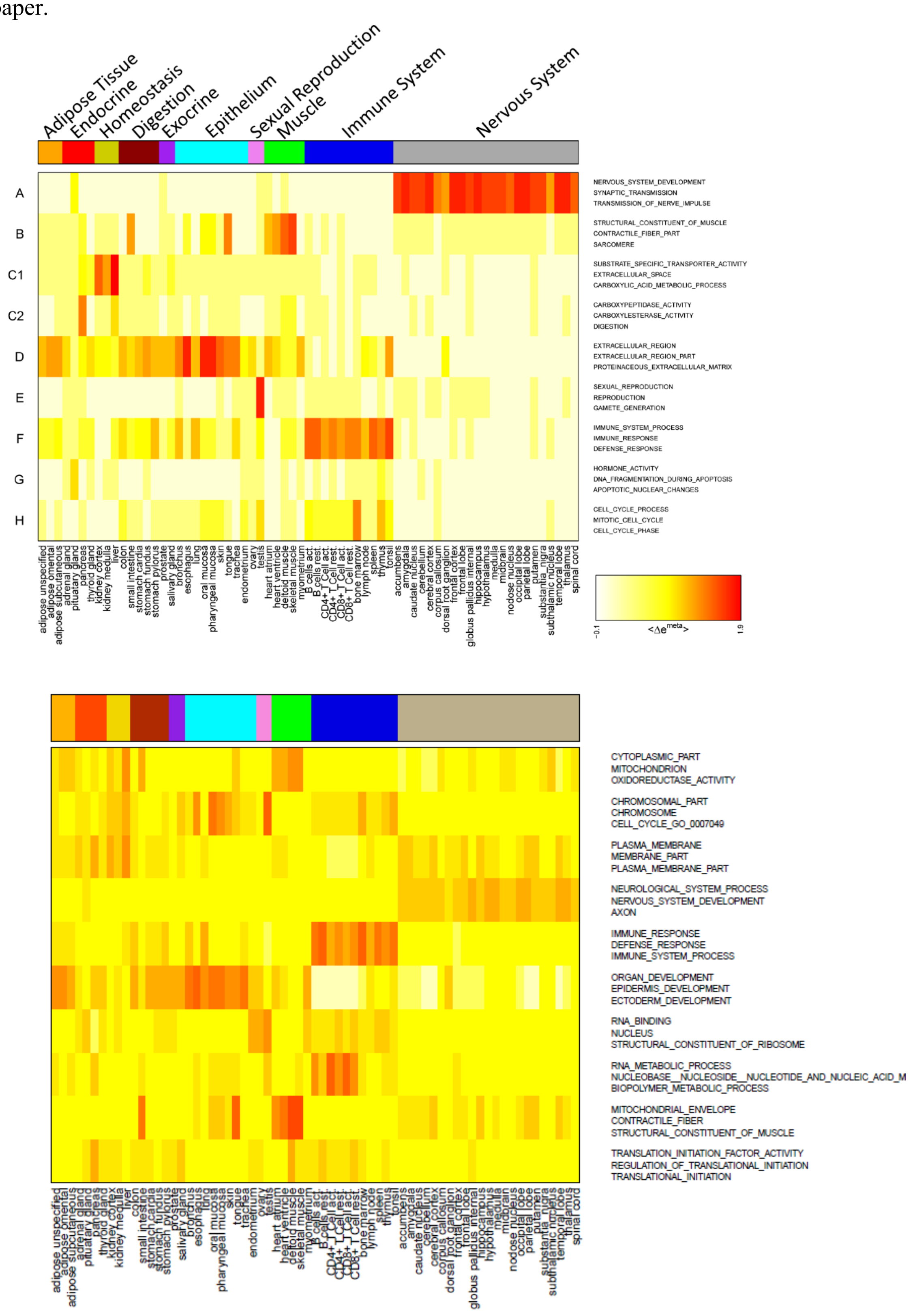

Figure S 16: Cluster overexpression heatmap of SOM- (part above) and NMF-metagens. The right part lists the three leading gene sets overrepresented in each of the clusters.

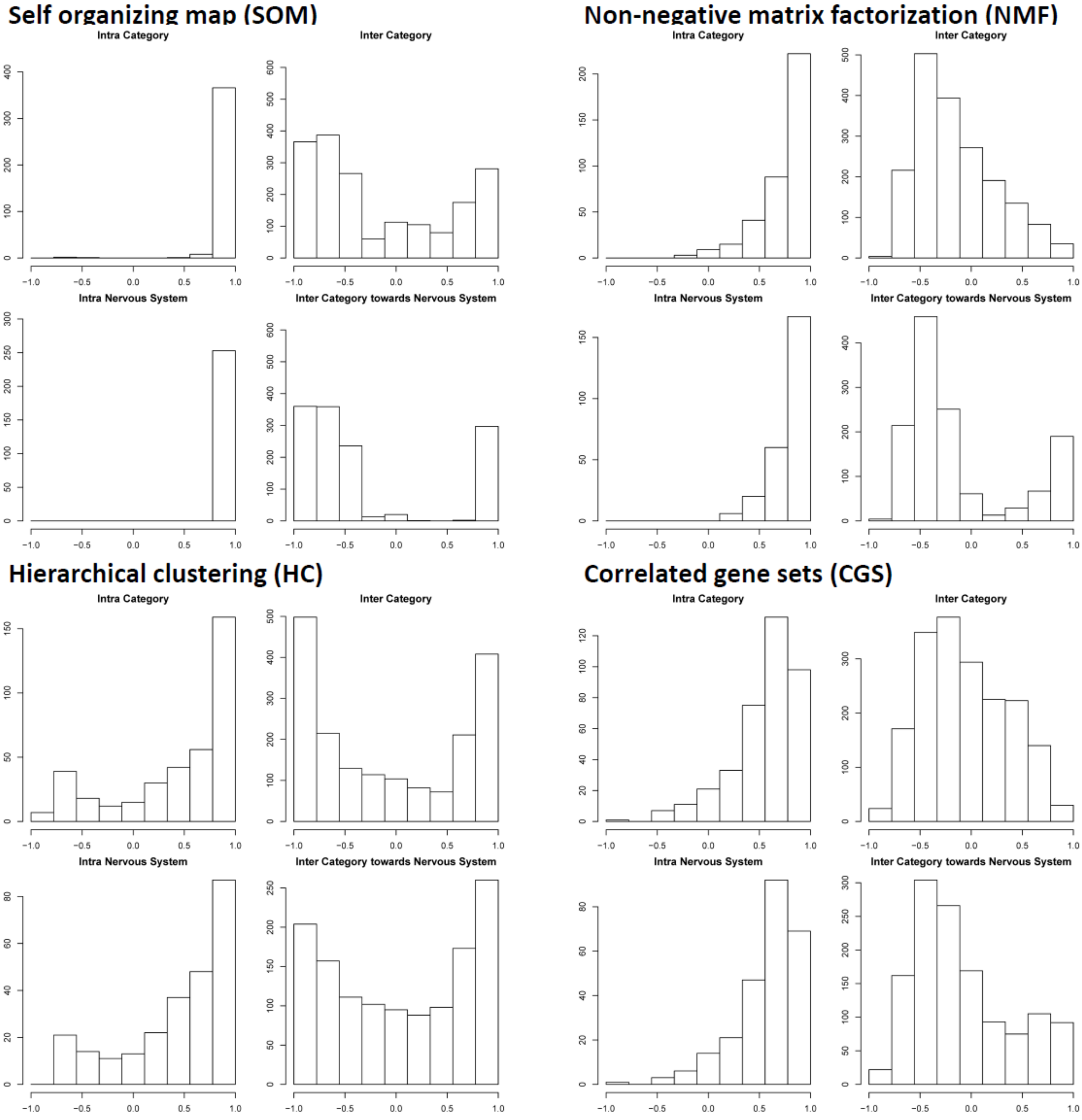

Figure S 17: Histogram of Pearson correlation coefficients for all pairwise combinations of metagene expression profiles between tissues of the same (intra category) and of different (inter category) categories. Intra- and inter category histograms are calculated for all tissues and for nervous tissue only using different clustering methods. The respective histograms of the SOM method are taken from the main paper.

11. Sample- and spot-related similarity trees

We visualized the sample-to-sample correlation pattern of metagene expression using the pairwise correlation map (PCM, see the main paper). Alternatively, one can plot the maximum spanning tree (MST) which is constructed as unidirectional graph connecting samples of strongest mutual correlations of their metagenes (Figure S 17). The MST reveals the predominant similarity relations between the tissues which are not clearly evident in the PCM and/or ICA plots. Most of the tissue categories cluster together along the MST-backbone and/or in distinct side branches with a few exceptions: For example, small intestine is located adjacent to muscle tissues on one side and to tongue on the other side owing to the presence of the muscle related metagene overexpression spot B in the respective SOM as discussed in the main paper. Tissues showing spot D related to epithelium such as myometrium, ovary and adrenal gland are found in one branch together with adipose tissues also showing spot D. Thyroid gland and stomach fundus group together with homeostasis tissues into one branch due to the common presence of metagene spot C related to homeostasis. On the other hand, pituitary gland is also part of the cluster of nervous tissues because it strongly expresses the nervous spot A in its SOM.

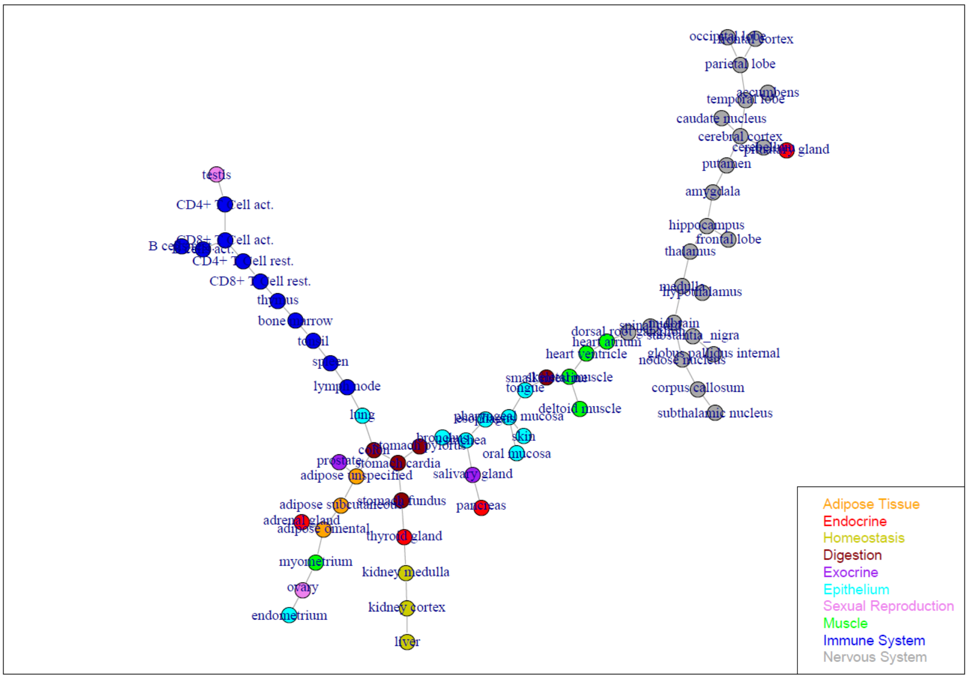

Figure S 18: Maximum spanning tree (MST) of the tissue samples studied (sample tree). The MST is calculated using the correlation matrix of the metagenes shown in the PCM (see main paper). Nervous and immune systems tissues aggregate into well separated clusters. Note that also the remaining tissues partly segregate according to the predefined tissue categories.

Hence, most of the similarity relations expressed by the MST can be simply rationalized in terms of the spot pattern observed in the different SOM without the explicit need to consider the individual metagenes. This result implies to make use of the particular spot pattern of the SOM images and to establish similarity relations between the tissues on a more coarse level of data reduction. With this objective we generated a ‘spot expression matrix’ of size #spots x #samples in the first step. In this matrix, the spot profile of each tissue is characterized by one column containing the mean expression values averaged over all metagenes of each overexpression spot. Then we construct the spot tree as the respective maximum spanning tree connecting spots of strongest correlation in all pairwise combinations of the row-vectors of the spot matrix, i.e. the spot profiles over all samples. The spot tree consequently characterizes similarities between the spots in terms of their common expression in different tissues. In contrast, the sample tree characterizes the similarities between the samples in terms of the degree of correlations between their metagenes.

Each of the nodes of the obtained spot tree in Figure S 18 thus refers to one particular spot. Its position and size is depicted in a small mosaic together with a pie chart the sectors of which visualize the tissues expressing this particular spot. The spot tree consequently visualizes the specificity of each

spot to characterize only one tissue category, such as spot A or E which are found exclusively either in nervous tissues or in testis, respectively. Contrarily, the highly degenerated spot D at the opposite end of the tree is commonly expressed in most of the tissue categories.

Note also that the marker spot F for immune system tissues is located in the centre of the backbone of the spot tree in Figure S 18 whereas immune system tissues occupy a peripheral position in the tissue tree in Figure S 17. This difference shows that the overall metagene expression profile of immune systems tissues is relatively unique whereas the selected immune spot F is commonly found in several tissue categories. For muscle tissues and the muscle spot B one observes the opposite relation, i.e. the spot is relatively specific for muscle tissues, the SOM-images of which are however contaminated by the relatively unspecific spot D to 60%. These examples illustrate the different and partly complementary information of spot- and tissue-related similarity relations.

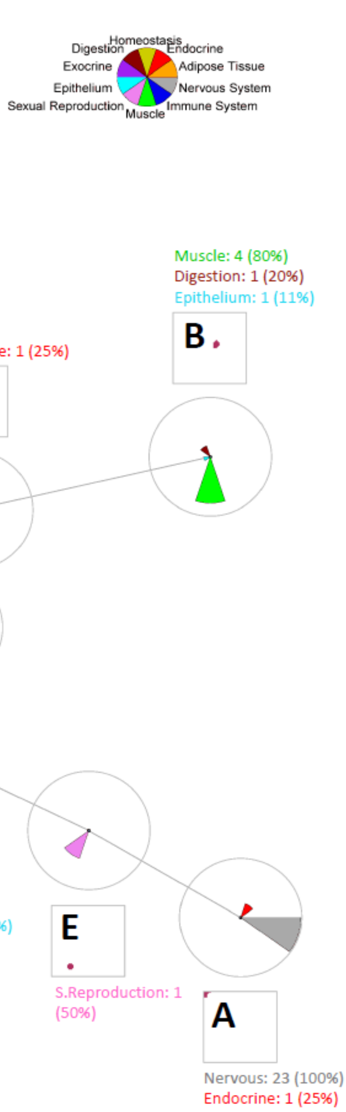

Figure S 19: The spot tree is calculated as the maximum spanning tree of the correlations between the mean metagene expression profiles of the overexpression spots observed in the tissues studied. It characterizes similarities between the spots in terms of their common expression in different tissues in contrast to the sample tree in the previous figure which characterizes the similarities between the samples in terms of the degree of correlations between their metagenes. Each node refers to one spot which is shown in maroon color in the respective quadratic maps and labeled with the capital letters used also in the main paper to assign the spots. The nodes are shown as pie-charts illustrating the tissue category expressing this spot (see the assignment in the top-right part of the figure). The radius of the pie-segments is scaled with the percentage of tissues of the respective category. The radius of the grey circles refers to 100%. For example, spot D is very commonly found in nine tissue category whereas spots A and B are specific for nervous and muscle tissues, respectively.

12. Zooming in: Expression map of nervous tissues

SOM expression profiles show very similar spot pattern for tissues of the same category in some cases. For example, the profiles of nervous system tissues are commonly characterized by highly expressed metagenes in spot A located in the top left corner of the mosaic (see the main paper). Subtle individual characteristics are visible in the blue regions of underexpressed genes (see also the respective SOM images in log log FC scale in the additional material). The PCM-heatmap of all tissue reveals that most of these underexpressed metagenes are specifically overexpressed in tissues of other, 'non-nervous' categories. Hence, nervous tissues are characterized by the overexpression of a specific set of metagenes which, on the other hand, are underexpressed in the other, non-nervous tissues. In turn, metagenes overexpressed in these non-nervous tissues are mostly underexpressed in nervous tissues as a rule of thumb.

To get further insights into the specifics of gene expression of this tissue category we applied a 'zoom-in' step which trains a new SOM using the reduced set of the 20 nervous tissues samples only. The obtained expression images reveal a much more diverse spot pattern of different subcategories of nervous tissues than the images obtained from the whole set of tissues discussed so far (Figure S 19a). For example, the SOM images of cerebral cortex tissues (first row in Figure S 19a: frontal lobe, occipital lobe, parietal lobe and temporal lobe) are clearly different from the other ones. Corpus callosum stands out as an outlier in the second row of profiles in Figure S 19a collecting telecephalon tissues. On the other hand, the spot pattern of corpus callosum resembles that of subthalamic nucleus in the subcategory of diencephalon tissues and also that of three tissues of the mesencephalon category (third and fourth row in Figure S 19a, respectively). Finally, globus palidus (fourth row, mesencephalon tissues) and especially cerebellum (fifth row, rhombencephalon tissues) reveal unique overexpression spot characteristics among all nervous tissues. The overexpression-spot map in part b of Figure S 19 and Table S 2 assign the most prominent overexpression spots.

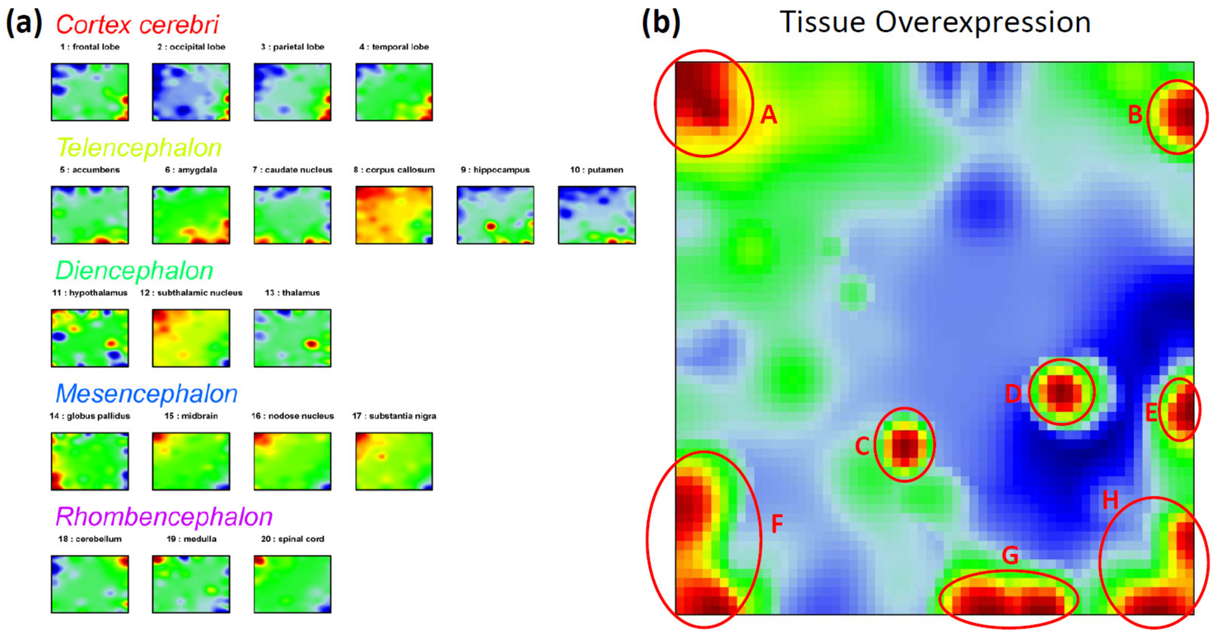

Figure S 20: Zoom-in expression profiles of 20 selected nervous tissues (panel a) and overexpression summary map (panel b). Note that the profiles reveal a much more diverse spot pattern after zoom-in. Selected spots are marked in the summary map in panel b and assigned in Table S 2.

Table S 2: Assignment of selected overexpression spots in nervous tissues.

Spot	Tissue
A	Corpus callosum (8), medulla (19), spinal cord (20)<br>Mesencephalon: midbrain, nodose nucleus, substantia nigra (15-17)
B	Specific for cerebellum (18)
C	Hippocampus (9), (corpus callosum (8)), (subthalmatic nucleus (12))
D	Hypothalamus (11), thalamus (13)
E	Specific for cortex cerebri (1-4)
F	Globus pallidus (14), (caudate nucleus (7))
G	Specific for telencephalon (5-10)
H	Specific for cortex cerebri (1-4)<br>Specific for most telencephalon samples (5-7,9-10)

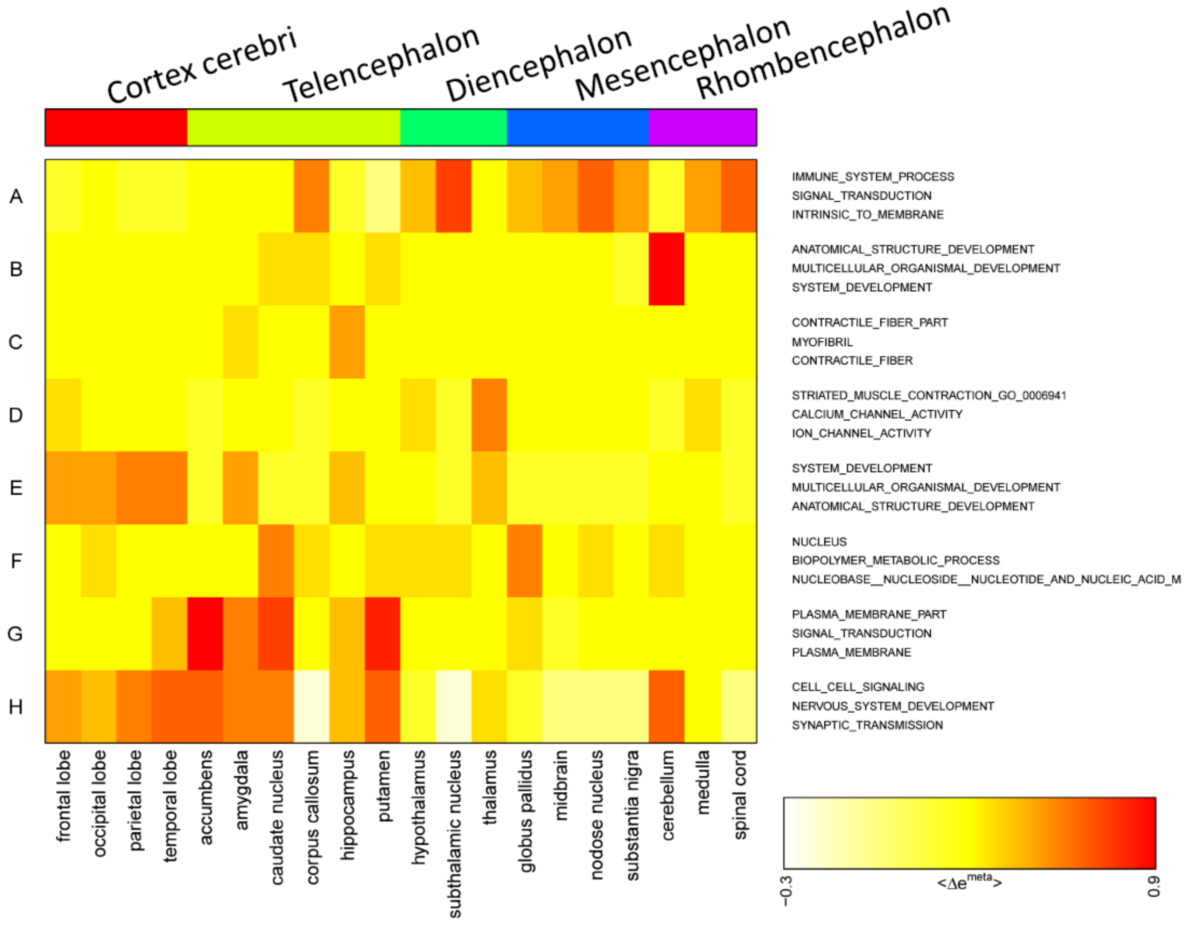

Figure S 21: The overexpression spot heatmap heatmap shows the mean expression of metagenes of each spot in the different nervous tissues. The legend on the right part assigns the three topmost enriched GO gene sets in each spot labeled A – H (see also Figure S 19b).

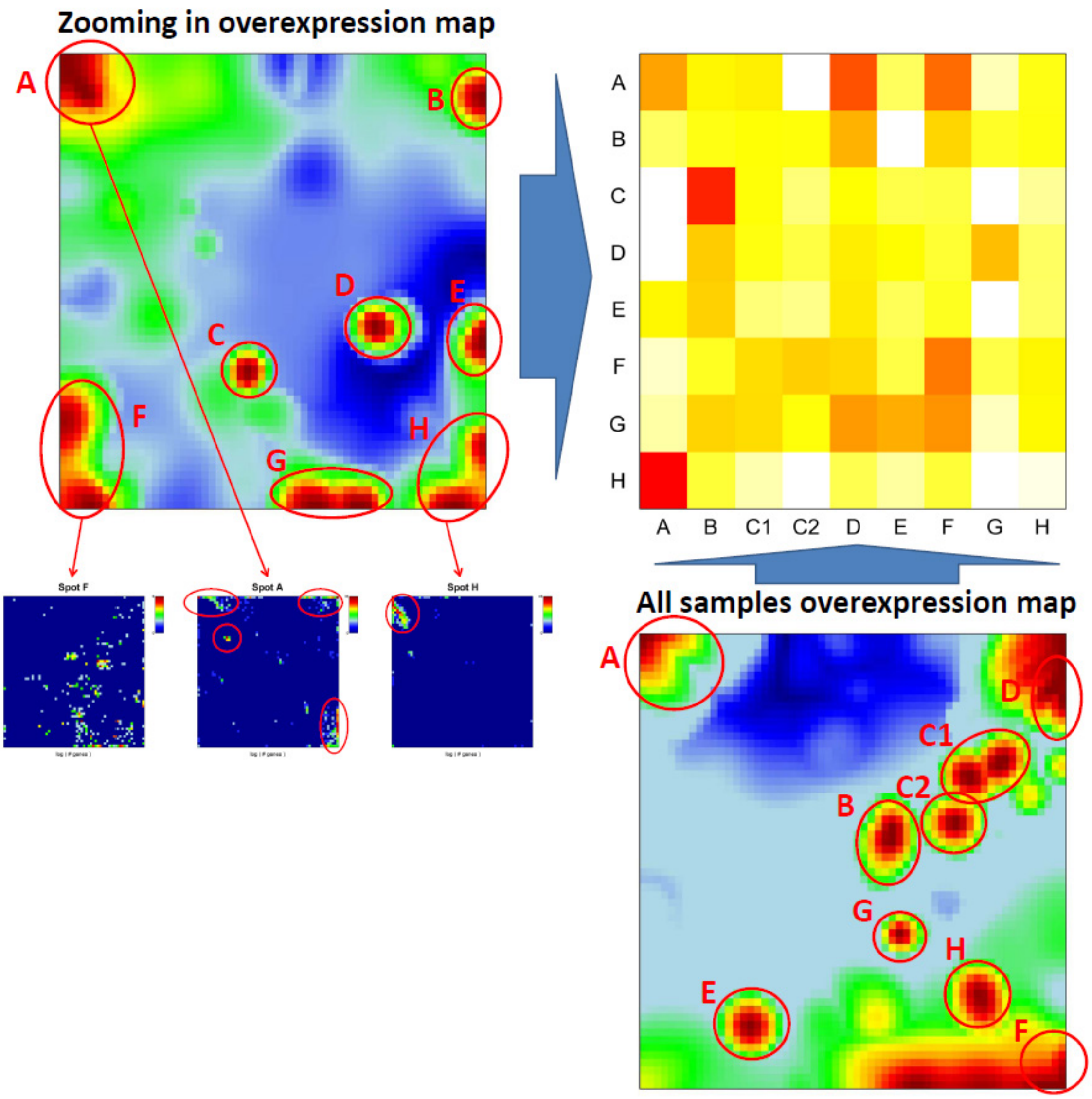

Figure S 22: Spot correspondence between the original overexpression map referring to all tissues (‘all samples overexpression map’) and the zoom-in overrepresentation map generated from nervous system tissues only. The heatmap in the top right part of the figure color-codes the degree of overlap between all pairwise combination of spots from both maps (red: >10%; yellow: 1%>; white: 0%). Note that the genes from spot A in the original SOM referring to the nervous system tissues transfer to a high degree into spot H of the zoom-in map. The three small overrepresentation maps in the left part illustrate the redistribution of genes from selected spots of the zoom-in map into the total map.

The zoom-in SOM of nervous tissues show ‘new’ textures of characteristic over- and underexpression spots which reflect the expression profiles of the tissues of interest more in detail than the original SOM. The newly generated spot pattern differs from the previous one in two respects: Firstly, the metagene profiles cover a reduced range of microstates which increases the resolution of the metagene profiles and results in the partial redistribution of the real genes over the metagene clusters. Secondly, the reference level of differential expression of each gene is defined by its mean value in the pool of all samples considered. It might shift owing to the altered ensemble of samples with consequences for the over- and underexpression pattern. Due to both effects there is no simple one-to-one relation between the spots and the associated metagenes in the original and the zoom-in SOM. Moreover, even equivalent metagene profiles might be located in different regions of the respective SOM due to the independent training runs.

Figure S 21 compares the overexpression summary maps of the previous ‘all samples’ map with the ‘zoom-in’ map of nervous tissues. The heatmap in the figure assesses the correspondence between both overexpression maps in terms of the fraction of common single genes in all pairwise combination of spots taken from both maps. Nervous tissues are characterized by one strong overexpression spot A in the top left corner of the original SOM which contains enriched fractions of genes from gene sets associated with nervous function. This spot transforms with a high degree of overlap into spot H in the bottom right corner of the zoom-in map. Enrichment analysis shows that it indeed contains a largely enriched fraction of genes from the same gene sets which are always enriched in the nervous spot A of the original map.

The overexpression heatmap clearly confirms the enrichment of gene sets directly related to nervous system in spot H (Figure S 20). It also reveals that this spot contains a more diverse expression profile compared with the respective profile of spot A in the nervous tissues (see the main paper). The zoom-in map obviously amplifies subtle details of the expression profile of these genes in the reduced subset selected for zoom-in analysis.

Interestingly, the zoom-in map of nervous tissues also amplifies the expression profiles of gene sets not clearly related to nervous system and not evident in the original overall map. For example, the genes in spot A of the zoom-in map markedly overlap with the genes in spot D and F in the original ‘all-samples’ overexpression map. These spots are related to tissue development and immune response, respectively. Spot A indeed contains an enriched number of genes associated with ‘immune response processes’ but also genes related to ‘signal transduction’ whereas the gene sets related to ‘tissue development’ are not among the top-enriched sets. Note also that the genes from the ‘immune system’ spot F in the corner right below in the original map do not simply shift into spot A of the zoom-in map. Instead, they accumulate also in other spots such as spot F which enriches genes related to ‘nucleus transport’ function. These examples illustrate the fact that the zoom-in step re-distributes part of the genes into metagene expression profiles not clearly resolved in the original map with consequences for the particular enrichment pattern and its tissue specificity. For example, spots A and H of the zoom-in map are overexpressed in different nervous tissues, among others, in mesencephalon (blue) and cortex cerebri (red), respectively (Figure S 20).

Figure S 22 shows the results of different similarity analyses using the 100 topmost differentially expressed metagenes. Essentially three groups of nervous tissues can be identified, namely one containing three telencephalon tissues (olive bars/circles in the PCM/MST plots) and two mixed groups, where one is essentially dominated by the characteristics of cortex cerebri tissues (red) and the second one by the characteristics of mesencephalon tissues (blue). The cerebellum sample is isolated from these groups. These results clearly show that zooming-in largely improves resolution and discrimination between the samples of a selected tissue subgroup.

Taking together, the resolution power of SOM analysis is improved in a zooming-in step which trains the SOMs using a subensemble of samples. Effectively this step adapts the expression profiles of the metagenes to the smaller bandwidth of expression values observed in the subensemble compared with the wider range observed in the whole ensemble of tissues. Note that machine learning maps the multidimensional space of differential expression of the considered samples onto that of the metagenes. It automatically adapts to the maximum amplitudes and to the degree of similarity between the samples considered.

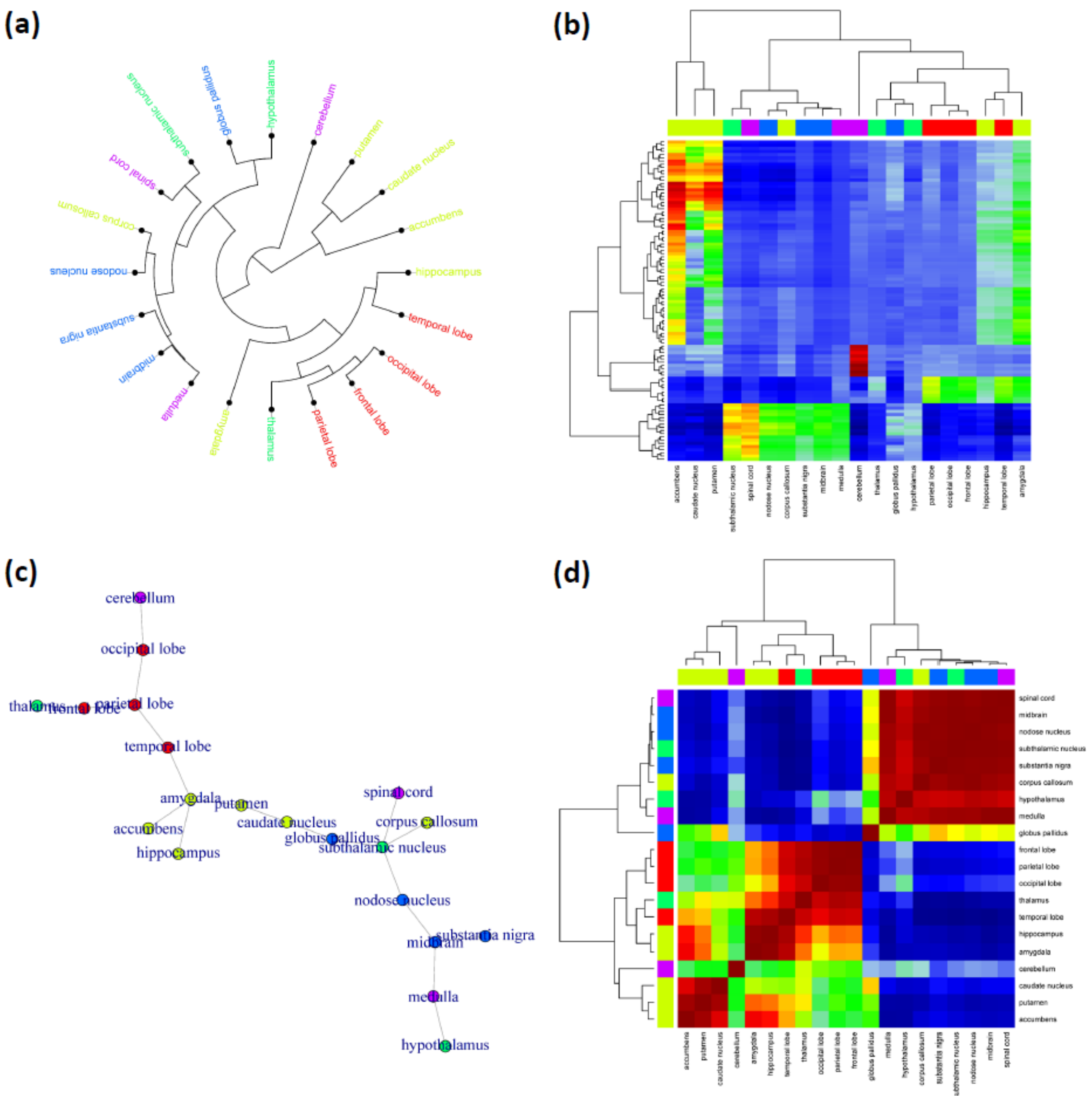

Figure S 23: Zooming-in characteristics of nervous tissues: Clustering tree (panel a), two-way hierarchical clustering heatmap (b), MST (c), and PCM (d) of 20 selected nervous tissues based on the 100 topmost differentially expressed metagenes of nervous tissues. The different categories of nervous tissues are color-coded as assigned by the colors of the captions in Figure S 19a.

13. Zooming in: Expression map of immune system tissues

We applied the zoom-in step to the 11 samples of the immune system category in analogy to the zoom-in of nervous tissues. The expression profiles of these samples shown in Figure S 23a reveal an improved resolution (compare with the original SOM obtained from the whole set of tissues). For example, the spot pattern of lymphocytes and of primary and secondary lymphoid organs can be better distinguished: Spot A is overexpressed in resting T-cells and spot C in activated T-cells only (see Figure S 23b). Spot B collects genes commonly overexpressed in all T-cells. Table S 3 assigns the most prominent overexpression spots to the different immune tissues.

Spot correspondence analysis shows that the genes from the characteristic ‘immune system’ overexpression spot F observed in the original SOM (see the main paper) distribute over 3 -4 new spots after zooming-in (A, B, C, G1, see Figure S 25). The leading gene sets are attributed to ‘immune systems process’ (spot A), ‘signal transduction’ (B), ‘programmed cell death’ (C) and again ‘immune systems process’ (G1) as illustrated in Figure S 24. Spots A, B and partly C are overexpressed in T-cells whereas spot G1 is overexpressed in B cells. Hence, the re-sorting of genes over a new collection of overexpression spots in the zoom-in map splits the gene sets ‘immune systems process’ and ‘defense response’ into two types of genes showing high expression either in T- or in B-cells. In the original SOM both types of genes are found together in the immune spot F. Table S 4 provides examples of such genes which can be partly assigned to T- or B-cell function.

Genes from another spot originally assigned to ‘cell cycle process’ (spot H, see [15]) remain basically localized within one spot after zoom-in which however strongly enriches also genes from other gene sets such as ‘defense response’ (spot H, Figure S 24) which is originally enriched in the immune systems spot F before zoom-in (see [15]). Hence, spot H and also spot G2 collect genes originally distributed over different spots (Figure S 25). Taken together, one observes two tendencies after zoom-in, namely the split of enriched gene sets from one into several spots and the aggregation the gene sets from several spots into one leading one.

The results of downstream cluster- and correlation-analysis are shown in Figure S 26. One- and two-way hierarchical clustering (Figure S 26a,b) reveal close relation of resting and activated modes of B-cells and the two types of T-cells, respectively. This is also confirmed by correlation analysis which provides PCM and MST plots (Figure S 26c and d): lymphocyte subtypes are highly correlated and clearly separated from lymphatic organs.

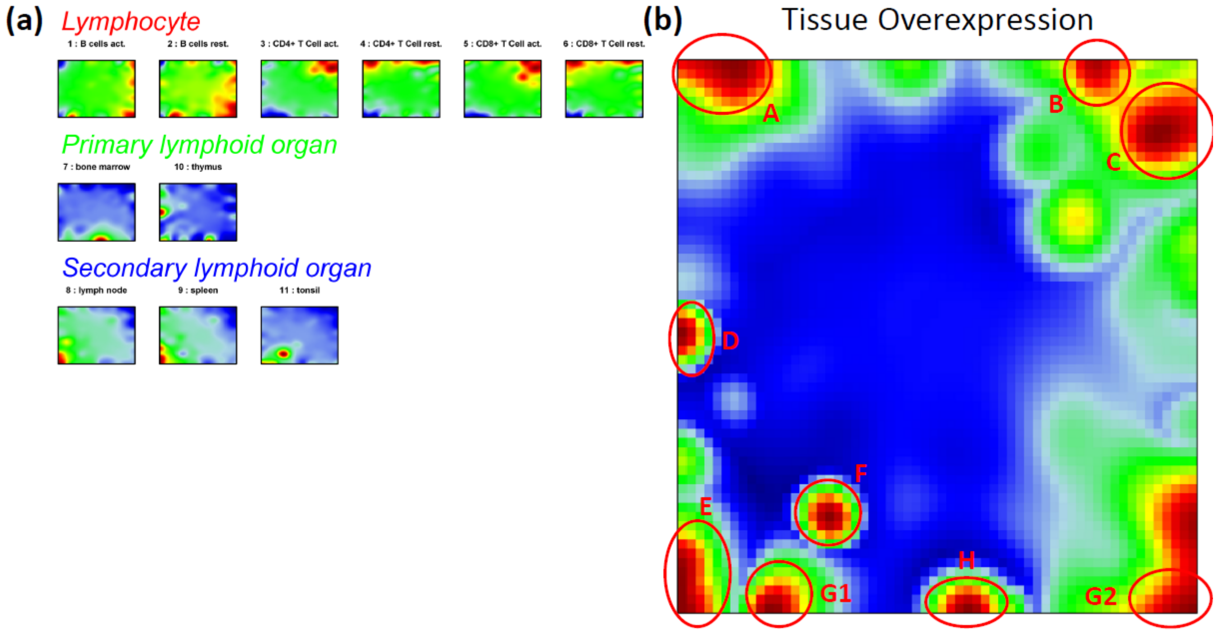

Figure S 24: Zoom-in expression profiles (panel a) and overexpression summary map (panel b) of 11 samples from 3 categories of the immune system. Selected spots are marked in the summary map and assigned in Table S 3.

Table S 3: Assignment of selected overexpression spots in immune system samples (see also Figure S 23).

Spot	Tissue
**A**	Resting T-Cells (4,6)
**B**	All T-Cells: CD4+/CD8+; resting/activated (3-6)
**C**	Activated T-cells (3,5)
**D**	Specific for thymus (10)
**E**	Lymph node (8), spleen (9)
**F**	Specific for tonsil (11)
**G1, G2**	B-cells (1,2)
**H**	Bone marrow (7), thymus (10)

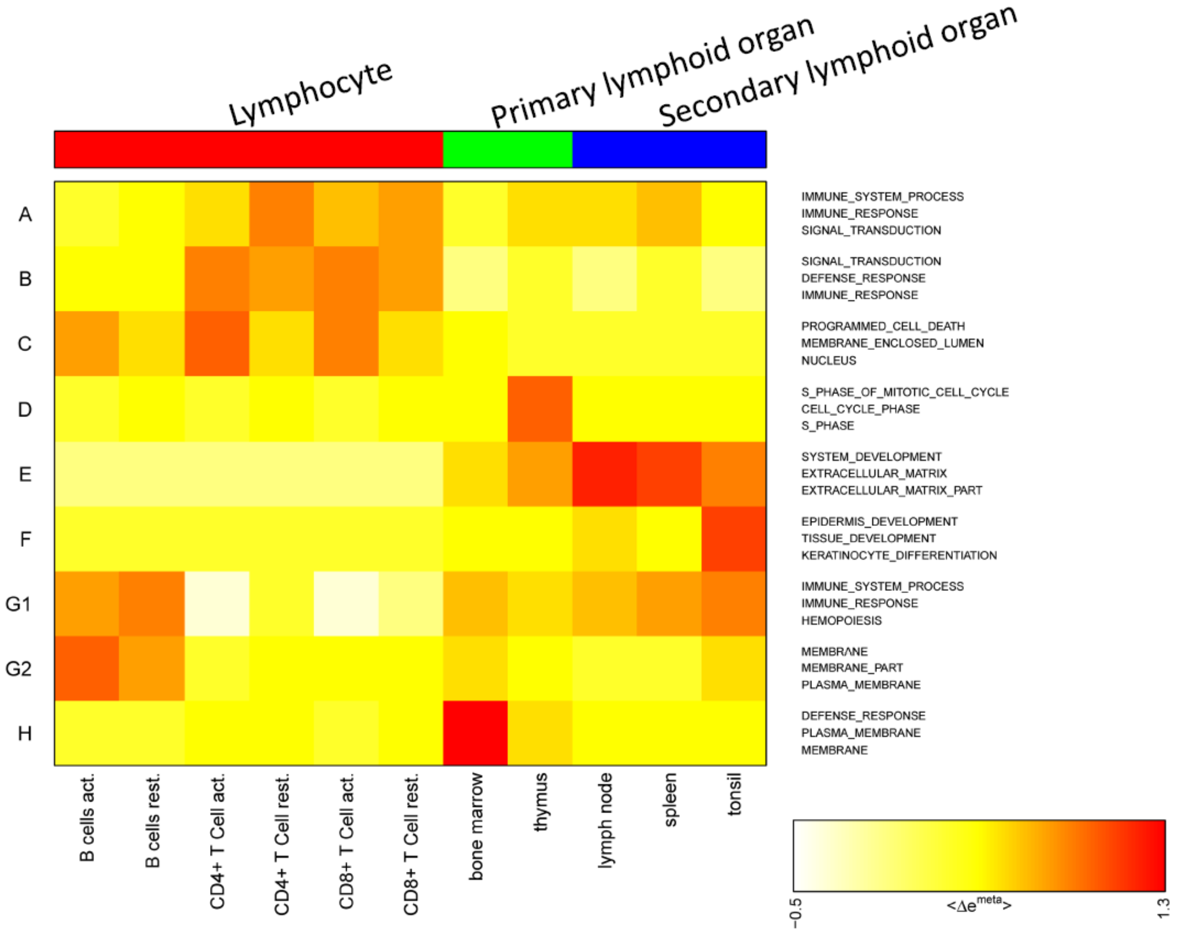

Figure S 25: Overexpression spot heatmap of immune systems tissues. The legend on the right part assigns the three topmost enriched GO gene sets in each spot labeled A – H (see also Figure S 23b).

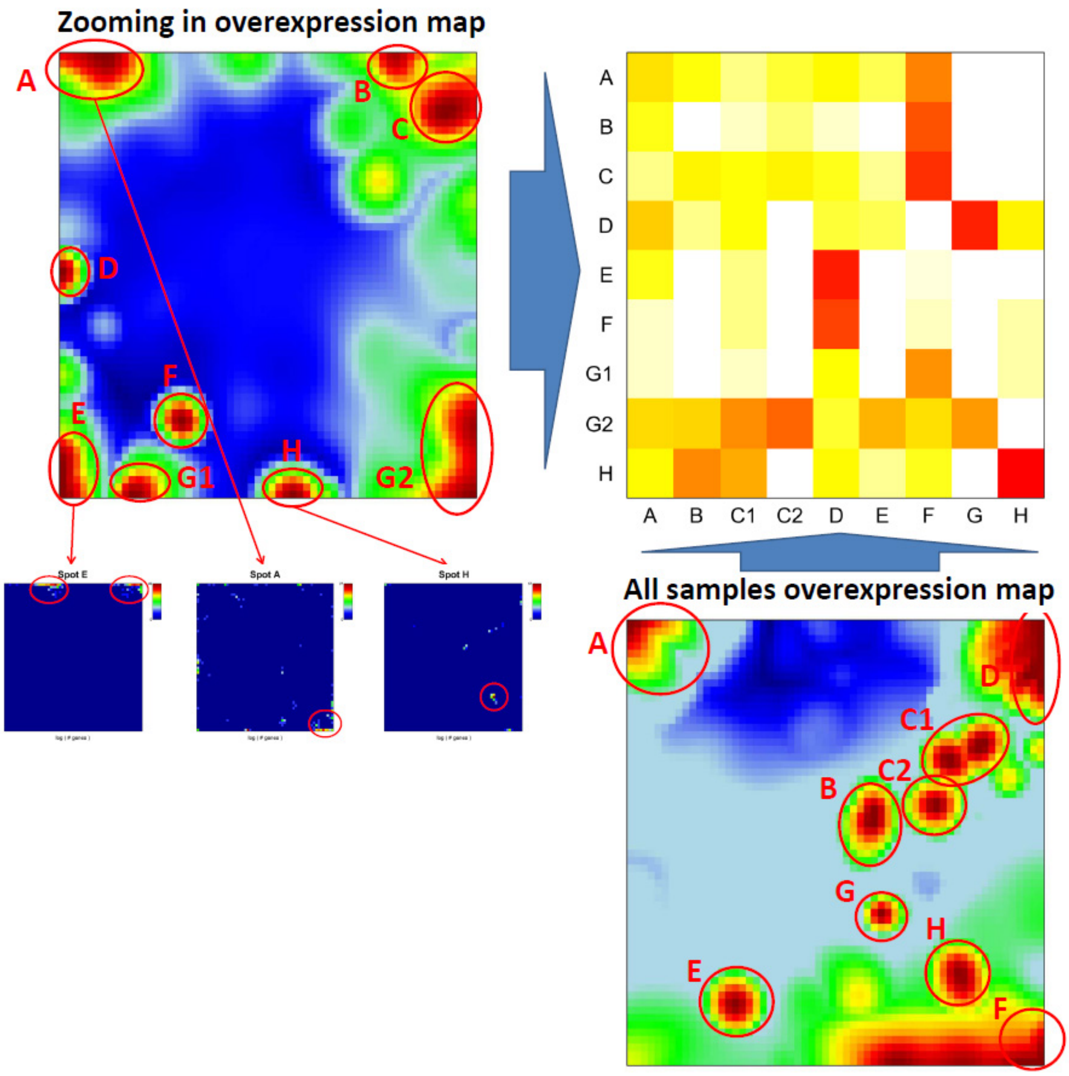

Figure S 26: Spot correspondence between the original overexpression map referring to all tissues ('all samples overexpression map') and the zoom-in overrepresentation map generated from immune system tissues only. The heatmap in the top right part of the figure color codes the degree of overlap between all pairwise combination of spots from both maps (red: >15%; yellow: 1%>; white: 0%). Note that the genes from spot F in the original SOM referring to the immune systems tissues transfer essentially into spot A, B and C of the zoom-in map. The three small overrepresentation maps in the left part illustrate the redistribution of genes from selected spots of the zoom-in map into the total map.

Table S 4: Genes of the gene sets overrepresented in different spots of the zoom-in map

Gene set	T-cells: overrepresented in spot A	B-cells: overrepresented in spot G1
**Immune systems process**	IL8 (interleukin 8)<br>CD3D (T-cell receptor)<br>IL2 (interleukin 2, T-cell growth factor)<br>CCL4 (chemokine (C-C motif) ligand 4)<br>CST7 (cystatin F (leukocystatin))<br>IL7R (interleukin 7 receptor, essential for the differentiation and activation of T lymphocytes)	CCL22 (chemokine (C-C motif) ligand 22)<br>MS4A1 (B-lymphocyte antigen CD20)<br>CD79A (B lymphocyte antigen receptor)<br>CD79B (B lymphocyte antigen receptor)<br>POU2AF1 (POU class 2 associating factor 1, essential for the response of B-cells to antigens)<br>BLNK (B-cell linker)
**Defense response**	GNLY (granulysin, present in cytotoxic granules of cytotoxic T-cells)<br>IL8 (interleukin 8)<br>KLRG1 (killer cell lectin-like receptor)<br>ITK (IL2-inducible T-cell kinase)<br>CX3CR1 (chemokine receptor 1, involved in T-cell differentiation)<br>CCL4 (chemokine (C-C motif) ligand 4)	CCL22 (chemokine (C-C motif) ligand 22)<br>CD40 (TNF receptor resting B cell activation)<br>BLNK (B-cell linker)<br>LY75 (lymphocyte antigen 75)<br>HDAC9 (histone deacetylase 9)<br>IL17RB (interleukin 17 receptor B)

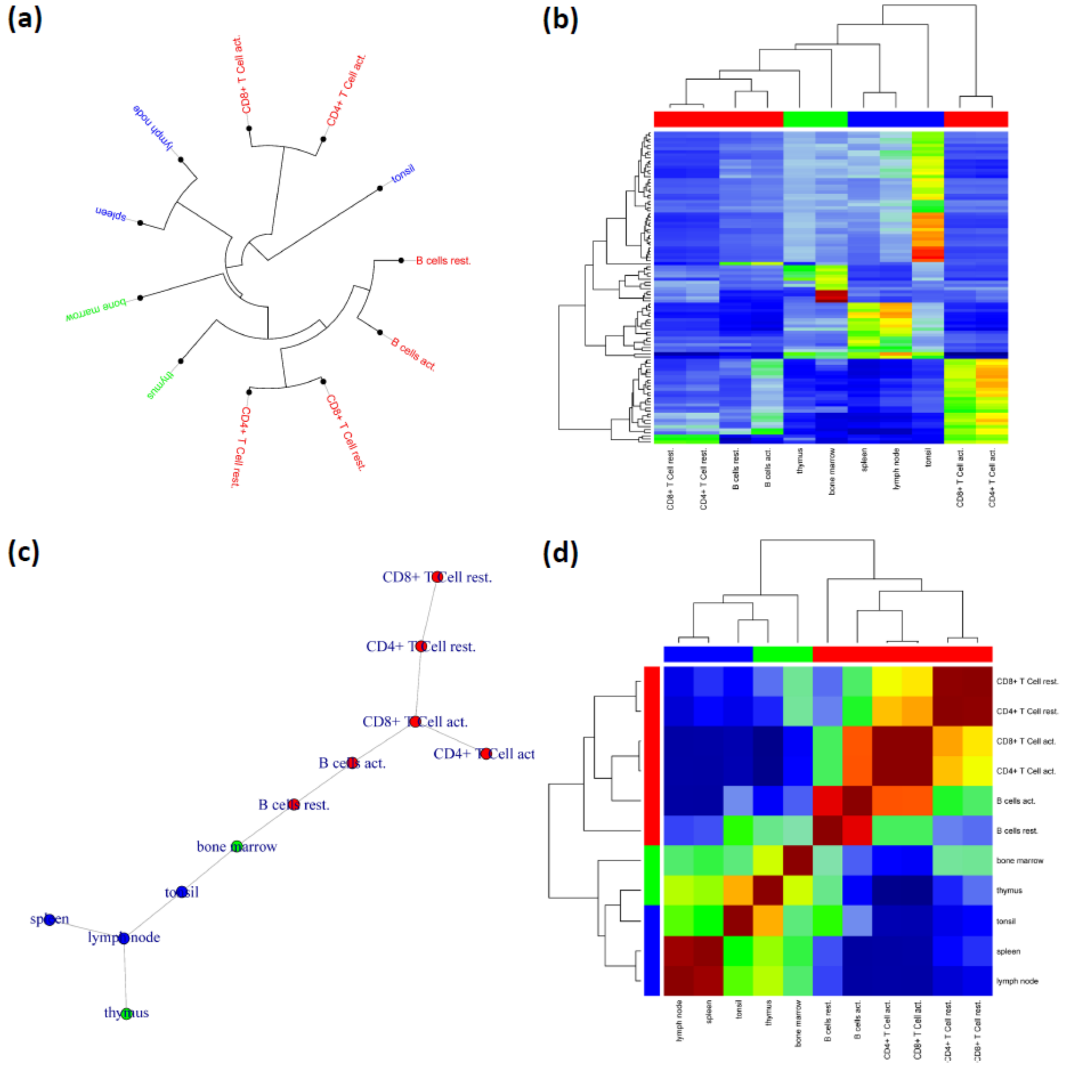

Figure S 27: Zooming-in characteristics of the immune system samples: Clustering tree (panel a), two-way hierarchical clustering heatmap (b), MST (c) and PCM (d) expression fingerprints of 11 immune system samples, assigned to 3 categories, based on the 100 topmost differentially expressed metagenes. The different categories are color coded as assigned by the colors of the text of the captions in Figure S 23.

14. Zooming in: Expression map of 31 diverse tissues

We applied zooming-in to 31 diverse tissues forming one of the three clusters in the ICA plots to better differentiate between the respective samples. This group of tissues is much more heterogeneous than the cluster of nervous and immune system tissues analyzed above. This collection of tissues subsumes the categories adipose, endocrine, homeostasis, digestion, exocrine, epithelium and muscle tissues which cluster relatively tightly together in the agglomerative analyses provided in [15]. Figure S 27 shows the resulting SOM profiles and the overexpression summary map. Compared with the original SOM one finds a higher number of overexpression spots despite smaller number of samples. This supports the conclusion that nervous and immune system samples dominate the metagene differential expression suppressing subtle differences within the set of 31 diverse tissues if analyzed together.

The tissue specific spots B (muscle tissues), C1 (liver and kidney) and C2 (pancreas) of the original map transform virtually ‘one-to-one’ into the spot C, A and B of the zoom-in map, respectively (Figure S 29). They overrepresent essentially the same gene sets as the spots in the original map. Note however, that spots A and B are better resolved in the zoom-in map than spots C1 and C2 in the original map. On the other hand, the original spot D found in different tissues such as adipose and epithelial ones splits essentially into the three spots F, G and H which are selectively overexpressed in adipose tissue (spot F), stomach, lung, trachea and bronchus (G) and other epithelial tissues (H) with the different leading gene sets ‘plasma membrane’ (F), ‘immune system process’ (G) and ‘ectoderm development’ (H) after zoom-in, respectively.

The results of cluster- and correlation-analyses are shown in Figure S 30. Notably, most of the tissues cluster together in agreement with their predefined categories.

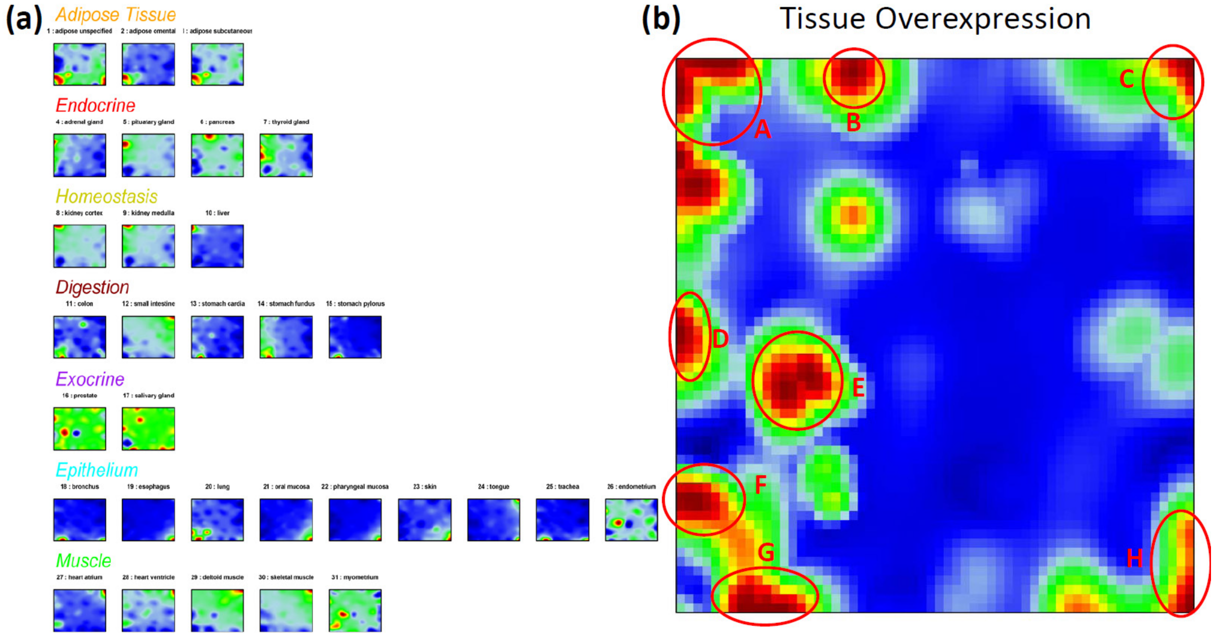

Figure S 28: Zoom-in expression profiles (panel a) and overexpression summary map (panel b) of a group of 31 diverse tissues from 6 tissue categories poorly resolved in the central part of ICA-plots of all tissues. Selected spots are marked in the summary map and assigned in Table S 5

Table S 5: Assignment of selected overexpression spots in diverse tissues (see also Figure S 27).

Spot	Tissue
A	Specific for homeostasis samples (8-10)
B	Pancreas (6)
C	Specific for muscle samples: heart atrium, heart ventricle, skeletal muscle, deltoid muscle (27-30)
D	Thyroid gland (7)
E	Prostate (16), endometrium (26), myometrium (21)
F	Specific for adipose tissue (1-3)
G	Digestion: colon (11), stomach samples (13-15)<br>Epithelium: bronchus (18), lung (20), trachea (25)
H	Specific for epithelium tissues (18-26)

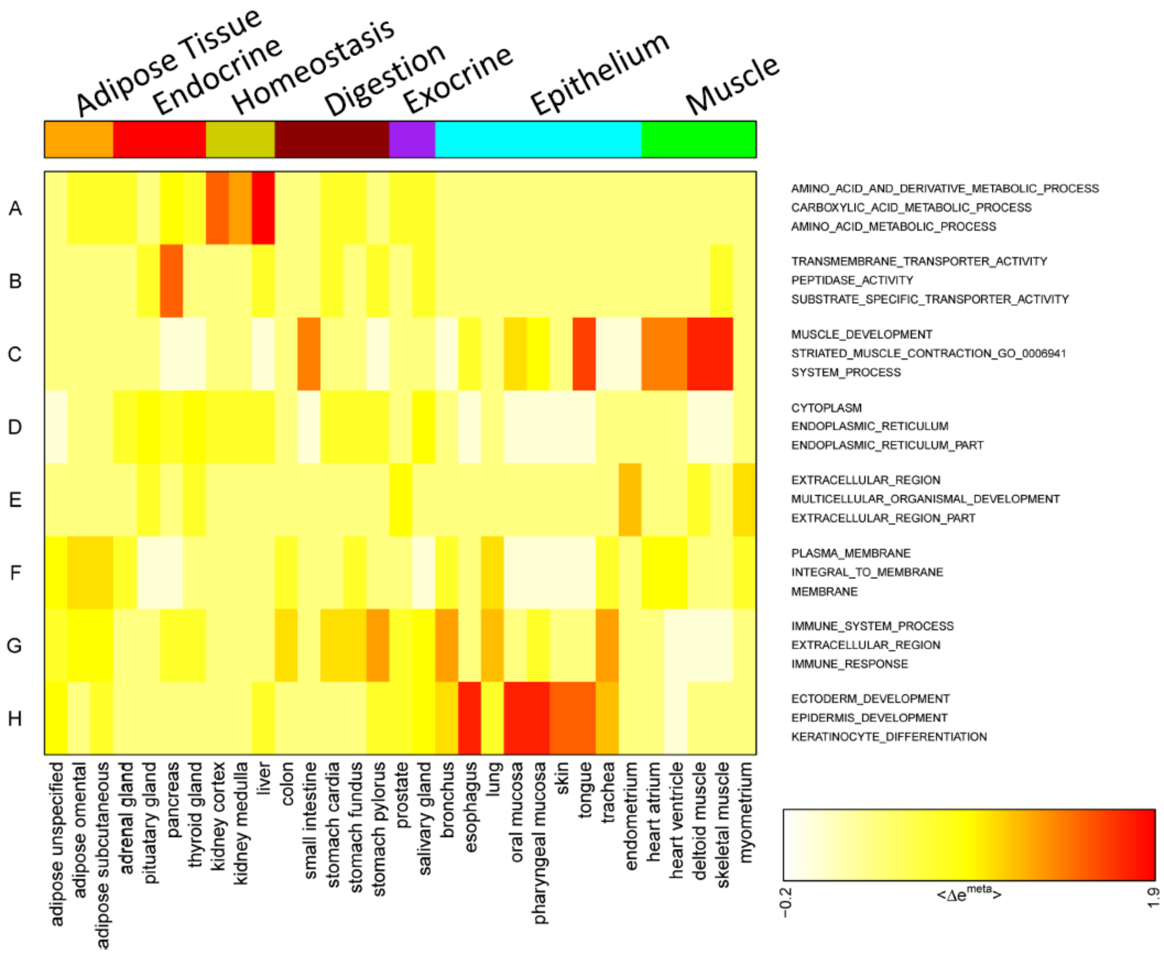

Figure S 29: Overexpression spot heatmap of immune systems tissues. The legend on the right part assigns the three topmost enriched GO gene sets in each spot labeled A – H (see also Figure S 27b).

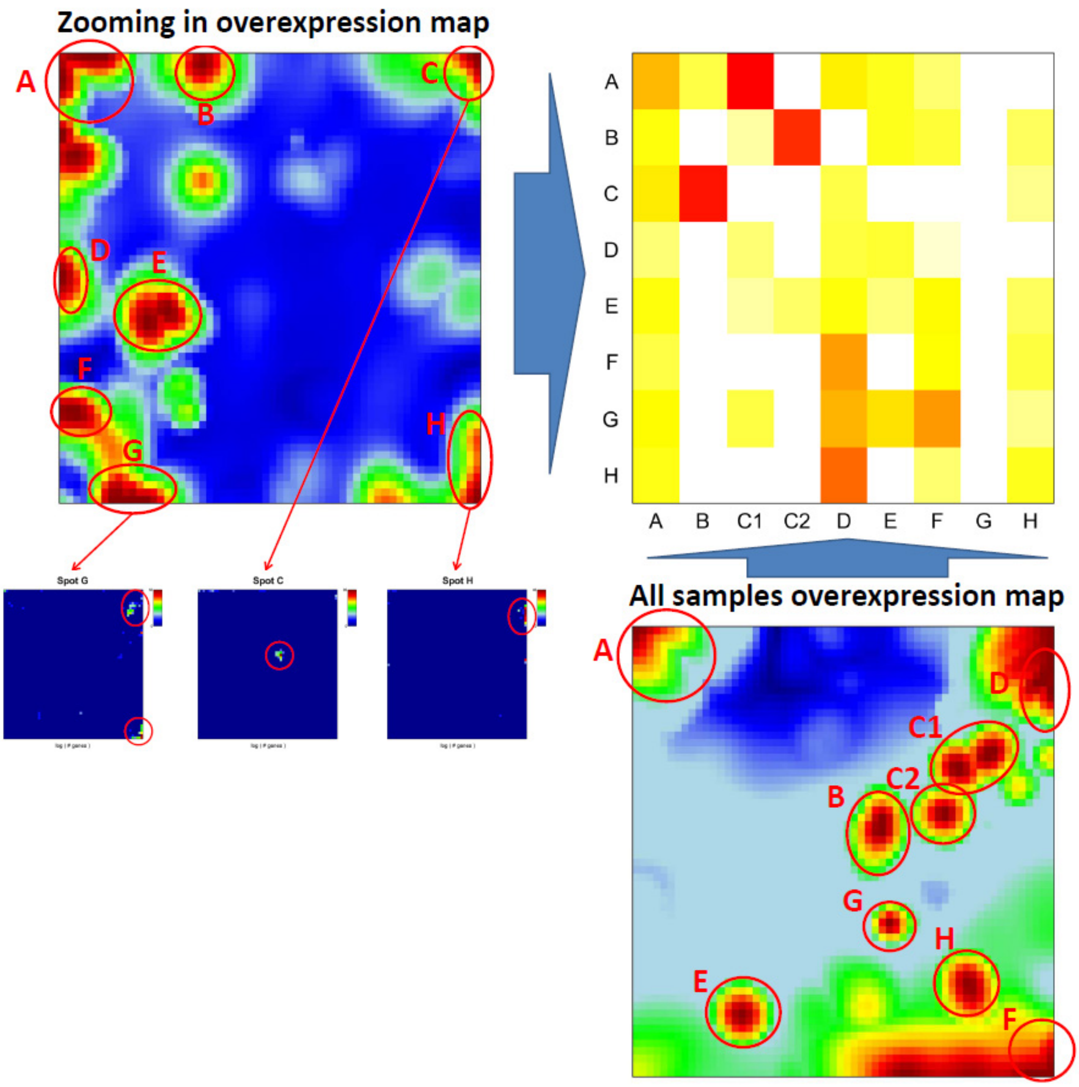

Figure S 30: Spot correspondence between the original overexpression map referring to all tissues ('all samples overexpression map') and the zoom-in overrepresentation map generated from 31 diverse tissues only. The heatmap in the top right part of the figure color codes the degree of overlap between all pairwise combination of spots from both maps (red: >50%; orange:> 10%; yellow: 1%>; white: 0%). The three small overrepresentation maps in the left part illustrate the redistribution of genes from selected spots of the zoom-in map into the total map.

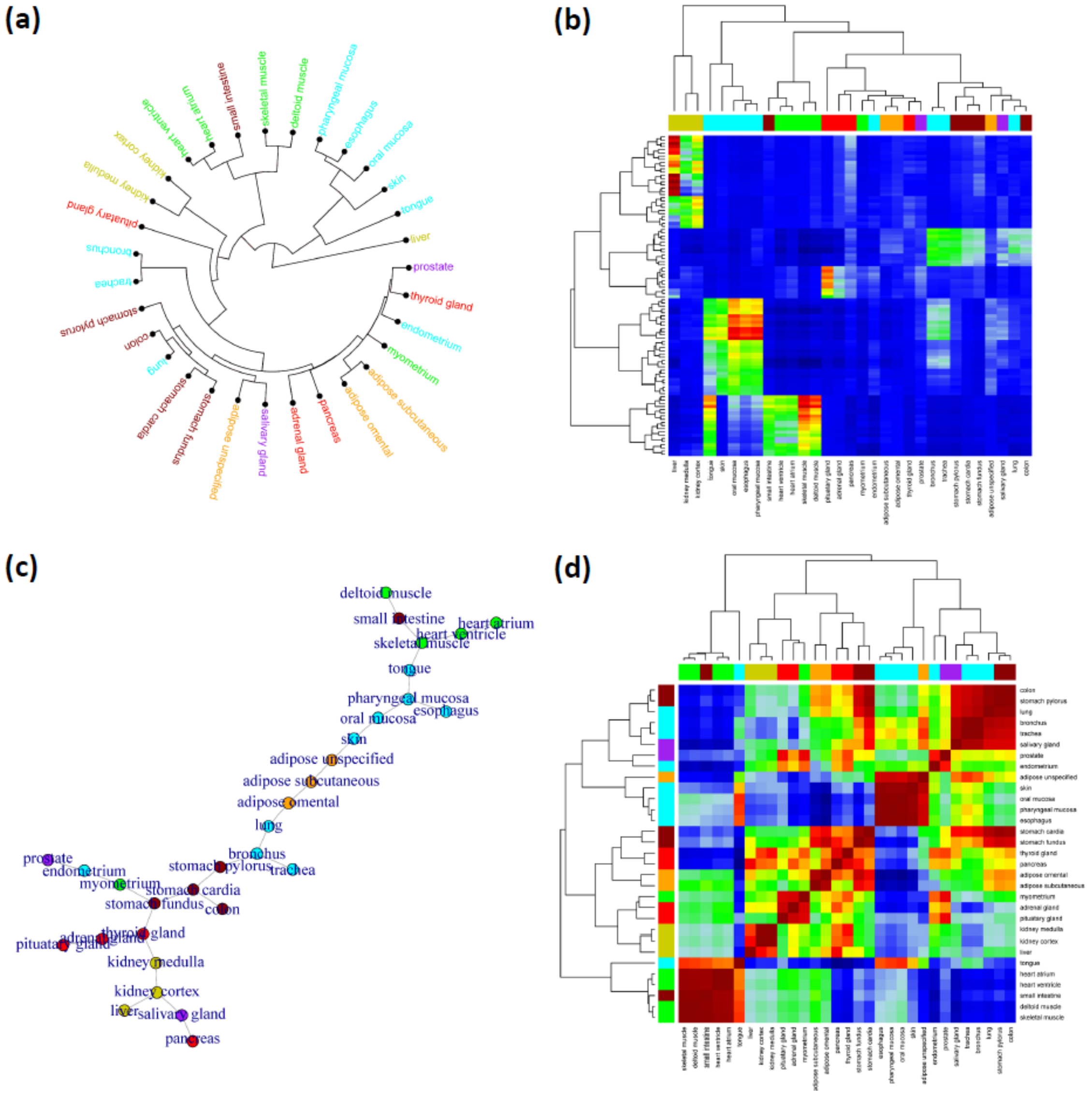

Figure S 31: Zooming-in characteristics of the group of diverse tissues: Clustering tree (panel a), two-way hierarchical clustering heatmap (b), MST (c) and PCM (d) expression fingerprints of 31 diverse tissues from 6 tissue categories based on the 100 topmost differentially expressed metagenes. The different categories are color coded as assigned by the colors of the text of the captions in Figure S 27.

15. 2nd level SOM and 3D ICA maps

Figure S 31 shows the second level SOM of all 67 human tissues studied using a coarse 9x9 grid: Each tissue is represented by its tissue number and the color of its previously assigned tissue category. In addition, representative first-level SOMs are shown in each of the occupied tiles representing the respective metasample. Note that second level SOM use a resolution where the number of mosaic tiles exceeds the number of samples. In consequence most tiles remain empty.

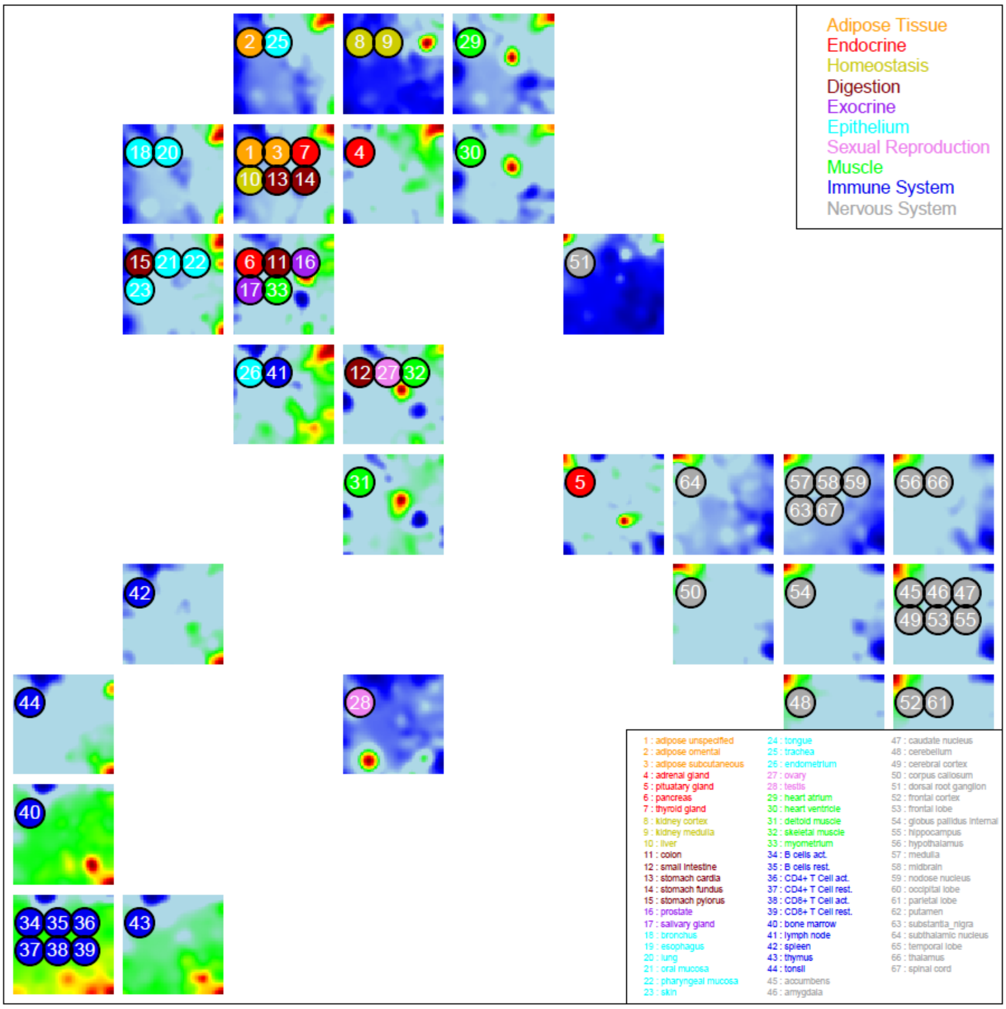

Figure S 32: Second level SOM of the metagene expression profiles of all 67 samples: Each tissue is color-coded by the circles according to its tissue category and assigned by its number. The small mosaics show the relevant first level SOM pattern of the not-empty metasamples which might be occupied by up to six real samples.

We also generated three dimensional ICA-plots to assess the third main component of variability. This plot reveals that the characteristic pattern of orthogonal linear clusters of selected tissue categories extends into the third dimension (see, e.g. the clusters of nervous system, immune system and epithelium tissues in the 3D-ICA of all tissues). Hence, the metagene-based ICA plots in two and three dimensions allow to disentangle tissue categories of virtually independent expression profiles. The responsible groups of genes can be identified using the spot pattern of the original SOM where they typically aggregate into metagene spots specifically overexpressed in the respective tissue category.

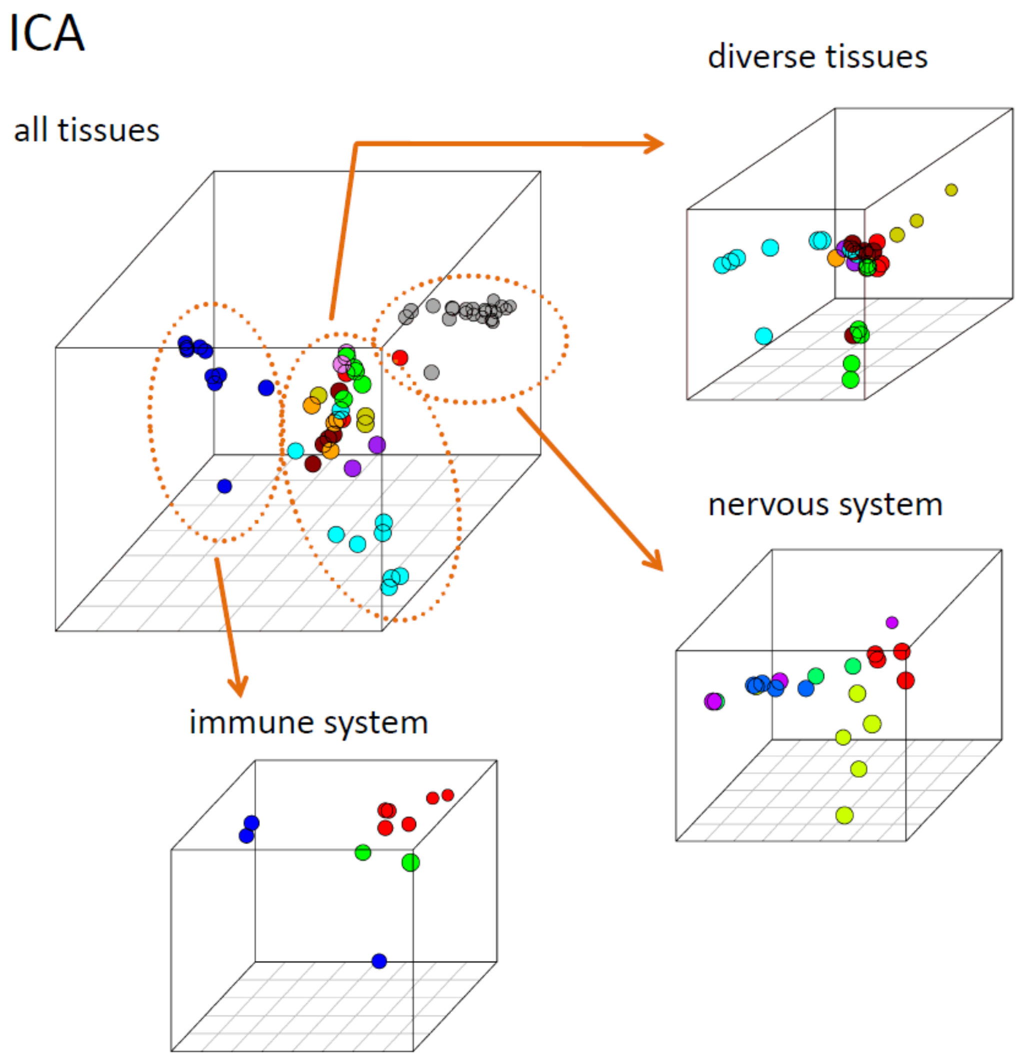

Figure S 33: Three-dimensional ICA plots of the tissues studied. Zoom-in views are shown in the right part of the figure.

16. References

[1] Nikkilä J, Törönen P *et al*: **Analysis and visualization of gene expression data using Self-Organizing Maps**. *Neural Networks* 2002, **15**(8-9):953-966.

[2] Kadota K, Nakai Y *et al*: **A weighted average difference method for detecting differentially expressed genes from microarray data**. *Algorithms Mol Biol* 2008, **3**:8.

[3] Durbin BP, Hardin JS *et al*: **A variance-stabilizing transformation for gene-expression microarray data**. *Bioinformatics* 2002, **18 Suppl 1**:S105-110.

[4] Kadota K, Nakai Y *et al*: **Ranking differentially expressed genes from Affymetrix gene expression data: methods with reproducibility, sensitivity, and specificity**. *Algorithms Mol Biol* 2009, **4**:7.

[5] Pereira V, Waxman D *et al*: **A Problem With the Correlation Coefficient as a Measure of Gene Expression Divergence**. *Genetics* 2009, **183**(4):1597-1600.

[6] Tsigelny IF, Kouznetsova VL *et al*: **Analysis of Metagene Portraits Reveals Distinct Transitions During Kidney Organogenesis**. *Sci Signal* 2008, **1**(49):ra16-.

[7] Strimmer K: **A unified approach to false discovery rate estimation**. *BMC Bioinformatics* 2008, **9**(1):303.

[8] Lee DD, Seung HS: **Learning the parts of objects by non-negative matrix factorization**. *Nature* 1999, **401**(6755):788-791.

[9] Brunet J-P, Tamayo P *et al*: **Metagenes and molecular pattern discovery using matrix factorization**. *Proceedings Of The National Academy Of Sciences Of The United States Of America* 2004, **101**(12):4164-4169.

[10] Kim PM, Tidor B: **Subsystem Identification Through Dimensionality Reduction of Large-Scale Gene Expression Data**. *Genome Res* 2003, **13**(7):1706-1718.

[11] Eisen MB, Spellman PT *et al*: **Cluster analysis and display of genome-wide expression patterns**. *Proceedings Of The National Academy Of Sciences Of The United States Of America* 1998, **95**(25):14863-14868.

[12] Läuter J, Glimm E *et al*: **Search for relevant sets of variables in a high-dimensional setup keeping the familywise error rate**. *Statistica Neerlandica* 2005, **59**:298-312.

[13] Läuter J, Horn F *et al*: **High-dimensional data analysis: Selection of variables, data compression and graphics - Application to gene expression**. *Biometrical Journal* 2009, **51**(2):235-251.

[14] Quackenbush J: **Microarrays--Guilt by Association**. *Science* 2003, **302**(5643):240-241.

[15] Wirth H, Loeffler M *et al*: **SOM-cartography of human tissues – differential expression analysis and integrating concepts of molecular function** 2011, **submitted**.
