# Supplementary material for: Expression cartography of human tissues using self organizing maps
Source: BMC Bioinformatics. 2011 Jul 27;12:306. doi: 10.1186/1471-2105-12-306 (PMC3161046; doi:10.1186/1471-2105-12-306)
Supplement: Additional file 3 — Expression profiles of human tissues in alternative color scales. [file 1471-2105-12-306-S3.PDF]

WAD Profiles

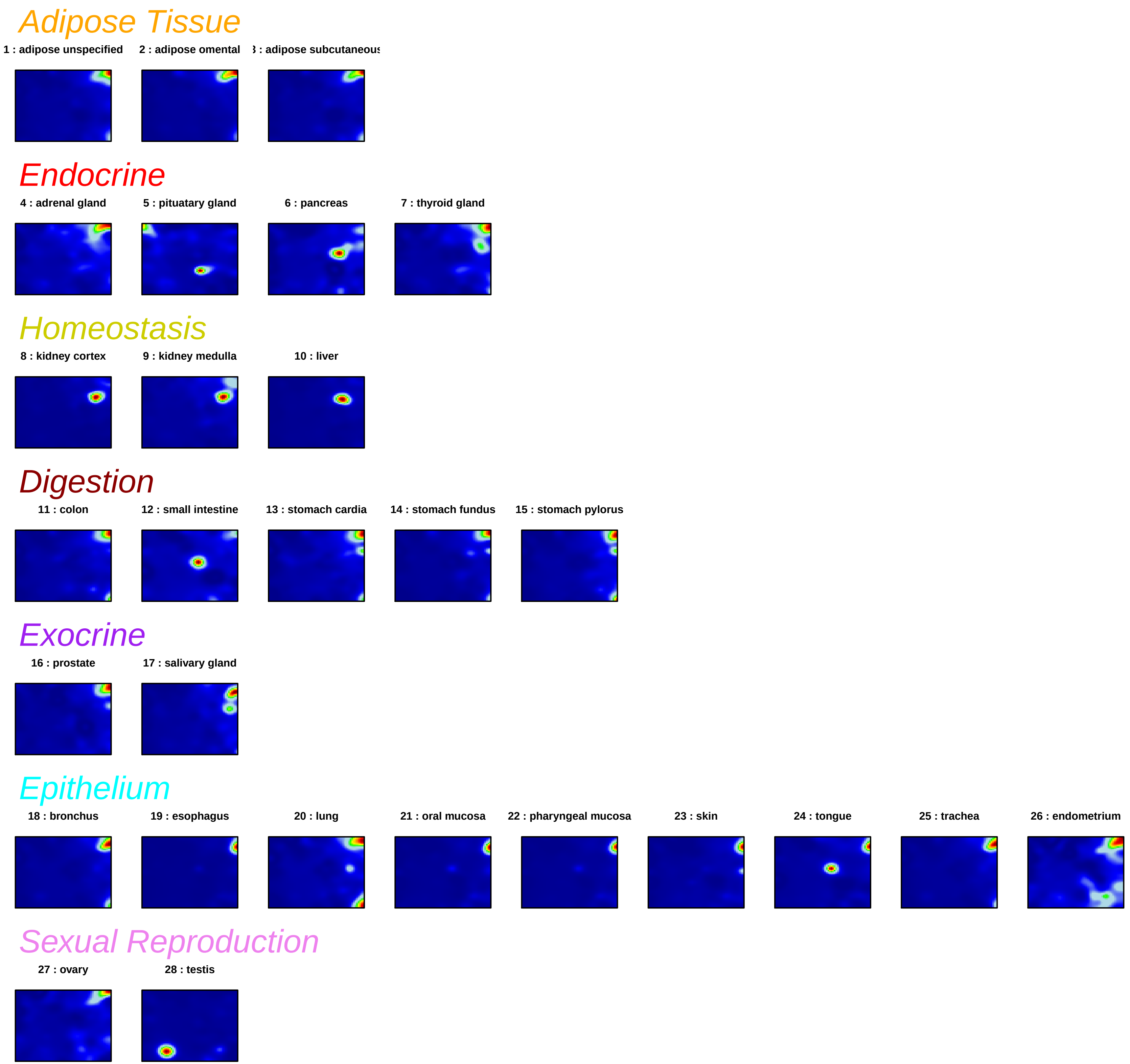
Adipose Tissue
1 : adipose unspecified
2 : adipose omental
3 : adipose subcutaneous
Endocrine
4 : adrenal gland
5 : pituatary gland
6 : pancreas
7 : thyroid gland
Homeostasis
8 : kidney cortex
9 : kidney medulla
10 : liver
Digestion
11 : colon
12 : small intestine
13 : stomach cardia
14 : stomach fundus
15 : stomach pylorus
Exocrine
16 : prostate
17 : salivary gland
Epithelium
18 : bronchus
19 : esophagus
20 : lung
21 : oral mucosa
22 : pharyngeal mucosa
23 : skin
24 : tongue
25 : trachea
26 : endometrium
Sexual Reproduction
27 : ovary
28 : testis

Muscle

29 : heart atrium | 30 : heart ventricle | 31 : deltoid muscle | 32 : skeletal muscle | 33 : myometrium

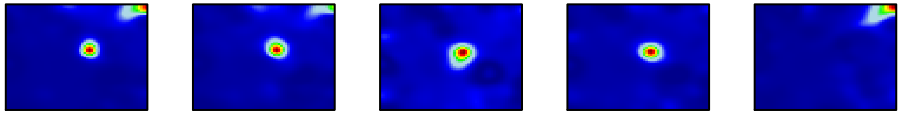

Immune System

34 : B cells act. | 35 : B cells rest. | 36 : CD4+ T Cell act. | 37 : CD4+ T Cell rest. | 38 : CD8+ T Cell act. | 39 : CD8+ T Cell rest. | 40 : bone marrow | 41 : lymph node | 42 : spleen | 43 : thymus | 44 : tonsil

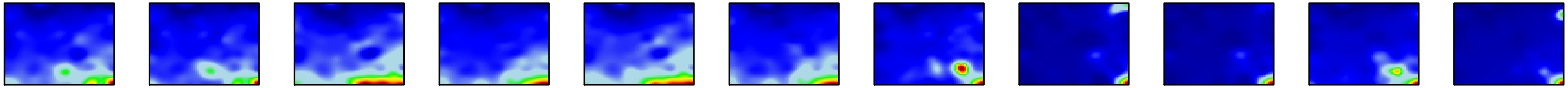

Nervous System

45 : accumbens | 46 : amygdala | 47 : caudate nucleus | 48 : cerebellum | 49 : cerebral cortex | 50 : corpus callosum | 51 : dorsal root ganglion | 52 : frontal cortex | 53 : frontal lobe | 4 : globus pallidus intern | 55 : hippocampus | 56 : hypothalamus

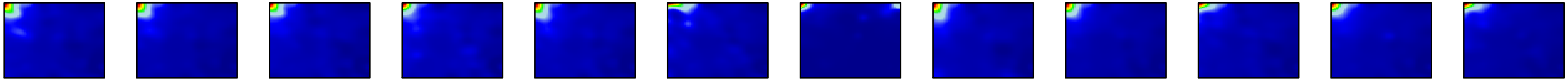

57 : medulla | 58 : midbrain | 59 : nodose nucleus | 60 : occipital lobe | 61 : parietal lobe | 62 : putamen | 63 : substantia_nigra | 64 : subthalamic nucleus | 65 : temporal lobe | 66 : thalamus | 67 : spinal cord

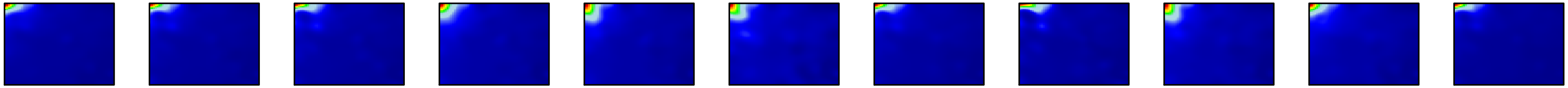

loglog FC Profiles

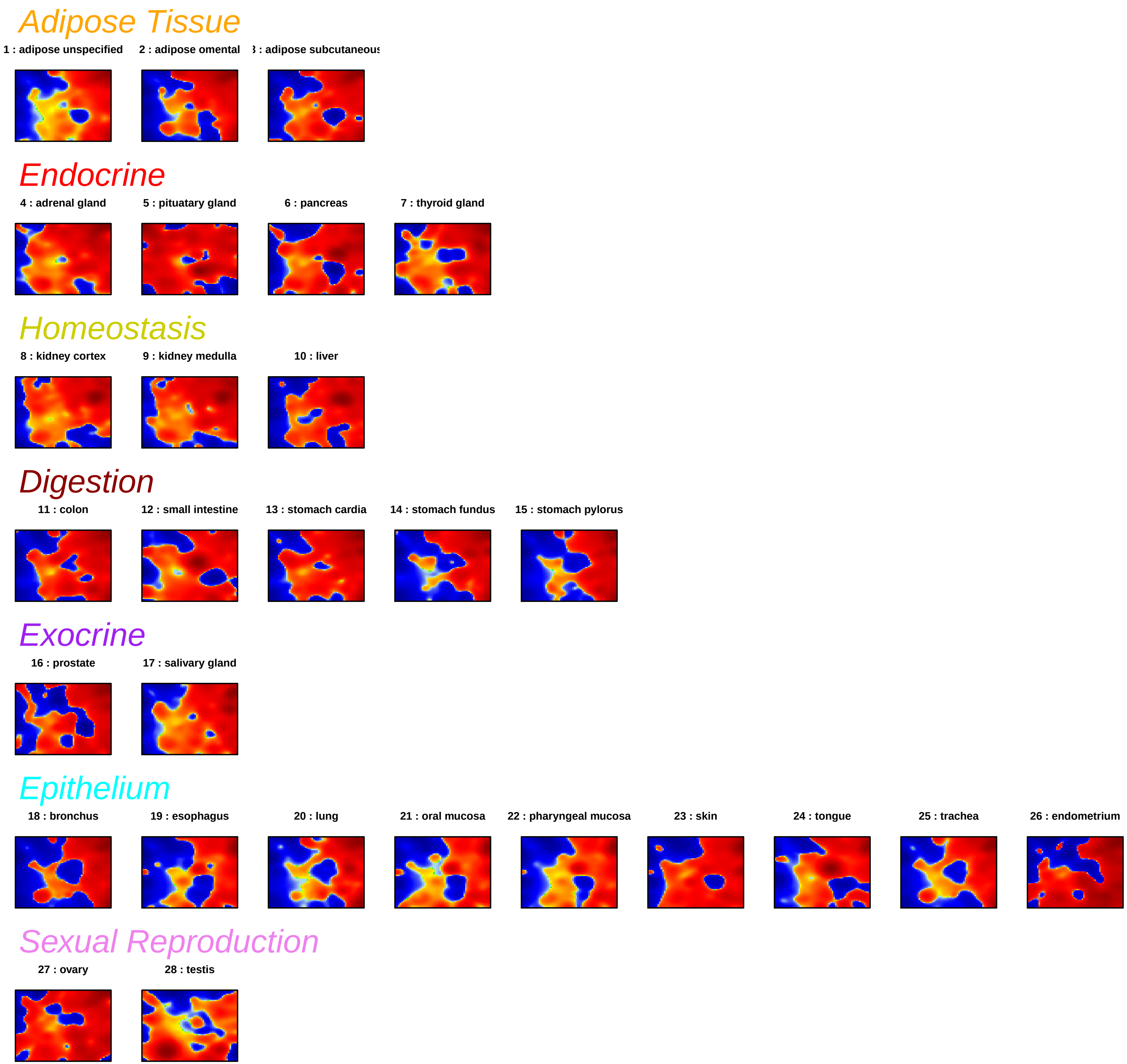
Adipose Tissue
1 : adipose unspecified
2 : adipose omental
3 : adipose subcutaneous
Endocrine
4 : adrenal gland
5 : pituatary gland
6 : pancreas
7 : thyroid gland
Homeostasis
8 : kidney cortex
9 : kidney medulla
10 : liver
Digestion
11 : colon
12 : small intestine
13 : stomach cardia
14 : stomach fundus
15 : stomach pylorus
Exocrine
16 : prostate
17 : salivary gland
Epithelium
18 : bronchus
19 : esophagus
20 : lung
21 : oral mucosa
22 : pharyngeal mucosa
23 : skin
24 : tongue
25 : trachea
26 : endometrium
Sexual Reproduction
27 : ovary
28 : testis

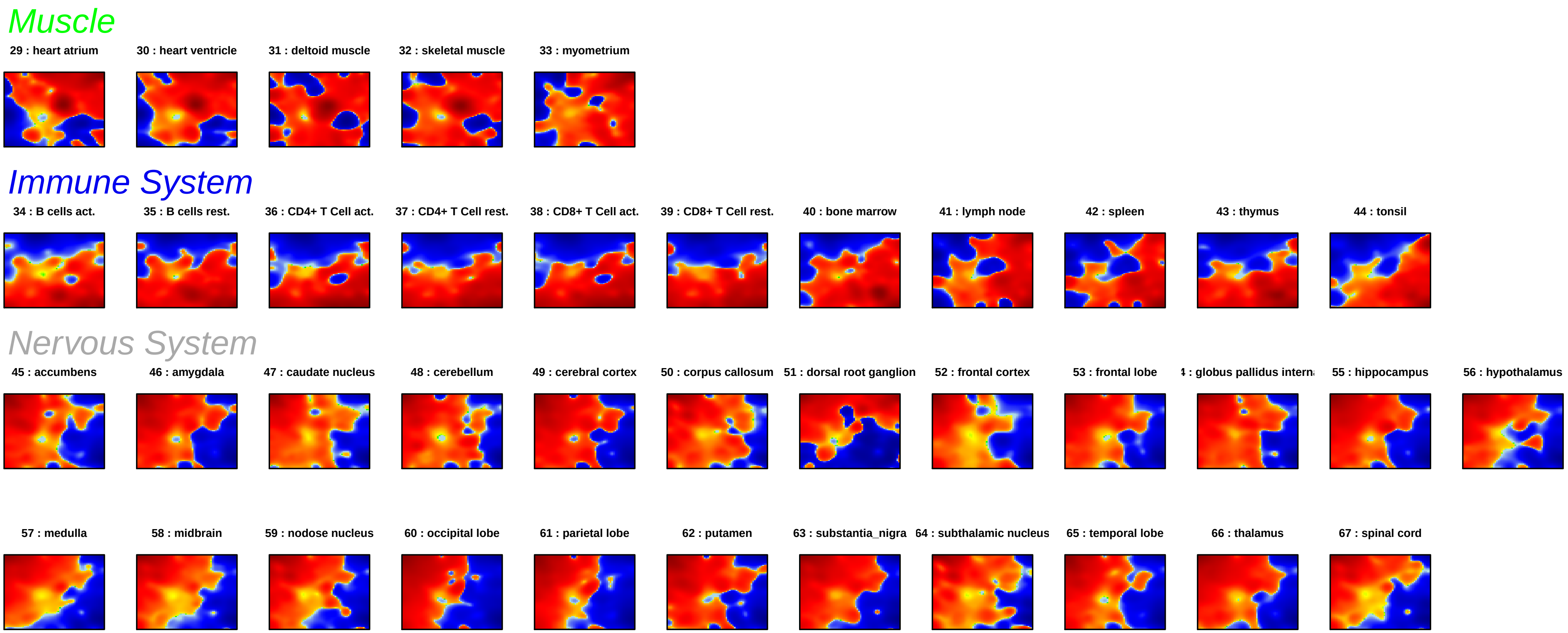
Muscle
29 : heart atrium
30 : heart ventricle
31 : deltoid muscle
32 : skeletal muscle
33 : myometrium
Immune System
34 : B cells act.
35 : B cells rest.
36 : CD4+ T Cell act.
37 : CD4+ T Cell rest.
38 : CD8+ T Cell act.
39 : CD8+ T Cell rest.
40 : bone marrow
41 : lymph node
42 : spleen
43 : thymus
44 : tonsil
Nervous System
45 : accumbens
46 : amygdala
47 : caudate nucleus
48 : cerebellum
49 : cerebral cortex
50 : corpus callosum
51 : dorsal root ganglion
52 : frontal cortex
53 : frontal lobe
4 : globus pallidus intern
55 : hippocampus
56 : hypothalamus
57 : medulla
58 : midbrain
59 : nodose nucleus
60 : occipital lobe
61 : parietal lobe
62 : putamen
63 : substantia_nigra
64 : subthalamic nucleus
65 : temporal lobe
66 : thalamus
67 : spinal cord
