# Supplementary material for: Expression cartography of human tissues using self organizing maps
Source: BMC Bioinformatics. 2011 Jul 27;12:306. doi: 10.1186/1471-2105-12-306 (PMC3161046; doi:10.1186/1471-2105-12-306)
Supplement: Additional file 4 — Agglomerative cluster analyses after single gene and metagene filtering using FDR and variance criteria [file 1471-2105-12-306-S4.PDF]

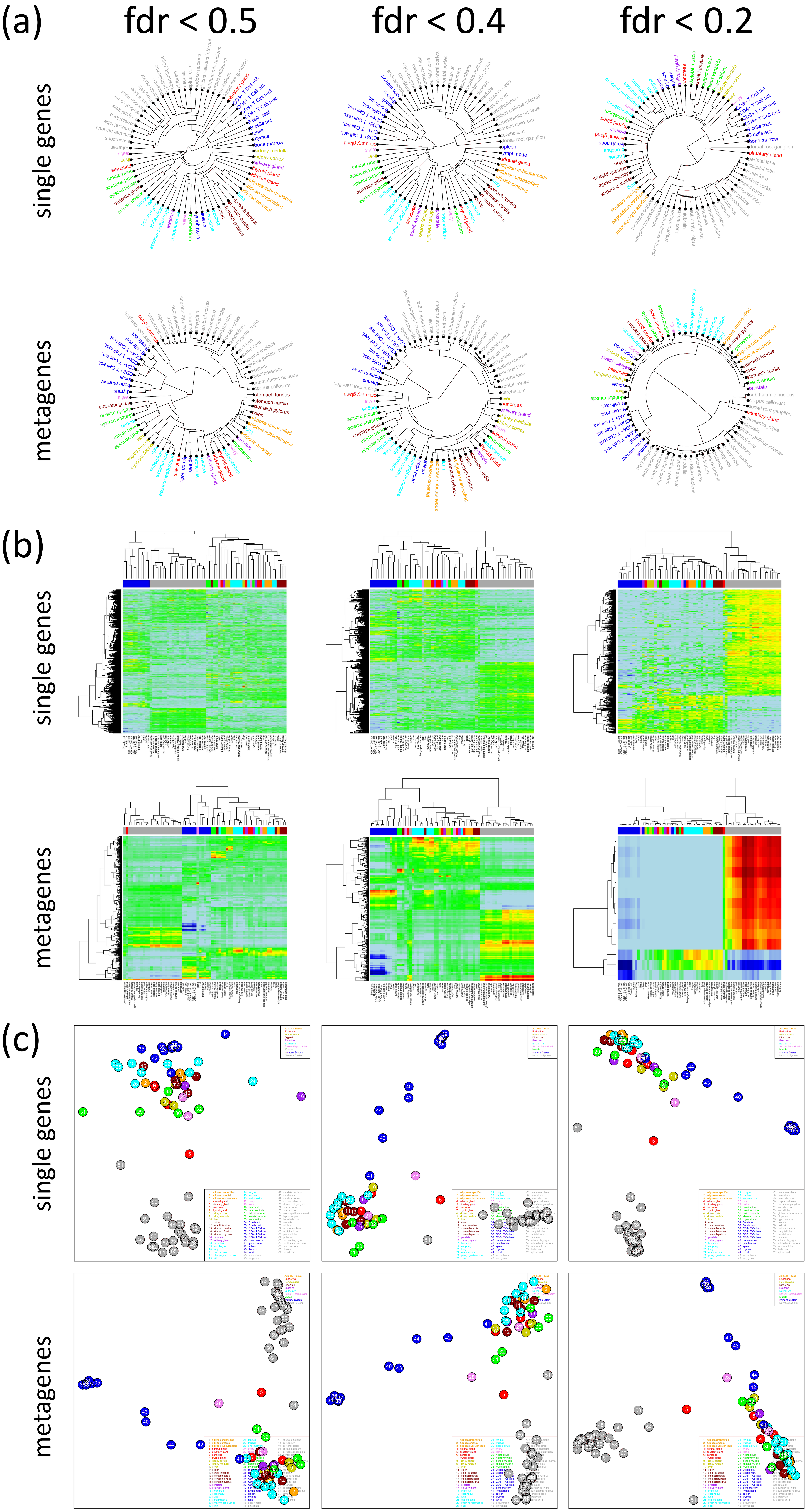
(a)
fdr < 0.5
fdr < 0.4
fdr < 0.2
single genes
metagenes
(b)
single genes
metagenes
(c)
single genes
metagenes

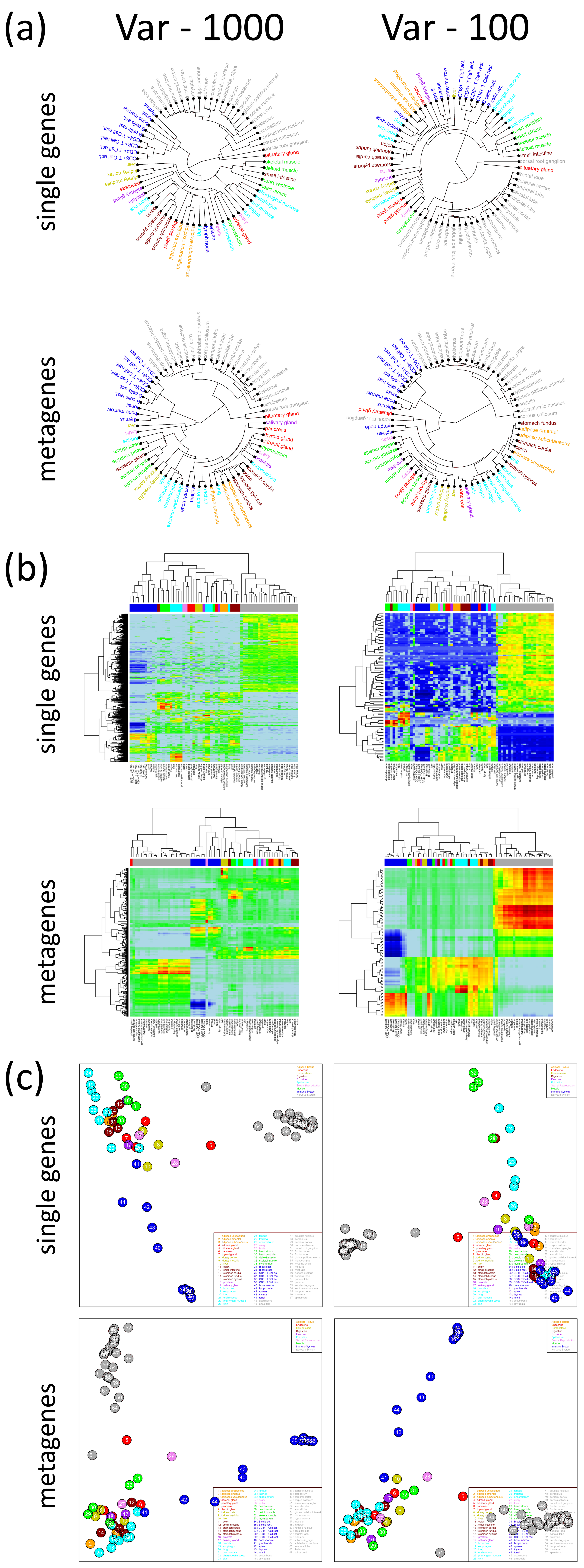
(a)
Var - 1000
Var - 100
single genes
metagenes
(b)
single genes
metagenes
(c)
single genes
metagenes
